# Modulation of O-GlcNAc cycling influences α-synuclein amplification, degradation, and associated neuroinflammatory pathology

**DOI:** 10.1186/s13024-025-00904-2

**Published:** 2025-10-27

**Authors:** Yongzhen Miao, Ting Zhang, Zhuoya Ma, Huanhuan Du, Qipei Gu, Mengni Jiang, Kangping Xiong, Chun-Feng Liu, Hongrui Meng

**Affiliations:** 1 Institute of Neuroscience, Soochow University, Suzhou, Jiangsu 215123 China; 2 Department of Neurology, Second Affiliated Hospital of Soochow University, Suzhou, Jiangsu 215004 China

**Keywords:** α-synuclein, O-GlcNAcylation, Neuroinflammation, Proteolysis

## Abstract

**Background:**

The accumulation and propagation of α-synuclein (α-syn) are hallmark features of Parkinson’s disease (PD) and related neurodegenerative disorders. O-GlcNAcylation, an abundant post-translational modification throughout the brain, is regulated by the enzymatic activity of the cycling enzymes O-GlcNAc transferase (OGT) and O-GlcNAcase (OGA) and has been implicated in altering α-syn toxicity. Nevertheless, the interplay between modulating O-GlcNAc cycling and α-syn aggregation and the propagation of amyloid pathology is not well elucidated.

**Methods:**

To this end, we delivered conformational strains of α-syn in the striatum of mice or neuronal and microglial co-cultured cells following pharmacologically or genetically inhibited OGT and OGA. The substantia nigra was injected with an adeno-associated viral vector coding for α-syn combined with α-syn preformed fibrils to examine α-syn-induced dopaminergic cytotoxicity. The α-syn pathology and spreading, protein O-GlcNAcylation, OGT and OGA levels, microglial inflammation, and behavioral impairments were evaluated. Furthermore, the O-GlcNAc modification and proteolysis status of α-syn under O-GlcNAc cycling modification were also assessed using a combination of approaches, including Click-iT™ O-GlcNAc enzyme labeling, sWGA pulldown, HPLC-MS/MS, and immunohistochemical analysis following proteasome and autophagy-lysosome inhibition.

**Results:**

We found that modulation of O-GlcNAc cycling, governed by the two enzymes OGT and OGA, significantly affected α-syn aggregation, propagation, dopaminergic neuronal degeneration, and microglial inflammation. Pathological α-syn transmission to adjacent cells and anatomically connected brain regions was found to suppress recipient cellular O-GlcNAc levels, concomitant with reduced OGT expression. Pharmacological inhibition or genetic knockdown of OGT exacerbated α-syn aggregation, enhanced its intercellular transmission, and intensified NOD-, LRR-, and pyrin domain-containing 3 (NLRP3)-mediated microglial inflammation. Conversely, increasing O-GlcNAcylation via OGA inhibition ameliorated these pathological processes. Furthermore, we demonstrate that enzymatic O-GlcNAcylation significantly regulates the aggregation of fibril-induced initial dimer formation and facilitates the clearance of α-syn aggregates through autophagosome-lysosome flux.

**Conclusions:**

These findings highlight the critical regulatory role of O-GlcNAc modification in α-syn pathology and conformational strain formation, and provide mechanical evidence that enhancing O-GlcNAc modifications alleviates pathological α-syn proteolysis by restoring autophagosome-lysosome flux.

**Supplementary Information:**

The online version contains supplementary material available at 10.1186/s13024-025-00904-2.

## Introduction

Parkinson’s disease (PD) is a progressive neurodegenerative disorder primarily characterized by the degeneration of dopaminergic neurons in the substantia nigra pars compacta (SNpc) and a subsequent decline in dopaminergic innervation [1, 2]. A growing body of evidence highlights the pivotal role of α-synuclein (α-syn), a presynaptic neuronal protein, in PD pathology [3, 4]. Under pathological conditions, α-syn aggregates into neuronal amyloid inclusions, known as Lewy bodies (LBs) and Lewy neurites (LNs), which are cytotoxic and contribute to neuronal degeneration and death [5]. These inclusions, primarily composed of aggregated α-syn, also contain proteins associated with proteostasis, such as ubiquitin [6, 7].

Amyloid inclusions originating from monomeric α-syn assemble into oligomeric intermediates, which eventually develop into polymeric fibrils [8]. These fibrils can be released by neurons and internalized by adjacent neurons or microglia, facilitating cell-to-cell transmission and microglial activation [9]. This process contributes to neurotoxicity, neuroinflammation, and disease progression. Microglial interactions with α-syn aggregates have emerged as critical contributors to α-syn pathology [10–12]. For instance, α-syn accumulation in microglia induces phagocytic dysfunction and promotes the secretion of proinflammatory cytokines, exacerbating dopaminergic neuronal damage [13]. Pharmacological inhibition of microglial activation has been shown to mitigate preformed fibril (PFF)-induced α-syn seeding and dopaminergic neuron loss [14]. Furthermore, fibrillar α-syn uptake by microglia activates the NLRP3 inflammasome, driving cytokine production and neuroinflammation [15, 16].

The progression of α-syn pathology is influenced by its structural diversity, with oligomers and fibrils displaying varying degrees of toxicity and seeding capacity [17, 18]. Amyloid fibrils derived from monomeric α-syn efficiently seed aggregation of endogenous α-syn in neurons, leading to detergent-insoluble aggregates that recapitulate PD-associated dopaminergic neurodegeneration and motor dysfunction. Post-translational modifications of α-syn have been identified as critical modulators of its aggregation, clearance, and pathogenicity, offering potential therapeutic targets to slow disease progression [19–21].

O-linked β-N-acetylglucosaminylation (O-GlcNAcylation) is a dynamic post-translational modification that plays a critical role in regulating diverse biological processes, including transcription [22], protein homeostasis [23], and metabolism [24, 25] under both physiological and pathological conditions. O-GlcNAcylation depends on a cycling process mediated by two unique intracellular enzymes: O-GlcNAc transferase (OGT), which catalyzes the addition of O-GlcNAc from UDP-GlcNAc to serine/threonine residues of target proteins, and O-GlcNAcase (OGA), which removes this modification [26]. Previous studies have demonstrated that site-specific O-GlcNAc modification of α-syn inhibits its aggregation, reduces its toxicity in cultured cells, and diminishes its conversion to amyloid fibrils [27–29]. Upregulation of O-GlcNAcylation by deleted OGA or administration of the selective OGA inhibitor Thiamet G (TMG) in animals reduces α-syn A53T mutant aggregation and motor deficits [13]. TMG treatment significantly reduces autophagic flux and proteasome activity, thereby contributing to protein accumulation [30, 31]. Additionally, OGT deletion in adult mouse brains leads to glial cell proliferation, inflammation, and neuronal apoptosis, indicating that cellular O-GlcNAc modification protects neuronal survival and mitigates glial inflammation [32]. Enhancing O-GlcNAcylation pharmacologically has been shown to reduce neurodegeneration, neuroinflammation, and motor deficiencies in 6-hydroxydopamine (6-OHDA)-induced PD models [33].

In this study, we demonstrate that fibrillar α-syn downregulate cellular O-GlcNAcylation and OGT protein levels, indicating a pathway by which α-syn aggregation exerts cytotoxic effects. Both pharmacological and genetic modulation of O-GlcNAc cycling affect the fibrillar recruitment of endogenous α-syn, promoting the formation of higher molecular weight aggregates. A decrease in O-GlcNAcylation exacerbates α-syn aggregation, resulting in dopaminergic innervation loss, α-syn transmission, and microglial activation. Conversely, upregulating O-GlcNAcylation attenuate these pathological processes. In addition, the protective effects of O-GlcNAcylation on α-syn pathology are abolished when autolysosome flux is inhibited, suggesting that O-GlcNAcylation influences α-syn fibril seeding pathology through mechanisms involving autolysosome-mediated degradation. These findings reveal a critical role for O-GlcNAc modification in modulating α-syn aggregation and pathology and support its potential as a therapeutic target for altering the progression of neurodegenerative diseases, including PD.

## Results

### α-Syn fibril seeding downregulates O-GlcNAcylation in cells and mouse brain

**Fig. 1:**
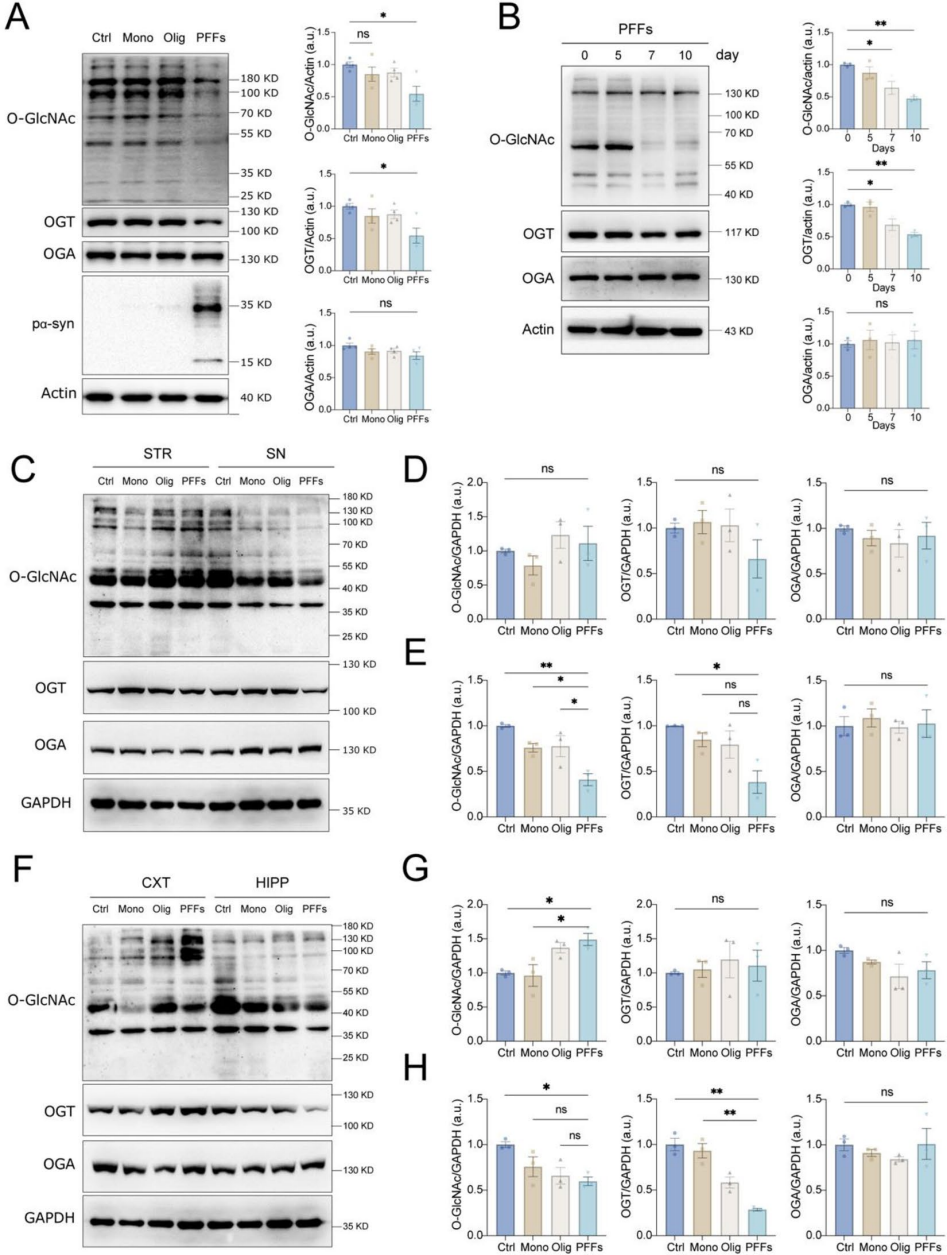
Reduced O-GlcNAc levels in SH-SY5Y cells and mouse brain in response to α-syn. (**A**) immunoblot and densitometric analysis for O-GlcNAcylation, OGT, and OGA in SH-SY5Y cells treated with 40 μg/ml of α-syn monomer (mono), oligomer (olig), and preformed fibrillar (PFFs) for five days (*n* = 4). The aggregation status of α-syn was evaluated using an anti-phosphorylated S129 α-syn (pα-syn) antibody. Actin was used as a loading control. (**B**) Representative immunoblot and quantification analysis for O-GlcNAcylation, OGT, and OGA in SH-SY5Y cells exposed to 5 μg/ml PFFs for 0, 5, 7, and 10 days. The protein levels were normalized to actin (*n* = 3). (**C**) Representative immunoblot images of O-GlcNAc, OGA, and OGT in the mouse striatum and substantia nigra tissues of α-syn bilateral striatum (5 μg/side) six months post-injection (MPI). (**D**-**E**) quantification of O-GlcNAc, OGA, and OGT levels in the striatum (**D**) and substantia nigra (**E**) normalized to GAPDH (*n* = 3). (**F**) Representative immunoblot images of O-GlcNAc, OGA, and OGT from the mouse cortex and hippocampus. (**G**-**H**) quantification of O-GlcNAc, OGA, and OGT proteins in the cortex (**G**) and hippocampus (**H**) normalized to GAPDH (*n* = 3). Data are presented as means ± SEM. *p < 0.05 and **p < 0.01 by one-way ANOVA with Tukey’s post-hoc test

Intermediate fibrils and oligomers formed during the aggregation of α-syn monomers assemble into stable fibrils with neurodegenerative properties. To investigate whether conformational α-syn strains influence O-GlcNAcylation cycling, we generated monomers, oligomers, and PFFs from recombinant human α-syn proteins (Fig S1A-D). These α-syn species were applied to SH-SY5Y and primary neuronal cells, and total O-GlcNAcylation levels were assessed. It has been reported that fibrillar α-syn acts as a seeding agent, promoting the deposition of aggregates with high molecular weights [18]. Consistent with this, we observed a significant reduction in total O-GlcNAcylation levels upon PFFs treatment, while monomers and oligomers had no effect (Fig. 1A; Fig S1E). Notably, PFFs treatment also decreased OGT protein levels, but OGA levels remained unchanged under the three α-syn strain exposures (Fig. 1A; Fig S1E). A dose-response analysis of PFFs treatment revealed that O-GlcNAcylation and OGT levels decreased at concentrations above 10 μg/ml, with maximal effects observed at 40 μg/ml (Fig S1F). Using this optimal dose, we treated SH-SY5Y cells with 5 μg/ml PFFs for up to 10 days. From day 7 onward, a pronounced reduction in both O-GlcNAcylation and OGT levels was observed (Fig. 1B).

The transmission of misfolded α-syn across anatomically connected neurons results in the templated aggregation of α-syn in neighboring neurons, leading to degeneration in vulnerable neuronal populations, a hallmark of PD in patients and animal models [34]. To determine whether α-syn propagation affects O-GlcNAcylation in anatomically connected brain regions, we injected α-syn conformational strains into the bilateral striatum (Fig S2A) and analyzed total O-GlcNAcylation, OGA, and OGT levels in the striatum, substantia nigra, hippocampus, and cortex. Although the conformational strains of α-syn injection did not alter O-GlcNAcylation levels in the striatum (Fig. 1C, D and Fig S2B, C), PFFs injections significantly reduced O-GlcNAcylation and OGT levels in the substantia nigra at both 3- and 6-month post-injection (MPI) (Fig. 1C, E and Fig S2B, D). In the hippocampus, PFFs injections reduced O-GlcNAcylation at 3 and 6 MPI and decreased OGT levels at 6 MPI (Fig. 1F, H, and Fig S2E, G). Interestingly, in the cortex, O-GlcNAcylation was upregulated at 6 MPI, although OGT levels remained unchanged (Fig. 1F, G, and Fig S2E, F). Furthermore, α-syn aggregation was detected in the striatum, substantia nigra, cortex, and hippocampus of mice injected with PFFs (Fig S3A, B). These results suggest that pathological α-syn propagation through anatomically connected regions alters O-GlcNAcylation cycling, with region-specific effects on OGT levels. Behavioral assessments of the mice at 3 and 6 MPI (Fig S2 H-R) revealed that PFF-injected mice exhibited significant motor deficiencies and increased anxiety, consistent with the observed molecular and histopathological changes.

### O-GlcNAcylation modulates fibrillar α-syn seeding and aggregation

**Fig. 2:**
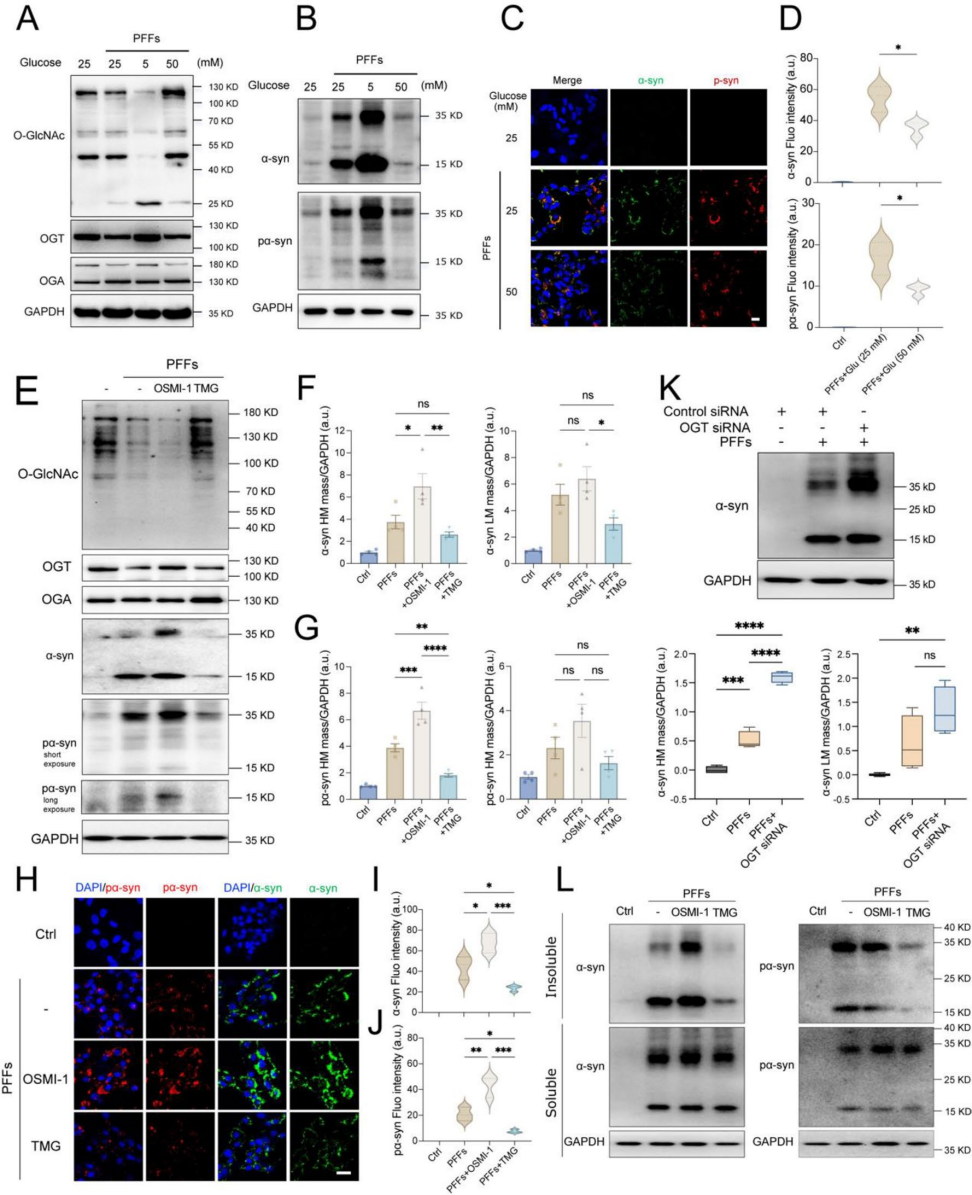
Regulating O-GlcNAc levels affects α-syn seeding aggregates in neuronal cells. (**A**-**B**) immunoblot analysis for O-GlcNAcylation, OGT, and OGA levels (**A**) and α-syn aggregation status (**B**) in SH-SY5Y cells exposed to PFFs (40 μg/ml) within medium containing glucose at concentrations of 5 mM, 25 mM, and 50 mM for five days. (**C**) immunocytochemistry of α-syn aggregates in PFFs-seeded SH-SY5Y cells cultured with varying glucose concentrations. Scale bar = 10 μm. (**D**) quantification of fluorescent intensity for α-syn and pS129 α-syn (*n* = 3). (**E**) immunoblot analysis for the effects of OSMI-1 (20 μM) or TMG (1 μM) treatment on α-syn aggregation in cells exposed to PFFs. Total O-GlcNAcylation, as well as OGT and OGA levels, were also examined. (**F**-**G**) quantification of the high molecular bands (~35 kDa) and lower molecular bands (~15 kDa) recognized by the pan α-syn (**F**) and pS129 α-syn (pα-syn) (**G**) antibodies. α-syn levels were normalized to GAPDH (*n* = 3). (**H**) immunocytochemistry of PFFs-seeded cells exposed to OSMI-1 or TMG. Scale bar = 20 μm. (**I**-**J**) quantification of fluorescent intensity of pan α-syn (**I**) and pα-syn (**J**) (*n* = 3). (**K**) immunoblot and quantitative analysis for α-syn aggregation in SH-SY5Y cells with OGT siRNA. Quantification of the high and low molecular weight bands of α-syn. The protein levels were normalized to GAPDH (*n* = 4). (**L**) immunoblot analysis of Triton X-100 soluble and insoluble fractions of α-syn in PFFs-seeded cells exposed to OSMI-1 or TMG for five days. Values are presented as means ± SEM. *p < 0.05, **p < 0.01, and *p < 0.001 by one-way ANOVA with Tukey’s post-hoc test

Previous studies have demonstrated that site-specific O-GlcNAcylation of α-syn through synthetic chemistry slows its aggregation kinetics in biochemical assays [27, 28]. These findings suggest that manipulating O-GlcNAcylation within the cellular microenvironment may modulate α-syn aggregation. One potential mechanism involves altering uridine diphosphate N-acetylglucosamine (UDP-GlcNAc) availability, the donor substrate for O-GlcNAcylation, derived from glucose flux through the hexosamine biosynthetic pathway (HBP) [35]. To investigate this, we manipulated glucose levels in cultured cells seeded with PFFs and analyzed α-syn aggregation. Reducing glucose concentration (5 mM) decreased O-GlcNAcylated proteins and increased α-syn phosphorylation at serine 129 (pS129), a hallmark of LB aggregates found in insoluble fractions [36, 37]. Immunoblotting revealed two prominent bands corresponding to high molecular weight (HMW, ~35 kDa) and low molecular weight (LMW, ~15 kDa) α-syn species, recognized by pan-α-syn (MJFR1) and pS129 antibodies. Conversely, high glucose (50 mM) increased O-GlcNAc levels and reduced both total α-syn and pS129 phosphorylation in SH-SY5Y cells (Fig. 2A, B). Interestingly, low glucose conditions increased OGT expression in the presence of PFFs, likely as a compensatory response to counteract reduced O-GlcNAc levels (Fig. 2A). Using immunofluorescence, we further validated α-syn aggregation by staining for pan-α-syn and pS129 in cultured cells. Under normal glucose conditions, phosphorylated α-syn partially colocalized with pan-α-syn, indicating PFFs-induced aggregation. High glucose culture conditions markedly reduced these signals (Fig. 2C, D).

O-GlcNAcylation relies on cycling mediated by two exclusive enzymes: OGT and OGA. Previous studies have suggested that modulating O-GlcNAcylation through pretreatment with enzyme inhibitors may influence PFFs-induced internalization of α-syn by cells [38]. To specifically assess the role of O-GlcNAcylation in α-syn aggregation, SH-SY5Y cells were first seeded with PFFs and then treated with OSMI-1 and Thiamet G (TMG), selective inhibitors of OGT and OGA, respectively [39]. OSMI-1 treatment reduced O-GlcNAcylation levels while increasing HMW total and phosphorylated α-syn (Fig. 2E–G). Moreover, PFFs-induced aggregates progressively worsen over time, decreasing the viability of SH-SY5Y cells (Fig S3C, D). This effect was further aggravated by OSMI-1 treatment but alleviated by TMG treatment (Fig S3E). Transient OGT knockdown using siRNA similarly elevated HMW α-syn levels while reducing O-GlcNAcylation levels (Fig. 2K; Fig S3F-I). These findings were corroborated by immunofluorescence in SH-SY5Y cells and primary neurons, where OSMI-1 elevated total and phosphorylated α-syn levels, whereas TMG reversed these effects (Fig. 2H–J; Fig S4A, B). Furthermore, OSMI-1 treatment increased the Triton X-100 insoluble fraction of total and pS129 α-syn, whereas TMG reduced it in cells seeded with fibrils (Fig. 2L).

### O-GlcNAc modulates pathological α-syn toxic to dopaminergic neurons

**Fig. 3:**
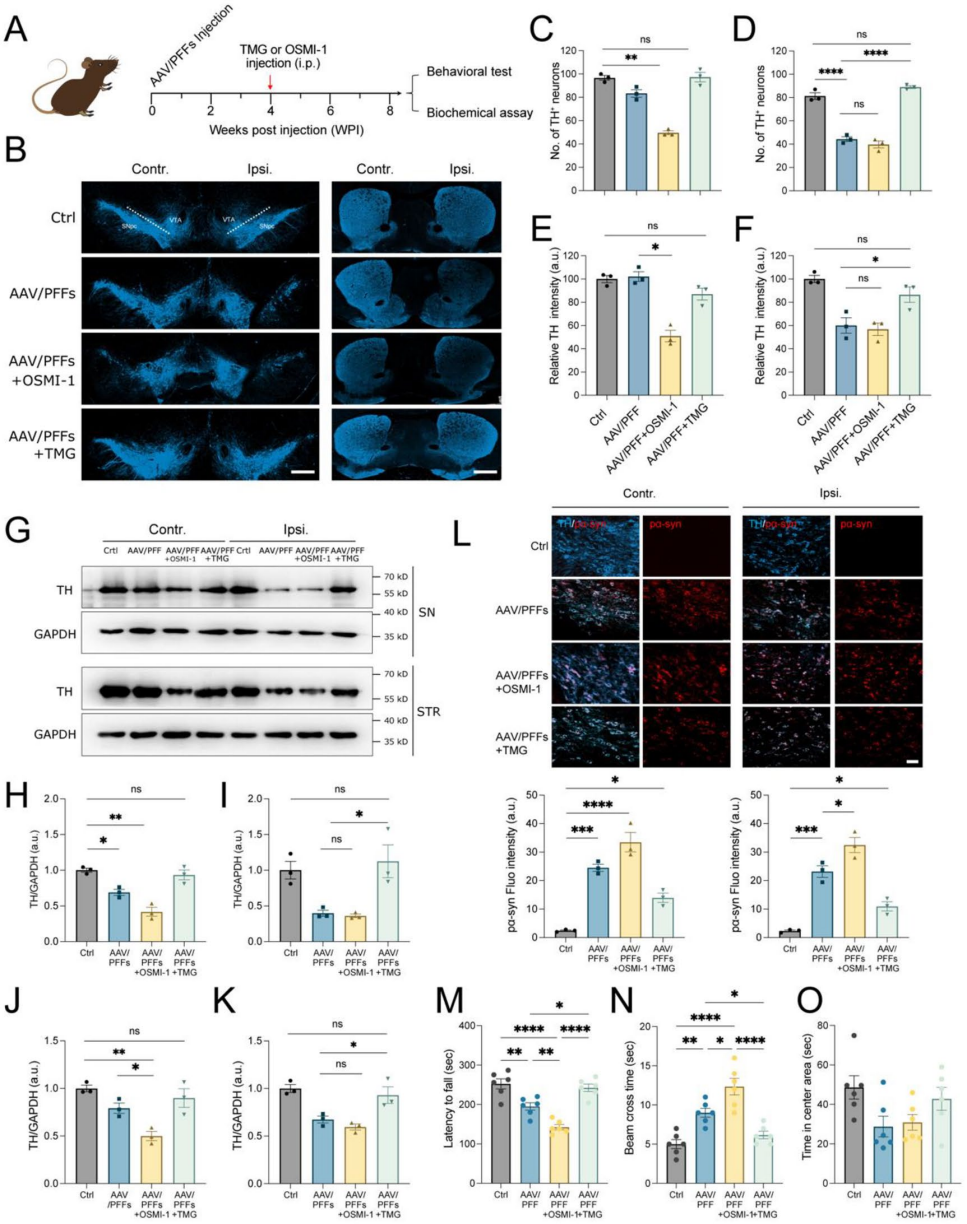
Regulating O-GlcNAcylation affects α-syn-induced dopaminergic deterioration. (**A**) schematic illustration of the experiment design in AAV/PFFs mice. A mixture of AAV-*mSNCA* (2.0 ×10^9^ va) and PFFs (10 μg) was microinjected into the right substantia nigra. Mice were treated with OSMI-1 (0.2 mg/kg) and TMG (20 mg/kg) for four weeks following the initial four weeks of the AAV/PFFs injection period. (**B**) fluorescent visualization of dopaminergic neurons and tyrosine hydrolysate (TH) expression in the substantia nigra and striatum of mouse brains using TH antibodies. Scale bar = 500 μm. (**C**-**D**) summary statistics for TH+ cell number in the contralateral side (**C**) and ipsilateral side (**D**) of the substantia nigra (*n* = 3). (**E**-**F**) summary statistics for TH fiber intensity in the contralateral side (**E**) and ipsilateral side (**F**) of the striatum (*n* = 3). (**G**) immunoblot analysis of TH levels in the tissue lysates of the substantia nigra and striatum. (**H**-**K**) quantification of TH in the contralateral side and ipsilateral side of the substantia nigra (**H** and **I**) and stratum (**J** and **K**) in AAV/PFFs mice, respectively. TH levels were normalized to GAPDH (*n* = 3). (**L**) visualization and quantification of α-syn aggregation in the dopaminergic neurons on the contralateral and ipsilateral sides of the substantia nigra in the AAV/PFFs-injected mice, using TH and pα-syn immunofluorescence staining (*n* = 3). Scale bar = 50 μm. (**M**) rotarod test, (**N**) balance beam test, and (**O**) open field test in AAV/PFFs mice following compound administration prior to sacrifice for biochemistry analysis. The corresponding parameter scores time, including latency to fall (**M**), time spent on the cross beam (**N**), and time spent in the center area (**O**), were quantified (*n* = 6). Values are presented as means ± SEM. *p < 0.05, **p < 0.01, *p < 0.001, **p < 0.0001. Statistical analysis was performed using one-way ANOVA with Tukey’s post-hoc test

The regulation of O-GlcNAcylation has been shown to critically affect α-syn seeding and aggregation, which are implicated in dopaminergic neuron toxicity and motor deficits. We speculate that OSMI-1 exacerbates neuronal degeneration associated with α-syn seeding aggregates in vivo, whereas TMG exerts a protective effect. To address this, we employed a mouse model involving the microinjection of an adeno-associated virus (AAV) vector expressing the *SNCA* gene and α-syn-derived PFFs (Fig S5A-E). This model induces accelerated α-syn pathology, dopaminergic neuron loss, and motor deficits, as previously reported [40–42]. Mice received unilateral injections into the substantia nigra of a mixture of an AAV vector overexpressing mouse α-syn (pAAV-hsyn-*mSNCA*-*eGFP*) and PFFs (AAV/PFFs), followed by intraperitoneal administration of either OSMI-1 or TMG for four consecutive weeks (Fig. 3A). Behavioral tests and biochemical analyses were conducted post-treatment. Consistent with the prior studies [42], unbiased stereological quantification revealed substantial loss of tyrosine hydroxylase (TH)-positive neurons in the ipsilateral substantia nigra following AAV/PFFs injection (Fig. 3B–D; Fig S5E). TH intensity was similarly reduced in the ipsilateral striatum, with no significant changes on the contralateral side (Fig. 3B, E, and F). OSMI-1 treatment did not exacerbate TH^+^ neuron loss in the ipsilateral substantia nigra (Fig. 3B, D), but it caused contralateral TH^+^ neuron loss (Fig. 3B, C). Moreover, OSMI-1 reduced TH fiber intensity in the contralateral striatum of AAV/PFFs-treated mice (Fig. 3B, E). Although OSMI-1 appears toxic to various cell types in vitro [43, 44], treatment with OSMI-1 alone did not cause an obvious loss of TH (Fig S4C, D). These findings indicate that OSMI-1-mediated downregulation of O-GlcNAcylation increases the vulnerability of dopaminergic neurons to PFFs-induced cytotoxicity. In contrast, TMG treatment significantly rescued ipsilateral dopaminergic neuron loss (Fig. 3B, D) and the reduction in TH intensity (Fig. 3B, F). Immunoblot analysis further confirmed that TH levels changes in these regions were consistent with the immunofluorescence results (Fig. 3G–K).

To examine the aggregation and transmission of α-syn pathology, double immunofluorescent staining for phosphorylated α-syn at serine 129 (pS129) and TH was performed. Robust perinuclear pS129 α-syn aggregates were observed in surviving dopaminergic neurons on the ipsilateral substantia nigra. OSMI-1 treatment significantly increased pS129 α-syn deposition in TH^+^ neurons on both sides of the substantia nigra, whereas TMG treatment reduced this accumulation (Fig. 3L). These findings suggest that pharmacological upregulation of O-GlcNAcylation via OGA inhibition effectively mitigates α-syn aggregation in dopaminergic neurons. Behavioral deficits, including motor impairment and anxiety, were consistent with α-syn pathology and dopaminergic degeneration. After four weeks of drug treatment, mice were subjected to locomotor, fine motor coordination, and anxiety testing. AAV/PFFs mice exhibited reduced latency to fall in the rotarod test and increased crossing time in the balance beam test, indicating motor impairment (Fig. 3M, N). OSMI-1 treatment further aggravated motor and coordination deficits, whereas TMG treatment partially rescued these deficiencies. In the open-field test, AAV/PFFs mice spent less time in the center area, indicative of anxiety-like behavior; however, no significant changes were observed with OSMI-1 or TMG treatment (Fig. 3O). These results indicate the sensitivity of dopaminergic neurons to α-syn pathology and support a protective role of O-GlcNAcylation in dopaminergic neurodegeneration.

### Enzymatic regulation of OGA and OGT mediates dimeric α-syn O-GlcNAcylation in fibril aggregation

**Fig. 4:**
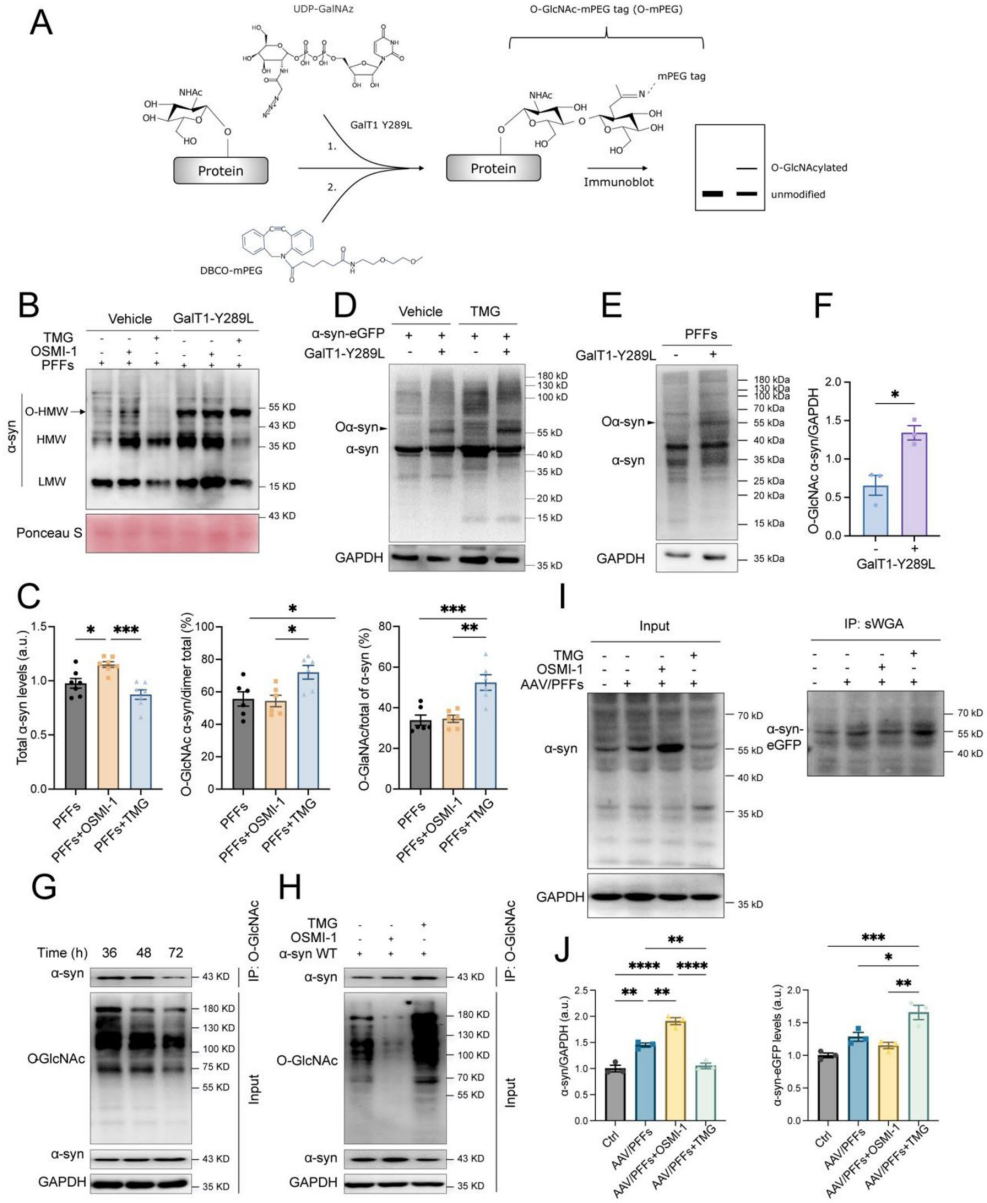
Regulating O-GlcNAcylation affects dimeric α-syn formation. (**A**) schematic illustration of the procedure for detecting O-GlcNAcylated α-syn (Oα-syn) in PFFs-seeded SH-SY5Y cells and mouse brain treated with OSMI-1 or TMG using the click-iT™ O-GlcNAc enzyme labeling system. (**B**) immunoblot analysis of O-GlcNAcylated α-syn (Oα-syn) in PFFs-seeded SH-SY5Y cells. Ponceau S staining serves as a loading control. Arrowhead indicates the Oα-syn bands that shifted from a higher molecular weight (HMW) of ~ 35 kDa (dimeric α-syn) to ~ 55 kDa in the presence of GalT1 (Y289L). (**C**) quantification of α-syn and the proportion (%) of Oα-syn in the dimeric and total α-syn in the cells treated with OSMI-1 or TMG (*n* = 6). (**D**) examination of Oα-syn in SH-SY5Y cells transfected with α-syn-eGFP plasmid. (**E**) representative immunoblot images of Oα-syn in the 6 months post-injection of PFFs. (**F**) quantification of the Oα-syn in the brain of the mice 6 months post-injection of PFFs. Oα-syn levels normalized to GAPDH (*n* = 3). (**G**-**H**) immunoblot analysis of immunoprecipitated α-syn in SH-SY5Y cells transfected with α-syn-eGFP plasmids for 36 h to 72 h (**G**) or exposed to OSMI-1 or TMG (**H**) using a pan O-GlcNAc antibody (RL2). (**I**) sWGA pull-down assay of α-syn in the brains of AAV/PFFs mice treated with OSMI-1 or TMG. (**J**) quantification of α-syn from the input and sWGA pull-down fractions. α-syn and Oα-syn were normalized to GAPDH (input) or the densitometric gray value (IP). Data are shown as means ± SEM. *p < 0.05, **p < 0.01, *p < 0.001, and **p < 0.0001 by one-way ANOVA with Tukey’s post-hoc test

Posttranslational modification, such as O-GlcNAcylation of α-syn, profoundly influences its fibrillar recruitment of soluble proteins into high-conformational strains with altered pathogenicity and toxicity [20, 29, 34]. However, the role of O-GlcNAc cycling in α-syn aggregate assembly remains incompletely understood. Using the Click-iT™ O-GlcNAc enzyme labeling system [45], a chemoenzymatic mass tagging approach, we assessed the impact of inhibiting OGA and OGT on α-syn O-GlcNAcylation after fibril seeding. This method employs click chemistry to conjugate a 5 kDa polyethylene glycol mass tag to O-GlcNAcylated residues, enabling their detection on immunoblots as higher molecular weight species compared to unmodified proteins (Fig. 4A). In lysates from SH-SY5Y cells seeded with PFFs, chemoenzymatic mass tagging revealed α-syn bands at ~ 15 kDa and ~35 kDa, as well as a prominent higher molecular weight band at ~ 55 kDa labeled with the GalT1 (Y289L) enzyme (Fig. 4B). OSMI-1 treatment increased total α-syn levels but had minimal effects on the proportion of O-GlcNAcylated α-syn. In contrast, TMG treatment significantly increased the proportion of O-GlcNAcylated α-syn at higher molecular weights while reducing total α-syn levels. Approximately 75% of dimeric α-syn and 50% of total α-syn were O-GlcNAc-modified (Fig. 4B, C). To further elucidate O-GlcNAc modification, we overexpressed α-syn via plasmid transfection under TMG treatment. Similar to endogenous monomer [46], TMG increased O-GlcNAcylation of exogenously expressed α-syn, with a theoretical molecular mass of ~ 55 kDa (Fig. 4D). In PFFs-treated mouse brains, O-GlcNAc-modified α-syn shifted to ~ 55 kDa (Fig. 4E, F), confirming dimeric α-syn O-GlcNAcylation. These findings suggest that fibrillar seeding facilitates O-GlcNAc modification during dimer assembly, and modulation of O-GlcNAc cycling significantly impacts this process.

Recent studies highlight the critical role of post-translational modifications in the aggregation of soluble monomeric α-syn [20, 47]. It is intriguing to know whether α-syn protein can be O-GlcNAcylated, so we transfected SH-SY5Y cells with *SNCA* tagged with *eGFP* and precipitated α-syn using an anti-O-GlcNAc antibody (RL2). The antibody successfully identified and precipitated α-syn. After 72 hours of α-syn induction, O-GlcNAcylation levels and precipitated α-syn were significantly reduced (Fig. 4G). We next examined the effects of OGT or OGA inhibition on the proportion of immunoprecipitated α-syn. TMG treatment significantly increased O-GlcNAcylation levels and the proportion of precipitated α-syn, while OSMI-1–treated samples maintained O-GlcNAcylation at levels comparable to untreated α-syn–expressing cells (Fig. 4H). A sWGA pull-down assay further confirmed that TMG primarily enhanced dimeric α-syn O-GlcNAcylation, with theoretically elevated molecular weights around ~55 kDa (Fig. 4I, J). These results demonstrated that OGA inhibition significantly enhances O-GlcNAc modification of α-syn during dimer formation. To identify glycosylation sites on dimeric α-syn under PFFs seeding conditions, proteins from cell lysates were separated via SDS-PAGE and analyzed by HPLC-MS/MS. Multiple post-translational modifications, including O-GlcNAcylation, phosphorylation, ubiquitination, and oxidation, were identified on dimeric α-syn (Fig S6A-B and Table S1). Notably, 12 modifications were located in the N-terminal domain, including four O-GlcNAcylation sites (Ser42, Thr44, Thr54, and Thr59) (Fig S6B), which are conserved across species (Fig S6C). These findings indicate that dimeric α-syn possesses four O-GlcNAc-modified sites, suggesting a regulatory role for O-GlcNAc cycling in fibril-associated assembly and pathology.

### Inhibition of OGA and OGT modulates α-syn transmission and NLRP3-dependent microglial inflammation

**Fig. 5:**
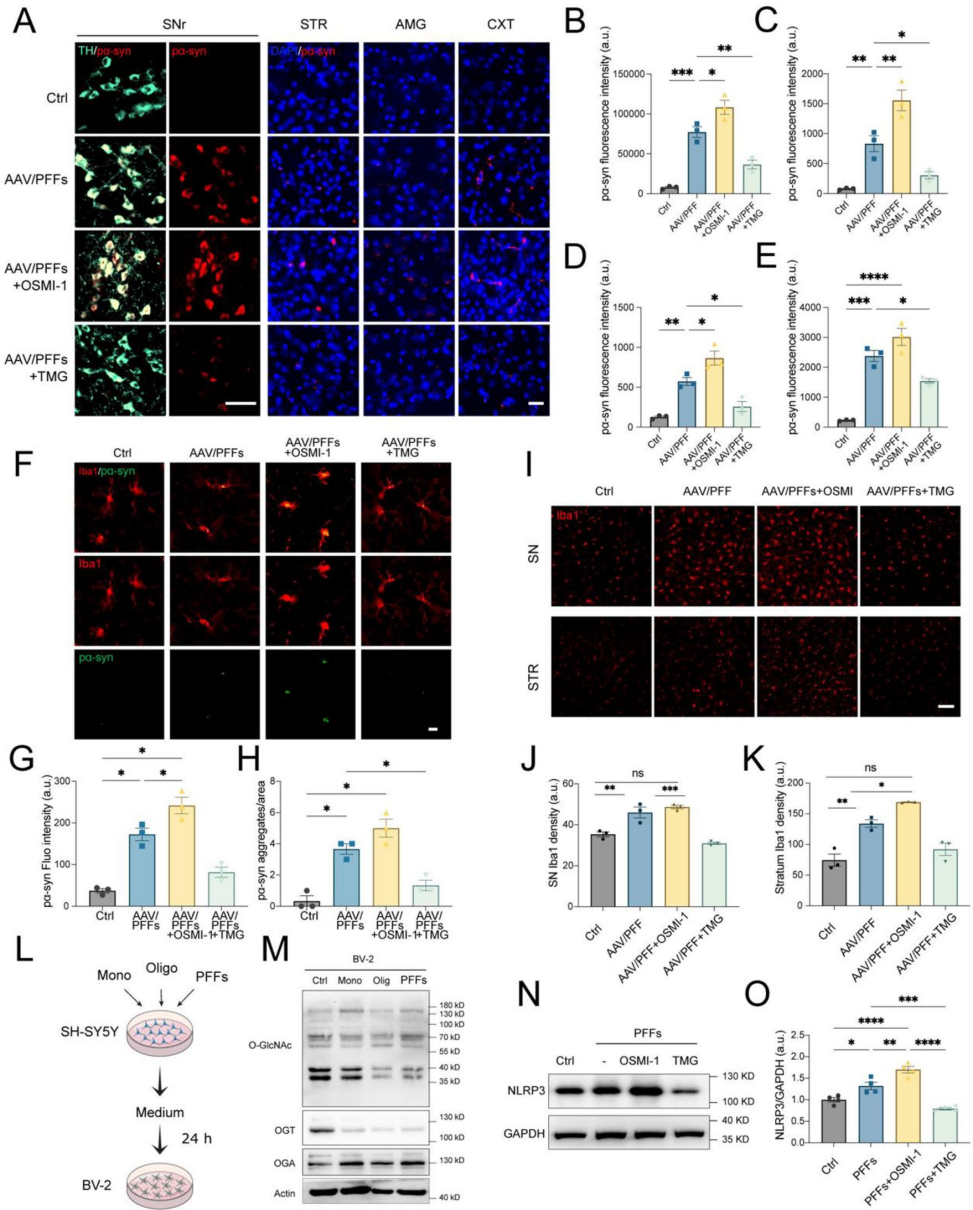
Regulating O-GlcNAc affects pathological α-syn spreading and microglial NLRP3-related inflammation. (**A**) immunohistochemistry of pα-syn (red) in dopaminergic neurons (TH^+^, green) in the substantia nigra and aggregated α-syn distribution in the striatum, amygdala, and cortex of the AAV/PFFs mice treated with OSMI-1 or TMG. Scale bars = 50 μm (left) and 25 μm (right). (**B**-**E**) quantification of fluorescent intensity of pα-syn in the substantia nigra (**B**), striatum (**C**), amygdala (**D**), and cortex (**E**). (*n* = 3). (**F**) deposition of pα-syn (green) in Iba1-marked microglia (red) from the striatum of AAV/PFFs mice. Scale bar = 10 μm. (**G**-**H**) quantification of fluorescent intensity (**G**) and number of aggregated pα-syn puncta (**H**) in microglia (*n* = 3). (**I**) immunohistochemistry of hypertrophic activated Iba1^+^ microglia in the substantia nigra and striatum of AAV/PFFs mice affected by OSMI-1 or TMG treatment. Scale bar = 25 μm. (**J**-**K**) quantification of Iba1 density of microglia in the substantia nigra (**J**) and striatum (**K**) (*n* = 3). (**L**) conditional medium (CM) from SH-SY5Y cells treated with conformational strains of α-syn was used to culture BV-2 cells for 24 h. (**M**) immunoblot analysis of O-GlcNAc, OGA, and OGT in BV-2 cells. Actin was used as a loading control. (**N**-**O**) immunoblot and quantification analysis of NLRP3 in BV-2 cells cultured with CM from PFFs-seeded SH-SY5Y cells treated with OSMI-1 or TMG. NLRP3 levels normalized to GAPDH (*n* = 4). Data are presented as means ± SEM. *p < 0.05, **p < 0.01, *p < 0.001, and **p < 0.001 by one-way ANOVA with Tukey’s post-hoc test

Pathological α-syn has been demonstrated to spread between neighboring cells and anatomically connected brain regions, selectively affecting vulnerable cells, including glial cells [48–50]. Our findings confirm that pathological α-syn can propagate along dopaminergic pathways and even cross hemispheres. We first evaluated the impacts of O-GlcNAc cycling on α-syn aggregation and interregional transmission in AAV/PFFs mice treated with OSMI-1 or TMG. Brain sections were immunostained with anti-pS129 α-syn antibodies to detect pathological α-syn in the striatum, cortex, and amygdala, regions anatomically connected to the substantia nigra [51]. In AAV/PFFs mice, pS129 α-syn was abundantly deposited within TH^+^ neurons in the substantia nigra (Fig. 5A, B), and also detected in neurons of the ipsilateral striatum (Fig. 5A, C), amygdala (Fig. 5A, D), and cortex (Fig. 5A, E). Furthermore, contralateral brain regions, including the amygdala, cortex, and hippocampus, exhibited pS129 α-syn signals, suggesting interhemispheric propagation (Fig S7A-C). OSMI-1 treatment exacerbated α-syn accumulation in dopaminergic neurons within the substantia nigra and its distribution to connected regions, whereas TMG treatment mitigated these effects (Fig. 5A–E; Fig S7A-C).

Clinical and experimental evidence suggests a strong relationship between microglial activation, neuroinflammation, and the degeneration of dopaminergic neurons in PD [52]. Microglia internalize neuronally secreted α-syn, triggering intracellular activation of the NLRP3 inflammasome, an endogenous pathway driving neuroinflammation [53]. To investigate whether modulating O-GlcNAcylation affects α-syn release and subsequent microglial activation, we stained microglia in the substantia nigra and striatum of AAV/PFFs mice treated with OSMI-1 or TMG. Aggregated α-syn was detected within microglia labeled with the Iba1 antibody in the ipsilateral striatum of AAV/PFFs mice (Fig. 5F–H). Given that OSMI-1 or TMG treatments altered α-syn particle deposition in these brain regions (Fig. 5A), we speculated that O-GlcNAcylation may regulate microglial internalization of α-syn and subsequent inflammatory activation. Quantification of Iba1^+^ microglia supported this hypothesis, which revealed a significant increase in activated microglia within the substantia nigra and striatum following OSMI-1 treatment, while TMG administration reduced microglial activation (Fig. 5I–K).

To explore how O-GlcNAc modification influences microglial inflammatory activation, potentially driven by α-syn secretion from neuronal cells, we conducted in vitro assays. Conditioned medium (CM) from SH-SY5Y cells pretreated with conformational α-syn strains was applied to BV-2 cells and cultured for 24 hours. Subsequently, the levels of cellular O-GlcNAcylation were assessed. BV-2 cells exposed to CM demonstrated reduced levels of OGT and total O-GlcNAcylated proteins, with oligomer and PFFs treatments exhibiting the most pronounced effects (Fig. 5L, M). To determine whether the observed downregulation of O-GlcNAcylation and upregulation of NLRP3 levels in recipient BV-2 cells were specifically attributable to α-syn secretion, we employed a precipitation method to deplete α-syn from the CM. The CM was treated with a pan anti-α-syn antibody (MJFR1) conjugated to magnetic beads and then applied to BV-2 cells. CM derived from SH-SY5Y cells treated with conformational α-syn strains efficiently downregulated O-GlcNAcylation and OGT levels, but not OGA, in BV-2 cells (Fig S8A-D). The CM with the PFFs treatment has the most efficient effects. Importantly, α-syn depletion from the CM did not alter the ability of the CM to downregulate O-GlcNAcylation or OGT levels in BV-2 cells (Fig S8A-D). These results suggest that the reduction in O-GlcNAcylation observed in BV-2 cells is not directly related to changes in OGT or OGA levels or to the uptake of α-syn itself. This conclusion was further supported by direct treatment of BV-2 cells with conformational α-syn strains, OSMI-1, or TMG in PFF-pretreated cells (Fig S8F-I and J).

The CM derived from SH-SY5Y cells treated with PFFs induced upregulation of NLRP3 in BV-2 cells, although this effect was diminished after α-syn deletion (Fig S8A and E). Furthermore, the increase in NLRP3 levels in BV-2 cells was found to depend on the dose of PFFs used to treat the donor SH-SY5Y cells (Fig S8K, L). Neuronal cells seeded with PFFs effectively triggered NLRP3 upregulation in recipient BV-2 cells, an effect that was enhanced by OSMI-1 treatment but mitigated by TMG administration (Fig. 5N, O). These findings were further corroborated by transiently silencing OGT using siRNA in SH-SY5Y cells seeded with PFFs, which similarly resulted in upregulated NLRP3 levels in BV-2 cells (Fig S9A, B). To better characterize the state of secreted α-syn, proteins were precipitated from the CM using the methanol/chloroform method and analyzed by immunoblotting with total and pS129 α-syn antibodies. Notably, pS129 α-syn in the high molecular weight range (~35 kDa) was elevated under OSMI-1 treatment. Conversely, under TMG treatment, the levels of pan α-syn, but not pS129 α-syn, in the high molecular weight range were relatively increased (Fig S9C, D). The reduced phosphorylation of α-syn in the TMG treatment group suggest that O-GlcNAc modification primarily occurs in secreted α-syn.

Microglial internalization of α-syn has been linked to the maturation and release of pro-inflammatory cytokines [9]. To investigate the impact of O-GlcNAcylation on inflammatory responses, we assessed the transcription and secretion of IL-1β, IL-6, IL-18, and TNF-α in co-cultured BV-2 cells and mouse brain tissue using RT-qPCR and enzyme-linked immunosorbent assay (ELISA) (Fig S7D and S9E). Neuronal cells treated with PFFs significantly increased the secretion of IL-1β, IL-6, IL-18, and TNF-α in co-cultured BV-2 cells. Although OSMI-1 treatment did not further enhance these PFF-induced effects, TMG treatment effectively reduced the levels of IL-1β, IL-6, IL-18, and TNF-α in the co-culture medium (Fig S9E-I). Similar trends were observed in vivo, as the mRNA and protein levels of these inflammatory cytokines in AAV/PFFs-treated mice were similar to the in vitro result (Fig S7D-L). These findings suggest that O-GlcNAcylation modulates pathological α-syn-induced pro-inflammatory responses in microglia influencing events from cytokine transcription to secretion.

### Blockade of autophagosome-lysosome pathway suppresses O-GlcNAcylation-mediated pathological α-syn degradation and microglial activation

**Fig. 6:**
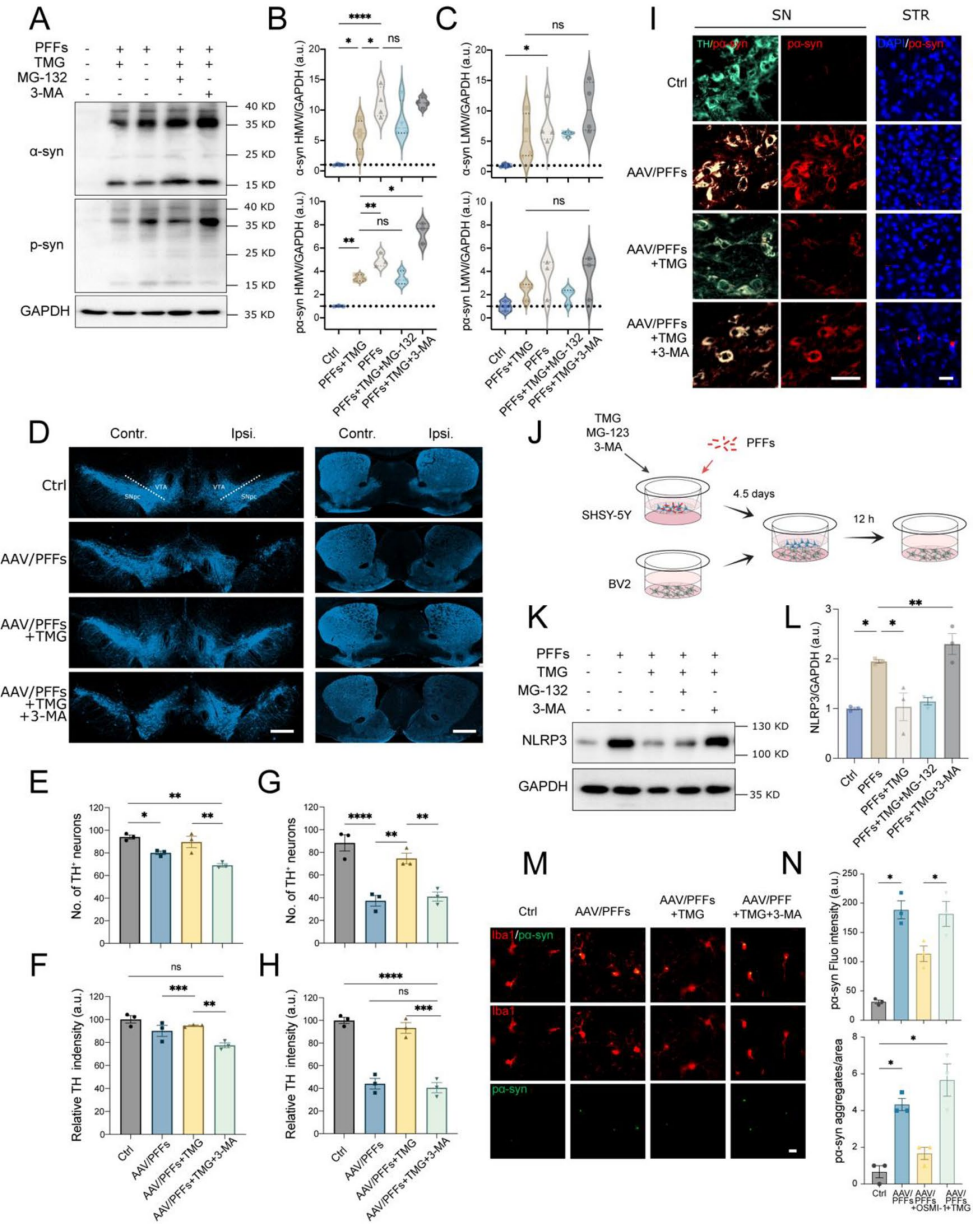
Blocking of autophagosome-lysosome flux re-aggravates the O-GlcNAc-elicited amelioration of α-syn pathology. (**A**) immunoblot analysis for pα-syn in PFFs-seeded SH-SY5Y cells treated with TMG, MG-132, and 3-MA. (**B**-**C**) quantification of the levels of the high molecular weight bands (**B**) and lower molecular weight bands (**C**) of total α-syn (upper) and pS129 α-syn (lower), normalized to GAPDH (*n* = 3–4). (**D**) fluorescent visualization of dopaminergic staining (TH^+^) in the substantia nigra and striatum of AAV/PFFs mice with OSMI-1 or TMG treatment. Scale bar = 500 μm. (**E**-**H**) summary statistics for TH^+^ cell number in the contralateral side (**E**) and ipsilateral side (**G**) of the substantia nigra and TH fiber intensity in the contralateral side (**F**) and ipsilateral side (**H**) of the striatum (*n* = 3). (**I**) Representative immunostaining of pα-syn (red) localized to the dopaminergic neurons (TH^+^, green) in the substantia nigra and pα-syn distribution in striatum of AAV/PFFs mouse brains. Scale bars = 50 μm (left) and 25 μm (right). (**J**) PFFs-seeded SH-SY5Y cells were treated with TMG plus MG-132 or 3-MA for 4.5 days and co-cultured with BV-2 cells for 12 h. (**K**) immunoblot analysis of NLRP3 levels in BV-2 cells. (**L**) quantification of NLRP3 levels normalized to GAPDH (*n* = 3). (**M**-**N**) immunostaining and quantification of pα-syn (green) in microglia (Iba1^+^, red) within the striatum of AAV/PFFs-injected mice with or without combined compound treatments (*n* = 3). Scale bar = 10 μm. Data are presented as means ± SEM. *p < 0.05, **p < 0.01, *p < 0.001 and **p < 0.0001 by one-way ANOVA with Tukey’s post-hoc test

Two major proteolytic pathways, the ubiquitin-proteasome system and the autophagy-lysosomal pathway, are critical for degrading misfolded α-syn, thereby preventing its pathological accumulation [54–56]. We next examine whether enhancing cellular O-GlcNAcylation significantly reduces α-syn accumulation via proteolytic pathways. To address this, we treated SH-SY5Y cells seeded with PFFs using TMG in combination with MG-132 (a proteasome inhibitor) or 3-MA (an autolysosome inhibitor). The results showed that MG-132 did not block TMGs ability to reduce phosphorylated α-syn levels in SH-SY5Y cells (Fig. 6A). In contrast, 3-MA strongly inhibited the effect of TMG, as evidenced by the accumulation of total and phosphorylated α-syn in the high molecular weight fraction (Fig. 6A–C). Importantly, the neuroprotective effect of TMG, which mitigates TH-positive neuron loss, was reversed in AAV/PFFs mice treated with 3-MA (Fig. 6D–G). This reversal was further supported by decreased TH staining intensity in the ipsilateral striatum of AAV/PFFs mice under TMG and 3-MA co-treatment (Fig. 6D, F, and H). Consistently, phosphorylated α-syn levels in the substantia nigra and striatum were significantly increased following TMG/3-MA treatment in AAV/PFFs mice (Fig. 6I). Next, we evaluated the effect of the two proteolytic pathways on the downregulation of NLRP3 levels in BV-2 cells induced by α-syn secretions following O-GlcNAcylation modification in donor neurons. The donated SH-SY5Y cells seeded with PFFs were treated with TMG combined with 3-MA or MG-132 and then co-cultured with BV-2 cells in trans-well chambers (Fig. 6J). Treatment with 3-MA, but not MG-132, significantly inhibited the effect of TMG on donor SH-SY5Y cells, as evidenced by the re-upregulation of NLRP3 levels in co-cultured BV-2 cells (Fig. 6K, and L). Similarly, 3-MA treatment prevented TMG-mediated reductions in aggregated α-syn accumulation in activated microglia of AAV/PFFs mice (Fig. 6M, N). Additionally, Iba1 staining intensity in microglia of the substantia nigra and striatum increased following 3-MA treatment, further supporting the involvement of the autophagy-lysosome pathway in O-GlcNAcylation-mediated α-syn degradation (Fig S10A-C). These findings were corroborated by using another autophagosome-lysosome inhibitor, chloroquine (CQ), in the trans-well co-culture system (Fig S10D). CQ inhibited the ability of TMG to reduce α-syn aggregation in donor cells and to downregulate NLRP3 levels in BV-2 cells (Fig S10E, F). Furthermore, we assessed whether PFF-induced aggregation impacted the proteolytic degradation of OGT and OGA proteins under whole-cell O-GlcNAcylation modification. Treatments with 3-MA, CQ, or MG-132 did not alter OGT or OGA protein levels (Fig S11A-B), indicating that changes in these enzymes’ cellular levels are unrelated to proteolytic degradation.

### Regulation of O-GlcNAcylation is associated with polyubiquitinated α-syn inclusion formation and lysosome degradation

**Fig. 7:**
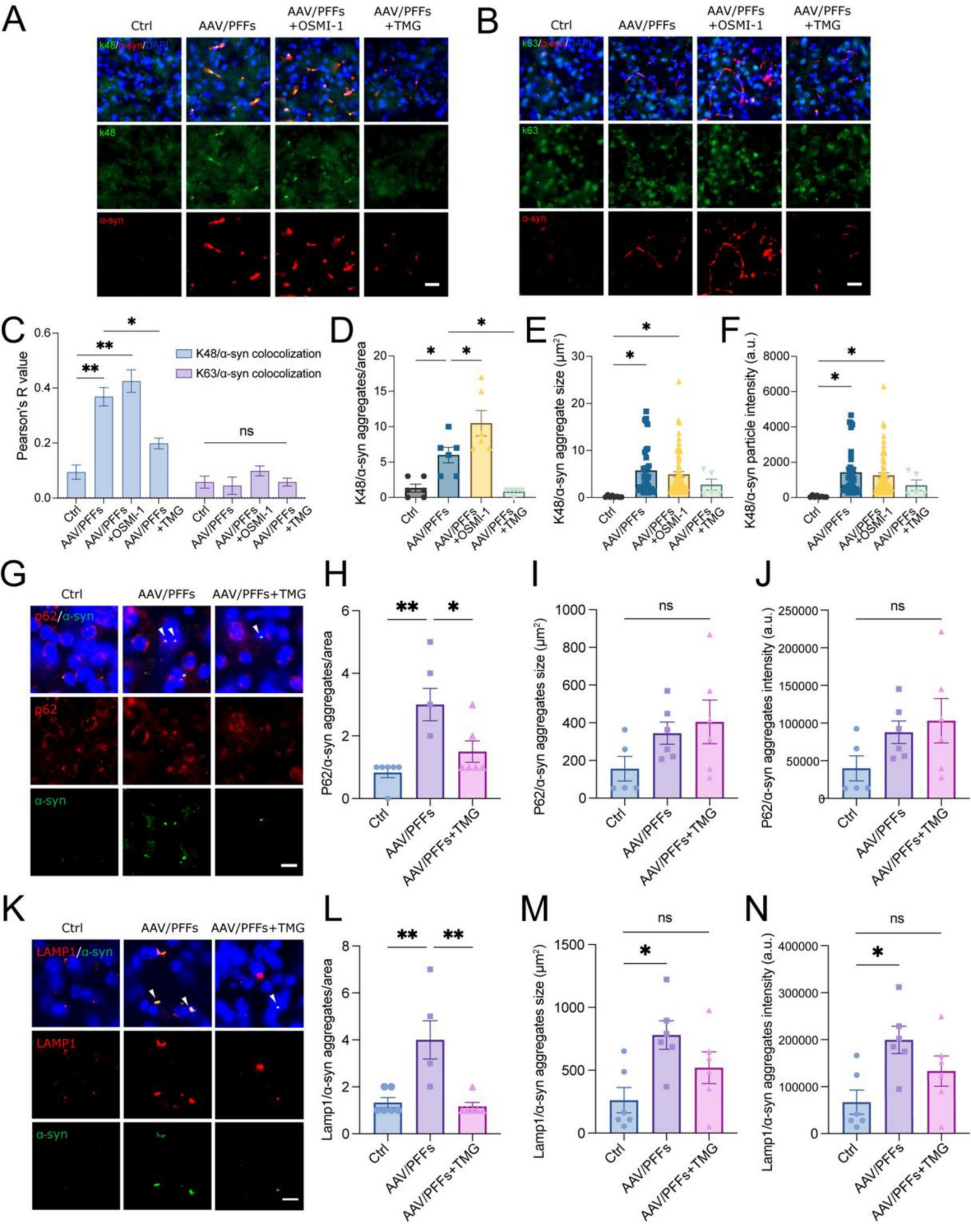
Modulating O-GlcNAc is critical for proteolysis of aggregated α-syn. (**A**-**B**) immunohistochemistry of α-syn (red) and polyubiquitin K48 (**A**) or K63 (**B**) (green) in the striatum of AAV/PFFs mice treated with OSMI-1 and TMG. Scale bar = 25 μm. (**C**) Pearson correlation analysis between α-syn and pU K48 or K63 in the images shown in panels A and B (*n* = 3). (**D**-**F**) quantification of the parameters related to pU K48/α-syn aggregates: number of aggregates per area (**D**), aggregate size (**E**), and particle intensity (**F**). (**G**) Representative p62 (red) and α-syn (green) immunofluorescence images of the striatum of AAV/PFFs mice treated with or without TMG, using anti-p62 antibody and α/β-syn (syn211) antibodies. (**H**-**J**) quantification of p62 and α-syn co-localization for parameters including aggregate number per area (**H**), aggregate size (**I**), and intensity (**J**). (**K**) Representative immunofluorescence images of the lysosome marker (LAMP1^+^, red) and α-syn (green) in the stratum of AAV/PFFs mice treated with or without TMG. (**L**-**N**) analysis of LAMP1 and α-syn co-localization in terms of aggregate number per area (**L**), aggregate size (**M**), and intensity (**N**). Data are presented as means ± SEM. *p < 0.05 and **p < 0.01 by 1-way ANOVA with Tukey’s post-hoc test

The degradation of aggregated proteins relies on autophagy cargo receptors, such as p62/SQSTM1, which recognize ubiquitin-tagged substrate proteins and interact with autophagosome-associated proteins. This process facilitates autophagosome biogenesis and subsequent fusion with lysosomes for substrate degradation [57, 58]. Our mass spectrometry analysis revealed that dimeric α-syn is subject to ubiquitination (Table S1), suggesting a critical role for ubiquitination in α-syn inclusion formation and its proteolytic degradation. To investigate how modulating O-GlcNAcylation affects proteolysis associated with K48- or K63-linked ubiquitin, we performed double-staining of polyubiquitin K48 or K63 with α-syn in the brains of OSMI-1- and TMG-treated AAV/PFFs mice. IHC staining showed that K48-linked ubiquitin, but not K63-linked ubiquitin, colocalized with α-syn aggregates in the AAV/PFFs mouse brain (Fig. 7A–C). OSMI-1 treatment increased the number of K48-linked ubiquitinated inclusions without altering their size or intensity (Fig. 7A, B, D-F). In contrast, TMG treatment significantly reduced K48 colocalization with α-syn aggregates (Fig. 7A, C). These findings suggest that O-GlcNAcylation modulation impacts ubiquitination patterns associated with α-syn inclusions.

We next assessed whether upregulating O-GlcNAcylation promotes the degradation of α-syn aggregates via the autophagy cargo linkage necessary for autophagosome-lysosome fusion. Previous studies have reported that PFF-induced α-syn aggregates transiently block autolysosome degradation, as evidenced by the colocalization of α-syn inclusions with autophagy cargo receptors or lysosome markers [59]. To evaluate the effects of TMG treatment, we examined the colocalization of the autophagy cargo receptor p62 and the lysosome-associated membrane protein 1 (LAMP1) with α-syn aggregates in AAV/PFFs mouse brains. While both p62 and LAMP1 colocalized with α-syn aggregates, TMG treatment significantly reduced their colocalization (Fig. 7G, K). In untreated AAV/PFFs mice, the number, size, and intensity of LAMP1/α-syn colocalized aggregates were markedly increased (Fig. 7L–N). However, TMG treatment decreased the recruitment of both p62 and LAMP1 to α-syn aggregates. Correspondingly, the number, size, and intensity of double-positive signals were significantly reduced, particularly for LAMP1/α-syn colocalization (Fig. 7G–J and L-N). Together, these findings support a crucial role for O-GlcNAcylation in promoting the degradation of α-syn aggregates via the autophagosome-lysosome pathway.

## Discussion

In this study, we explored how regulating O-GlcNAcylation through inhibition of OGT or suppression of OGA influences α-syn aggregation, propagation, dopaminergic neuron loss, and neuroinflammation in cellular and animal models (Fig S12). Our findings revealed a direct association between O-GlcNAcylation and α-syn, with changes in O-GlcNAcylation levels affecting the recruitment of α-syn into high-molecular-weight assemblies during fibril-seeding aggregation. Additionally, fibrillar α-syn reduces global O-GlcNAcylation levels and downregulates OGT expression in both neuronal and microglial cells, representing an aspect of α-syn toxicity. Pharmacological inhibition of autolysosome flux exacerbated α-syn pathology, implicating O-GlcNAcylation as a critical regulator of α-syn degradation through the autolysosomal pathway.

Previous research has indicated that cells coordinate OGT and OGA expression to maintain O-GlcNAcylation homeostasis. O-GlcNAc modification has been shown to reduce α-syn aggregation in vitro and to mitigate dopaminergic neurons degeneration caused by the A53T α-syn mutant [13, 27, 28]. Our data suggest a complex molecular relationship between O-GlcNAcylation cycling and α-syn pathology. Notably, pathological α-syn decreases O-GlcNAcylation in both in vivo and in vitro models. One possibility is that stressors or pathological conditions alter enzyme activity, rather than expression, as evidenced in other neurodegenerative diseases. For example, exposure of neuronal cells to amyloid-beta (Aβ), a hallmark protein of Alzheimer’s disease, leads to reduced O-GlcNAcylation due to S-nitrosylation-induced OGT inactivity [60]. In our study, α-syn fibril seeding decreases cellular O-GlcNAcylation and OGT levels in neuronal cells and mouse brains. In another report, 6-OHDA administration decreased O-GlcNAcylation by increasing OGA, but no changes in OGT levels were observed in vitro. However, in mouse brains, 6-OHDA administration unexpectedly increased both OGT and OGA levels [33]. These observations suggest that O-GlcNAcylation homeostasis may be influenced not only by the expression of opposing enzyme but also by their activity states and potentially other modulatory factors. It is important to note that the response of O-GlcNAcylation to α-syn pathology varies across brain regions. In mice injected with PFFs, O-GlcNAcylation was reduced in the substantia nigra and hippocampus but elevated in the cortex, despite no changes in OGT or OGA expression. This aligns with previous reports showing increased O-GlcNAcylation levels in the temporal cortex of PD patients [30]. These discrepancies suggest that regional differences in O-GlcNAcylation, such as increased modifications in the cortex but reduced levels in the substantia nigra, may reflect variations in cellular metabolic states or enzyme activity. For instance, in the 3xTg-AD mouse model, decreased O-GlcNAcylation was associated with reduced OGA activity, despite no changes in OGT or OGA levels [61]. These findings emphasize the complexity of O-GlcNAcylation regulation in amyloid protein pathology. Further investigations are needed to elucidate the precise mechanisms governing O-GlcNAcylation and its role in neurodegenerative diseases.

The accumulation and intercellular spreading of α-syn significantly exacerbate the progression of PD-related phenotypes [62]. Seeding PFFs to induce α-syn aggregation in wild-type animals is a useful model that mimics the progressive transmission of α-syn aggregates and dopaminergic neuronal cytotoxicity [63, 64]. Consistent with the previous report [51], bilateral injections of PFFs into the striatum led to the formation of α-syn inclusions in the anatomically connected brain regions such as cortex, amygdala, and hippocampus. We observed a region-specific reduction in O-GlcNAcylation levels concomitant with increased α-syn aggregation. Importantly, treatment with OSMI-1 exacerbated the spread of α-syn from the substantia nigra to connected regions, including the contralateral hemisphere, while TMG treatment reduced α-syn spreading.A recent study demonstrated that elevated O-GlcNAcylation levels are critical for preventing dopaminergic neuron loss in the SNpc caused by overexpression of AAV-α-syn A53T [13]. Inhibition of OGT by OSMI-1 reduces cell viability [43, 44], and conditional downregulation of OGT has been shown to increase the sensitivity of dopaminergic neurons [13]. Consistent with this, our findings revealed that TMG treatment significantly increased the survival of dopaminergic neurons in the substantia nigra of AAV/PFFs-injected mice. Conversely, OSMI-1 treatment reduced cell viability and the number of dopaminergic neurons on both the ipsilateral and contralateral sides caused by α-syn pathology, but not the drug alone. These findings further support the hypothesis that modulating O-GlcNAcylation affects α-syn aggregation and propagation, as well as its protective role against dopaminergic neuron loss associated with α-syn toxicity.

Growing evidence indicates that microglia-driven neuroinflammation is a key contributor to the progression of α-synucleinopathies and dopaminergic neuronal toxicity in PD [65]. Our findings, along with previous studies, indicate that the internalization of extracellular α-syn by microglia is a critical trigger for neuroinflammation [9, 65]. In AAV/PFFs mice, Iba1 immunostaining revealed that O-GlcNAcylation effectively modulates microglial activity induced by the spread of pathogenic α-syn between anatomically connected brain regions. One feature of microglial response to stress is the assembly of the NLRP3 inflammasome, which promotes the cleavage and activation of caspase-1, leading to the release of pro-inflammatory cytokines such as IL-1β and IL-18 [66, 67]. In our study, PFFs treatment could sufficiently activate microglia to elevate NLRP3 levels, cytokine secretion, and reduce O-GlcNAcylation. Treatment with OSMI-1 or TMG significantly impacts the pathogenic α-syn-induced inflammation in both co-culture systems and mouse brains. Interestingly, when α-syn was removed from the culture medium using a precipitation method and then applied to microglial cells, NLRP3 levels were significantly reduced, while O-GlcNAcylation levels remained unchanged. These findings suggest that O-GlcNAcylation could regulate microglial activation by influencing the upregulation of NLRP3 expression by pathogenic α-syn. Given its widespread presence in the brain, O-GlcNAcylation may play a key role in coordinating multiple cell types, including microglia, and in regulating inflammation [68, 69] and α-syn processing, which protects against neuronal toxicity [70, 71].

Previous studies have established that the early transition of α-syn from its soluble monomeric form to disease-associated oligomeric conformations represents a critical step in the cytotoxic aggregation pathway [72]. In our study, PFFs seeding leads to the assembly of higher molecular weight species enriched with dimeric α-syn in neuronal cells and mouse brains. Notably, a substantial proportion of this dimeric α-syn was O-GlcNAcylated. Although OSMI-1 treatment did not produce detectable changes in α-syn dimers, TMG treatment significantly increased O-GlcNAc-modified α-syn, despite an overall reduction in total α-syn levels. In another report, TMG treatment upregulates the accumulation of the native form of α-syn [30]. This suggests that O-GlcNAc modification of α-syn protein may influence the initiation of α-syn aggregation, specifically dimer formation. Several O-GlcNAc-modified residues, including threonine (T) 54, 59, 64, 72, 75, and serine (S) 87, located within the non-amyloid-β component (NAC) domain of endogenous α-syn, have been identified in mice and humans [20, 73–75]. Our findings reveal two novel O-GlcNAcylation sites, serine 42 (S42) and threonine 44 (T44), located in the N-terminal region of dimeric α-syn isolated from fibril-seeded neuronal cells. The novel modification sites were identified through in vitro experiments; however, further studies are needed to determine whether these modifications occur at conserved α-syn sites in the brains of patients and animal models.

The autophagy-lysosomal pathway (ALP) and the ubiquitin-proteasome system (UPS) are critical mechanisms for clearing amyloid aggregates, and their dysfunction has been implicated in α-syn aggregation and intercellular propagation [57, 58]. Ubiquitin chains, such as K48- or K63-linked ubiquitin, mediate substrate protein degradation through the UPS or ALP, respectively. Aggregated α-syn has been shown to significantly impair proteasome-mediated protein degradation [76, 77]. To investigate the role of O-GlcNAcylation in α-syn proteolysis, we used CQ, which blocks autophagosome maturation into autolysosomes [78], and 3-MA, an effective inhibitor of PtdIns3K III [79], both of which target the late-stage autolysosome formation. Our findings showed that inhibiting autophagosome-lysosome fusion with 3-MA or CQ significantly restrained the TMG-induced reduction of α-syn aggregates. In contrast, inhibition of the UPS with MG-132 had no comparable effect. These results indicate that pathological α-syn degradation is likely mediated through the autophagosome-lysosome pathway, regulated by O-GlcNAcylation modifications, particularly in the late stages [23, 26, 30]. Previous studies have reported that increased O-GlcNAcylation of proteins such as SNAP-29 and GRASP55 inhibits autophagosome-lysosome fusion, thereby disrupting late-stage autophagy [23, 80]. Although O-GlcNAcylation influences α-synucleinpathology primarily through later stage of the autophagy-lysosome pathway, (1) it remains possible that O-GlcNAcylation also regulates the initial stage of this pathway [23, 81], and (2) we acknowledge that it may affect α-synuclein pathology via alternative mechanisms, such as modulating cell internalization [38].

In summary, our findings demonstrate that the transmission of pathological α-syn, derived from conformational strain aggregation, between cells and across brain regions leads to a reduction in total O-GlcNAcylation and OGT levels in both cultured cells and animal brains. Pharmacological inhibition of OGA or OGT effectively alleviates or exacerbates α-syn aggregation, spreading, dopaminergic neuron degeneration, and neuroinflammation, which contribute to α-synucleinopathies in neurodegenerative diseases. We also provide evidence that α-syn is directly O-GlcNAcylated, and that modulation of O-GlcNAcylation can influence fibril-driven dimer formation, an early step in the aggregation process. Furthermore, upregulation of O-GlcNAcylation is mechanistically linked to the autophagosome-lysosome pathway, facilitating the degradation of pathological α-syn. These results highlight the significance of post-translational modifications in α-syn pathogenesis and offer novel insights into the therapeutic potential of targeting O-GlcNAcylation for neurodegenerative diseases such as PD.

## Materials and methods

### Preparation of conformational α-syn strains

Recombinant full-length human α-syn was purified as described previously [82]. Human wild-type α-syn was expressed in BL21 (DE3) E. coli cells and resuspended in a buffer containing 50 mM Tris-HCl (pH 7.4), 1 mM EGTA, and 1 mM DTT with added protease inhibitors before use. The recombinant protein was purified using a pre-equilibrated Capto Q Sepharose column (GE Healthcare Life Sciences). The α-syn proteins were eluted with an elution buffer (50 mM Tris-HCl, pH 7.4, 1 mM EGTA, 1 mM DTT, 0.2 M NaCl). Ammonium sulfate was then added to achieve 50% saturation, followed by centrifugation. The resulting pellet was resuspended in 1 mL of 30 mM Tris-HCl (pH 7.5) and dialyzed overnight against the same buffer. After dialysis, the solution was centrifuged at 100,000 × g for 20 minutes at 4 °C, and the supernatant was collected as the purified α-syn monomer.

For fibril preparation, 5 mg/ml of α-syn monomer was continuously rotated in a shaker at 1000 rpm for 7 days at 37 °C [83]. The resulting fibrils were briefly sonicated (100 s, 2 s on/off cycles) at 10% amplitude and characterized using transmission electron microscopy (TEM) [84]. Validation of fibrillar α-syn was performed via 12% SDS-PAGE and immunoblotting with anti-α-syn antibodies. For additional characterization, 20 μL of PFFs was transferred into a polyallomer tube, ultracentrifuged at 100,000 × g for 60 minutes, and the supernatant was transferred to a new tube. The pellet was resuspended in 20 μL of PBS. Both the supernatant and pellet fractions were analyzed via SDS-PAGE and visualized with Coomassie brilliant blue staining [83]. For oligomer preparation [17], 12 mg/mL of α-syn monomer was incubated in 30 mM Tris-HCl (pH 7.4) at 37 °C for 20 h without agitation. The fibril was removed by ultracentrifugation at 32,400 rpm for 2 h, and the protein concentration was determined using a BCA assay.

### TEM analysis

For negative-staining TEM, 10 μL of α-syn PFFs were placed on 300 mesh Formvar/carbon film-coated copper grids (EMS supply, USA) and incubated for 3 minutes. The grids were then washed twice with double-distilled water (ddH2O) for 1 min each, followed by staining with 2% uranyl acetate for 1 min. The grids were subsequently examined using a JEOL 1010 transmission electron microscope.

### Dot blot analysis

Dot blot analysis of conformational strains of α-syn was performed by spotting 2.0 μL (0.5 mg/ml) of each sample onto a 0.45 μm polyvinylidene fluoride (PVDF) membrane (Millipore). The membrane was blocked with 5% bovine serum albumin (BSA) in Tris-buffered saline containing 0.1% Tween-20 (TBST) for 1 h at room temperature. Following blocking, the membrane was incubated with A11 polyclonal antibodies and α-syn antibody (MJFR1) for 3 h at room temperature. After incubation, the membrane was treated with a horseradish peroxidase (HRP)-conjugated anti-rabbit secondary antibody for 1 h at room temperature. Immunolabeled signals were visualized using the Bio-Rad enhanced chemiluminescence (ECL) detection system.

### HPLC-SEC testing

Size-exclusion chromatography (SEC) was performed using a Shimadzu LC-20AD high-performance liquid chromatography (HPLC) system (Shimadzu, Japan) equipped with a refractive index detector (RID-20A, Shimadzu, Japan). Chromatographic separation was carried out using TSKgel G3000SWXL gel columns (TOSOH, Japan) coupled with a KW-G 6B guard column. The eluent used was 0.1 M sodium sulfate (pH 6.7), with a flow rate of 0.5 mL/min at 30 °C. Dual-wavelength detection was employed for enhanced characterization, with the higher wavelength (280 nm) used for a broader linear detection range and the lower wavelength (220 nm) providing increased sensitivity for low-abundance species. Each sample, prepared at a concentration of 10 mg/mL, was injected at a volume of 25 μL.

### Cell culture, drug treatment and cell viability assay

SH-SY5Y and BV-2 cells were cultured in DMEM supplemented with 10% fetal bovine serum (FBS) and 1% penicillin/streptomycin at 37 °C in a humidified incubator containing 5% CO2. Confluent cultures were washed with phosphate-buffered saline (PBS), detached using 0.25% trypsin-EDTA solution, and reseeded into six-well plates for overnight incubation. On the next day, SH-SY5Y cells were treated with different forms of α-syn (40 μg/mL) or transfected with a plasmid encoding α-syn using the jetPRIME® transfection reagent according to the manufacturer’s protocol. Cells were harvested 36–72 h post-transfection. SH-SY5Y cells treated with conformational strains of α-syn were maintained for 5 days. For experiments involving PFFs, SH-SY5Y cells were exposed to PFFs for 24 hours, after which the medium was removed and the cells were rinsed with pre-warmed PBS. Fresh medium containing either 20 μM OSMI-1 (TargetMol, T16409) or 1 μM TMG (TargetMol, T6056) was then added, and the cells were cultured for an additional 4 days. Cell viability was subsequently evaluated using the CCK-8 assay (MCE, HY-K0301) according to the manufacturer’s instructions. Additionally, SH-SY5Y cells seeded with PFFs were pre-treated with 10 μM MG-132 (TargetMol, T2154), 2 mM 3-methyladenine (3-MA, TargetMol, T1879), or 10 μM chloroquine (CQ, TargetMol, T8689) for 1 h before TMG treatment.

### Gel electrophoresis and HPLC-MS/MS

Whole cell lysates (200 μg) were fractionated through SDS-PAGE electrophoresis. Proteins on the gel were visualized by staining with Coomassie Brilliant Blue G-250. Gel bands corresponding to the relative molecular weight of ~ 35 kDa, indicative of α-syn dimers, were excised. The gel slices underwent decolorization and enzymatic digestion before desalting on a C18 column, followed by vacuum freeze-drying. The resulting lyophilized powder was reconstituted in 10 μL of Solution A (ddH2O containing 0.1% formic acid) and centrifuged at 14,000 g for 20 minutes. Supernatants (400 ng) were analyzed using the L-3000 HPLC system (RIGOL TECHNOLOGIES Co., LTD.) with a separation flow rate of 300 nL/min. The samples were introduced into an Orbitrap Eclipse Tribrid Mass Spectrometer (Thermo Fisher Scientific Co., LTD.) equipped with a Nanospray Flex™ (NSI) ion source. The first-level mass spectrometry was performed in the mass range of 350–1500 m/z, with a resolution of 12,000 and a maximum injection time of 50 ms in the C-trap. The ion spray voltage was set to 2.0 kV, and the ion transfer tube temperature was maintained at 320 °C. Secondary mass spectrometry was conducted in “Top Speed” mode with a resolution of 15,000 (200 m/z), a maximum injection time of 22 ms, and an automatic gain control (AGC) target of 5 × 104. MS/MS spectra of modified residues were identified using Proteome Discoverer 2.4 software, with searches conducted against mammalian protein databases from UniProt and NCBI.

### Trans well cell co-culture

SH-SY5Y cells were seeded onto the insert membrane of a trans well chamber, while BV-2 cells were cultured in separate 12-well plates. SH-SY5Y cells were treated with PFFs and OSMI-1 or TMG for 4.5 days. After incubation, the insert membrane containing the treated SH-SY5Y cells was placed above the wells containing BV-2 cells in the 12-well plates. Following a 12-hour co-culture, BV-2 cells were harvested, and the lysates were subjected to immunoblot analysis.

### Immunoblot

Brain tissues and cultured cells were homogenized in RIPA lysis buffer, and total protein concentration was quantified using the BCA protein assay kit (Fude Biotech., FD2001). Protein samples (20–60 μg per lane) were resolved on 8–12% sodium dodecyl sulfate-polyacrylamide gel electrophoresis (SDS-PAGE) and transferred onto PVDF membranes (Millipore, IPVH00010) via electrophoresis. Membranes were blocked with 5% nonfat dry milk in Tris-buffered saline (20 mM Tris-HCl, 500 mM NaCl, pH 7.4) containing 0.1% Tween 20 (TBST) for 1 h at room temperature. Blocked membranes were incubated overnight at 4 °C with primary antibodies (Table S2), followed by incubation with horseradish peroxidase (HRP)-conjugated goat anti-mouse or anti-rabbit IgG secondary antibodies. Immunoreactive bands were visualized using an ECL detection system (BIO-RAD). Band intensity was quantified using ImageJ software (NIH), and protein expression levels were normalized to GAPDH or actin as internal controls.

### ELISA

The levels of cytokines, including IL-1β, TNF-α, IL-6, and IL-18, were measured in mouse brain tissue samples and cultured BV-2 cell supernatants collected at the specified time points following drug administration. Cytokine concentrations were analyzed using ELISA kits (ABclonal Bio) according to the manufacturer’s protocol. Briefly, samples were added to pre-coated ELISA plates and incubated with a detection antibody conjugated to HRP. After washing to remove unbound components, the HRP substrate was added, and the reaction was stopped as per the protocol. Absorbance values were measured using a microplate reader (MR-96TC, Gallop) at the appropriate wavelength. Cytokine concentrations were calculated by comparing sample absorbance values to a standard curve generated with the kit’s reference standards.

### Immunoprecipitation

Cells were lysed on ice for 30 minutes in lysis buffer containing 50 mM Tris-HCl (pH 7.4), 150 mM NaCl, 1 mM EDTA, 1% Triton X-100, and protease inhibitors. The lysates were centrifuged at 13,000 rpm for 10 min at 4 °C, and the supernatants were collected. Total protein concentrations were quantified and normalized using the BCA assay. For immunoprecipitation, 600 μg of protein extract was incubated with 6 μg of anti-O-linked N-acetylglucosamine (O-GlcNAc) antibody (Invitrogen, MA1-072) overnight at 4 °C with gentle rotation. The following day, 50 μL of pre-washed protein A/G beads (Selleck, B23202) were added to the reaction mixture and incubated for 2 h at 4 °C. Beads were washed thoroughly with lysis buffer to remove unbound proteins. Bound proteins were eluted by adding 50 μL of 1× SDS loading buffer, then incubating at 95 °C for 5 min. The eluted proteins were then analyzed via immunoblotting using the appropriate primary antibodies to assess specific targets.

### Immunohistochemistry/immunocytochemistry

Mice were anesthetized and transcardially perfused with 0.9% saline, followed by 4% paraformaldehyde (PFA) in PBS. The brains were extracted, post-fixed in 4% PFA overnight, and subsequently immersed in 20 and 30% sucrose solutions for cryoprotection. Coronal brain sections (30 μm thickness) were prepared using a cryostat microtome. The tissue sections were washed with PBS, post-fixed in 4% PFA for 20 min, and permeabilized with 0.3% Triton X-100. The sections were then incubated overnight at 4 °C with primary antibodies diluted in 1% BSA in 0.3% Triton X-100 blocking buffer. Following three washes with PBS, the sections were incubated with fluorescently labeled secondary antibodies for 1 h at room temperature. Visualization and imaging were performed using a confocal laser scanning microscope (Carl Zeiss) and a digital slide scanner (Olympus). Images were analyzed using ImageJ/FIJI software. For cell immunofluorescence, cells grown on coverslips in 24-well plates were fixed with 4% PFA at room temperature for 20 min. Subsequent staining procedures were similar to the immunohistochemical protocol described.

### RNA extraction and RT-qPCR

For RT-qPCR analysis, mouse brains were dissected and homogenized in 1 mL of Trizol reagent (Thermo Fisher Scientific, USA). Total RNA was extracted following the manufacturer’s instructions, and RNA concentrations were determined using a spectrophotometer. A total of 1 μg of RNA was reverse-transcribed into cDNA using HiScript III RT SuperMix for qPCR (Vazyme, R323) according to the manufacturer’s protocol. qPCR was performed with specific primers for each target gene (Table S3) and analyzed using the comparative cycle threshold (Ct) method 2−*ΔΔ*CT.

### Triton-X-100-soluble and -insoluble fractionation

Cells were homogenized in Triton X-100 soluble buffer containing 1% Triton X-100, 0.5 μM EDTA, 10 mM Tris-HCl (pH 7.4), 150 mM NaCl, protease inhibitors, and phosphatase inhibitor cocktails, as previously described [70]. The homogenate was centrifuged at 13,000 rpm for 15 min at 4 °C. The resulting supernatant, representing the detergent-soluble fraction, was collected. The pellet was then resuspended in detergent-insoluble buffer containing 50 mM Tris-HCl (pH 8.0), 1% Triton X-100, 2% SDS, 1% sodium deoxycholate, 1% NP-40, 0.5 μM EDTA, and protease and phosphatase inhibitor cocktails. This lysate was centrifuged again at 13,000 rpm for 15 min at 4 °C, and the supernatant was collected as the Triton X-100 insoluble fraction.

### Purification of cell culture supernatant proteins

The purification of proteins from cell culture supernatants was conducted as previously described [85]. Briefly, the cell culture supernatant was collected and centrifuged to remove dead cells and debris. A 500 μL aliquot of the clarified supernatant was transferred to a clean tube, followed by the addition of 500 μL methanol and 125 μL chloroform. The mixture was vortexed thoroughly and centrifuged at 13,000 rpm for 5 min to precipitate the proteins. The upper aqueous phase was carefully discarded, avoiding disturbance of the protein layer. An additional 500 μL of methanol was added for washing, and the mixture was centrifuged again at 13,000 g for 5 min. The supernatant was removed, and the protein pellet was air-dried at 37 °C for 5 min. Finally, the pellet was resuspended in 30 μL of 1× SDS loading buffer and vortexed. The samples were boiled and subsequently loaded onto 12–15% SDS-PAGE gels for immunoblotting analysis.

### Molecular cloning and pAAV-hsyn-mSNCA-eGFP-WPRE preparation

The mouse α-synuclein (*mSNCA*, 20,617) gene was first amplified by polymerase chain reaction (PCR). Subsequently, the amplified product, measuring 434 bp, was digested with ApaI and XhoI. The resulting fragment was inserted into the backbone of the pAAV-hsyn-*eGFP*-WPRE vector (Addgene item no. #50465). Following gel extraction of the digested fragments, the assembly of the pAAV-hsyn-*mSNCA*-*eGFP*-WPRE construct was performed using a Gibson Assembly Reaction kit (NEB, New England Biolabs).

### Animal care, surgeries, and microinjection

All animal care and experimental procedures adhered to the National Institutes of Health Guide for the Care and Use of Laboratory Animals and were approved by the Institutional Animal Care and Use Committee of Soochow University (Approval No. 202210A0007). C57BL/6 mice used in this study were maintained in a pathogen-free facility under a 12-hour light/dark cycle at 25 °C. Surgeries were conducted on C57BL/6J mice aged 2–4 months using a digital stereotaxic frame (David Kopf Instruments). Anesthesia was induced and maintained with 1.25% tribromoethanol. Proteins for injection were drawn into a gas-tight syringe equipped with a 26-gauge needle (Hamilton) and delivered with precision using a digital pump. For bilateral striatum injections, solutions containing 10 μg of proteins were administered using the following stereotaxic coordinates: 0.2 mm anterior, 2.0 mm lateral to the bregma, and 2.6 mm ventral relative to the skull surface [62]. For AAV/PFFs injections, the protocol was modified as previously described [86]. A total of 2.0 × 10^9^ viral particles of AAV vector mixed with 10 μg of PFFs was injected into the right substantia nigra. The injection coordinates were: A/P −3.0 mm (from bregma), M/L −1.25 mm, and D/V −4.5 mm (from the dura). Four weeks after the injection, mice were intraperitoneally treated with 20 mg/kg TMG, 0.4 mg/kg OSMI-1, or 10 mg/kg 3-MA (administered 12 hours prior to TMG). This treatment regimen was maintained for an additional 4 weeks.

### Behavioral assays

#### Open field test

Exploratory activity and anxiety-like behavior were assessed using an open-field apparatus (40 cm × 40 cm × 40 cm, SoftMaze, China). Mice were placed in the center zone of the apparatus, which was defined as a square with 10 cm sides. The time spent in the center zone was recorded and monitored for 10 minutes using a video-imaging system to quantify both exploratory behavior and anxiety-like responses.

#### Rotarod test

The Rotarod test was employed to evaluate motor coordination and balance in mice. Prior to the test, mice were pre-trained on the apparatus (SoftMaze, China) for 5 min daily for two consecutive days, and the fall latency time was recorded as the baseline. During testing, mice were placed on a rotating rod, with the speed gradually increasing from 4 to 40 rpm. Each animal was tested in three separate trials, and the latency to fall was recorded for each. The experiment was terminated if a mouse slipped from the rod or gripped the rod in reverse without rotating in the direction of the rotation.

#### Elevated plus maze test

Anxiety-like behavior was assessed using the elevated plus maze (40 cm × 10 cm × 50 cm, SoftMaze, China). The maze consisted of two open arms and two closed arms, with black walls (20 cm in height) surrounding the closed arms. Each mouse was placed in the center of the maze, facing one of the open arms. The time spent by the animal in the open arms was recorded over a 5-minute period, following a previously established method [87].

#### Balance beam test

Motor coordination and balance were evaluated using a horizontal wooden beam (100 cm long, 1.2 cm diameter) elevated 50 cm above a padded surface [88]. Mice were placed on a platform at one end of the beam, and the time taken to traverse the beam to the opposite side was measured. All mice underwent initial training sessions (3–5 trials) to familiarize them with the task before data collection.

### Transfection of cells with OGT small-interference RNA

OGT small-interference RNA (siRNA) (Santa Cruz, sc-40780) was utilized to knock down OGT expression in SH-SY5Y cells. Transfections were carried out using the jetPRIME® transfection reagent according to the manufacturer’s protocol. After 36 hours, cells were harvested, and whole-cell lysates were prepared for subsequent immunoblotting analysis using OGT siRNAs or control siRNAs.

### Quantification of O-GlcNAcylated α-syn

Tissue and cell samples were homogenized in ice-cold buffer containing 25 mM Tris (pH 7.5), 150 mM NaCl, 1% Triton X-100, 20 μM PUGNAc (Tocris), and an EDTA-free protease inhibitor cocktail, as described previously [46, 89]. After a 30-min incubation on ice, the homogenates were centrifuged at 12,000 rpm for 15 minutes at 4 °C, and the supernatants were collected for chemoenzymatic mass tagging. For each sample, 300 μg of protein was precipitated using methanol/chloroform extraction and then resuspended in 40 μL of 20 mM HEPES (pH 7.9), 1% SDS. Protein concentrations were determined by BCA assay, and the samples were adjusted to a concentration of 2.5 μg/μL protein in 20 mM HEPES (pH 7.9), 1% SDS. Glycoproteins in the samples were labeled with terminal N-acetyl-β-D-glucosamine (GlcNAc) residues using N-azido galactose and a Click-IT kit with the GalT1(Y289L) enzyme (Thermo Fisher Scientific, 33,368) according to the manufacturer’s instructions. Sample reactions without the GalT1(Y289L) enzyme were used as controls. After 20 h of labeling with N-azido galactose, 7.5 μL of freshly made iodoacetamide (600 mM) was added to each sample, followed by a 30-min incubation in the dark with rotation. The samples were then precipitated with methanol/chloroform, and the pellets were solubilized in 100 μL of 10 mM triethanolamine (pH 7.4) (TEA, Sigma-Aldrich), 150 mM NaCl, 1% SDS. To facilitate mass tagging via click chemistry, 10 μL of 10 mM DBCO-mPEG 5 kDa (Tarmet Mol, T17753) was added. After boiling for 5 min at 98 °C, the reactions were precipitated with methanol/chloroform, and the pellets were resuspended in 40 μL of 10 mM TEA (pH 7.4), 150 mM NaCl, 1% SDS, and 10 μL of 5× SDS loading buffer (Fude, FD002) containing 200 mM DTT (Sigma). The samples were then boiled for 5 min at 98 °C, followed by immunoblotting to detect the tagged proteins.

### sWGA pull-down assay

Mouse brain and culture cells were lysed in RIPA lysis buffer (50 mM Tris-HCl, 150 NaCl, 1 mM EDTA, and 1% Triton X-100, pH 7.4) supplemented with protease and phosphatase inhibitors (Beyotime). Succinylated wheat germ agglutinin (sWGA)-conjugated agarose beads (Vector Laboratories, AL-1023S-2) were incubated with pre-cleared lysates overnight at 4 °C. After five washes with lysis buffer, 1× SDS loading buffer was added to the precipitated complexes, followed by denaturation at 95 °C for 5 min. The target protein input was normalized to a comparable level before performing the sWGA binding assay.

### Statistical analysis

All statistical analyses were conducted using GraphPad Prism software (Ver. 10.2.0). The normality and homogeneity of variance were assumed by the Mann-Whitney U Test or the Kruskal-Wallis Test. Data were analyzed using a two-tailed independent Student’s t-test for comparisons between two groups and a one-way analysis of variance (ANOVA) followed by Tukey’s post-hoc test for comparisons among three or more groups. Unless otherwise stated, data are presented as mean ± SEM. A p-value of < 0.05 was considered statistically significant.

## Electronic supplementary material

Below is the link to the electronic supplementary material.

Supplementary Material 1

Supplementary Material 2

Supplementary Material 3

Supplementary Material 4

Supplementary Material 5

Supplementary Material 6

Supplementary Material 7

Supplementary Material 8

Supplementary Material 9

Supplementary Material 10

Supplementary Material 11

Supplementary Material 12

Supplementary Material 13

Supplementary Material 14

## Data Availability

All data are available in the main text or the supplementary materials. All data generated and analyzed during this study are included in this article or available from the corresponding author on reasonable request.
