# Supplementary figures and images for: Modulation of O-GlcNAc cycling influences α-synuclein amplification, degradation, and associated neuroinflammatory pathology
Source: Mol Neurodegener. 2025 Oct 27;20:113. doi: 10.1186/s13024-025-00904-2 (PMC12560605; doi:10.1186/s13024-025-00904-2)

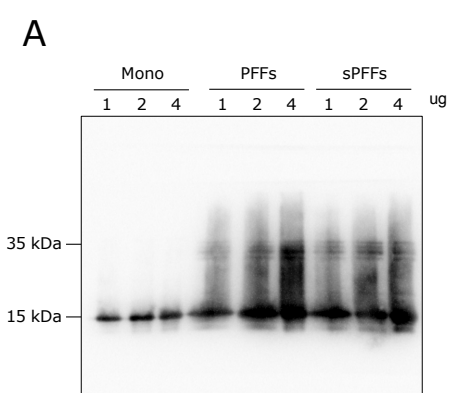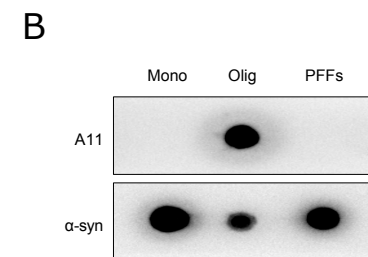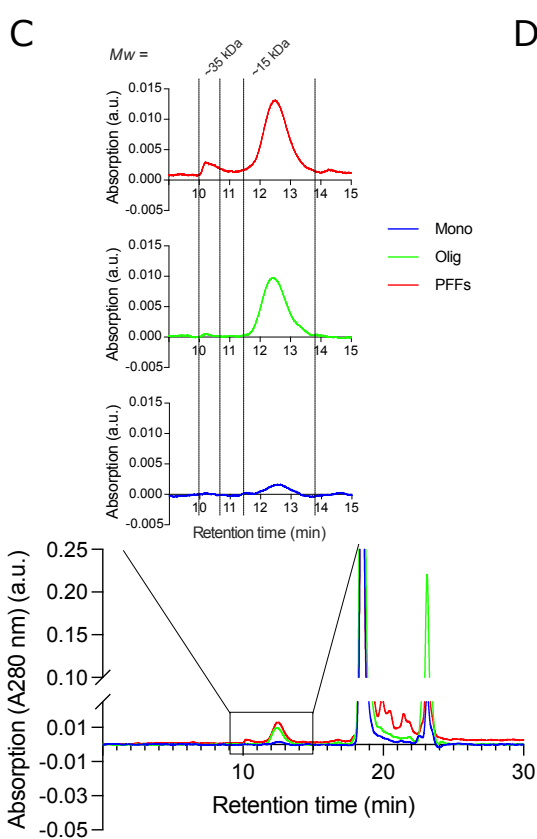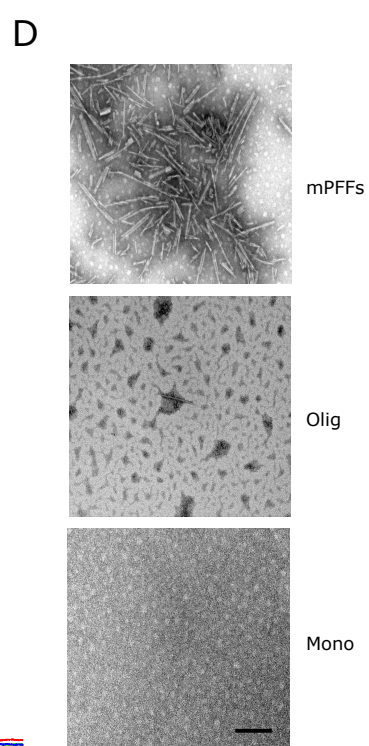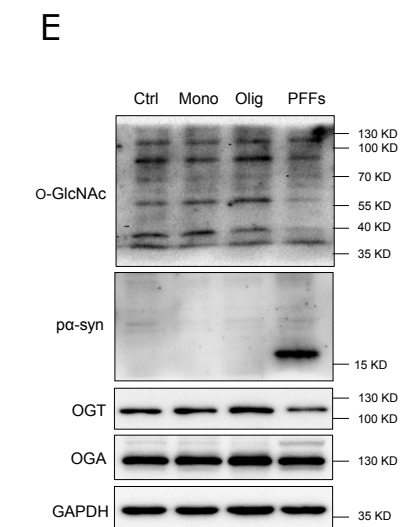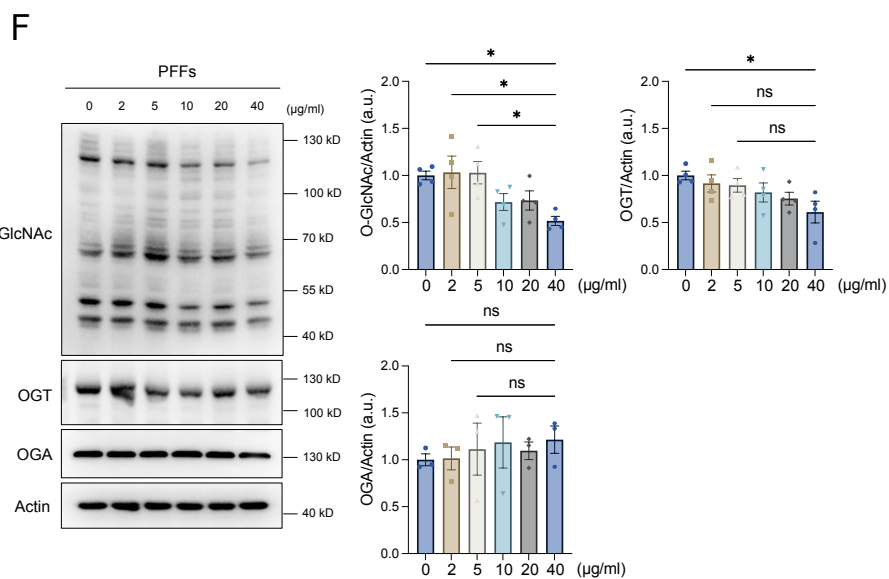

Supplement: Supplementary file 1 — Supplementary Material 1 [file 13024_2025_904_MOESM1_ESM.pdf]

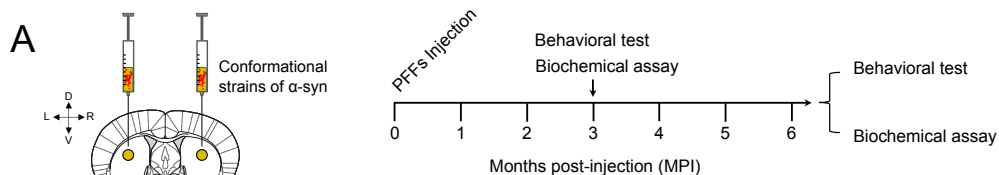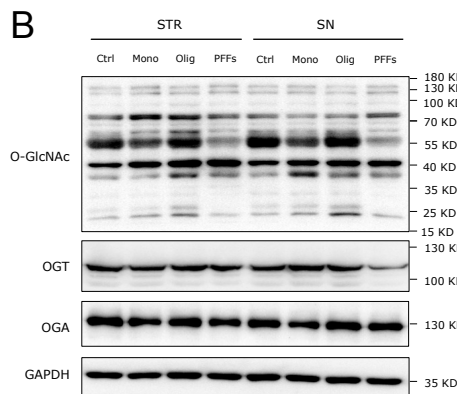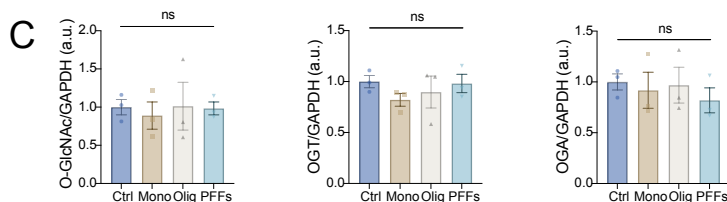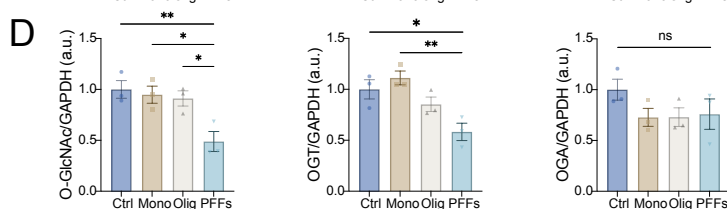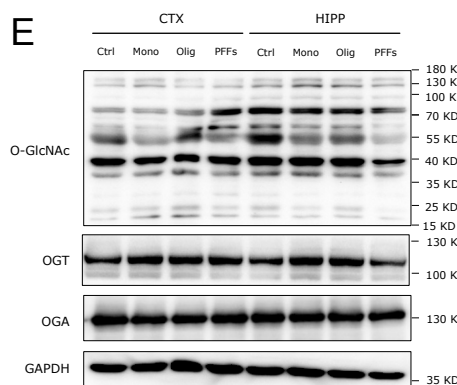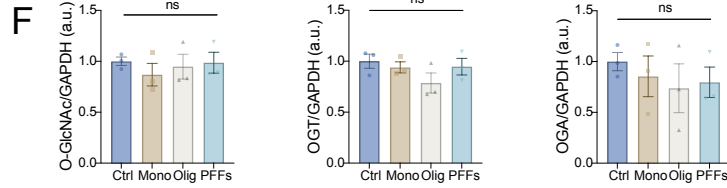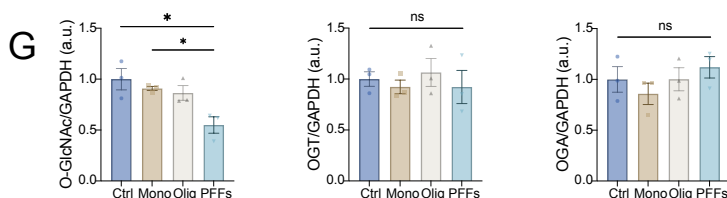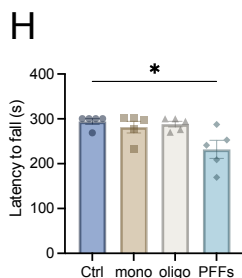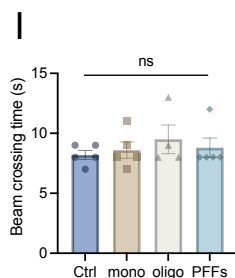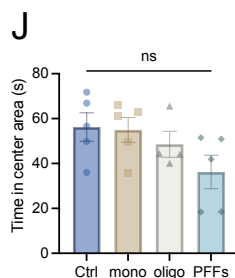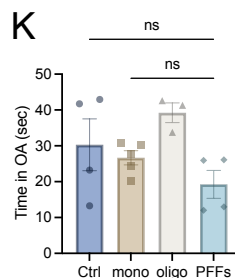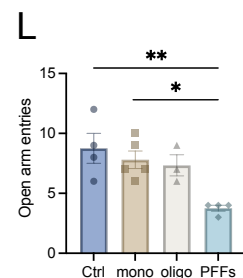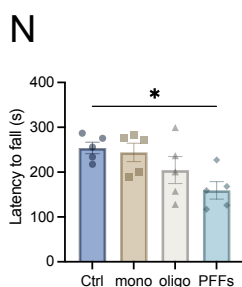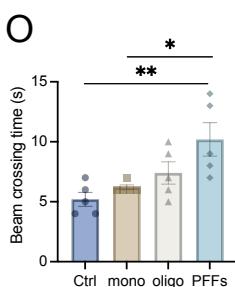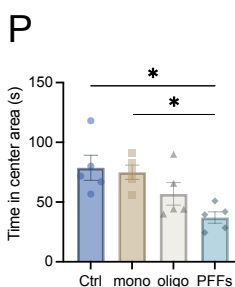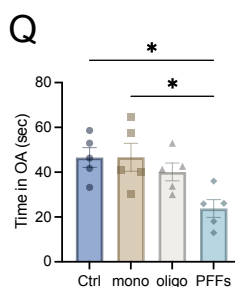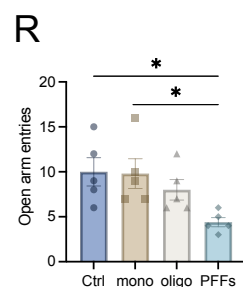

Supplement: Supplementary file 2 — Supplementary Material 2 [file 13024_2025_904_MOESM2_ESM.pdf]

**A**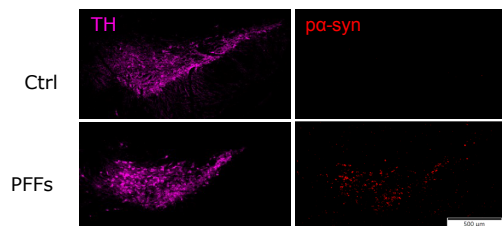**C**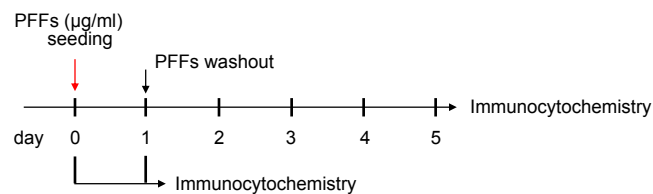**B**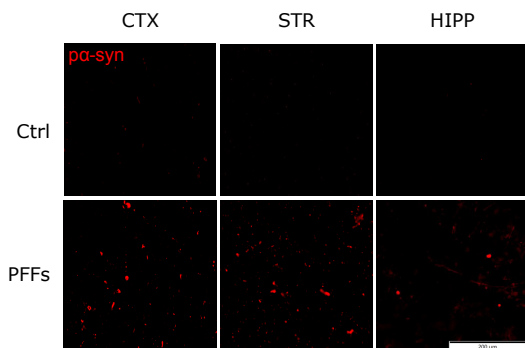**D**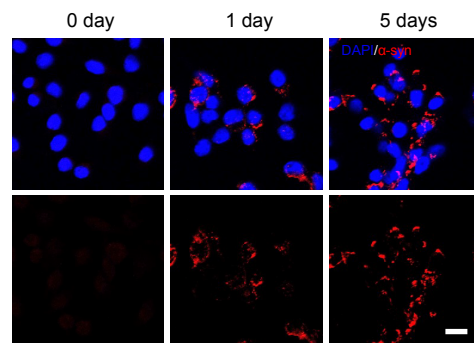**E**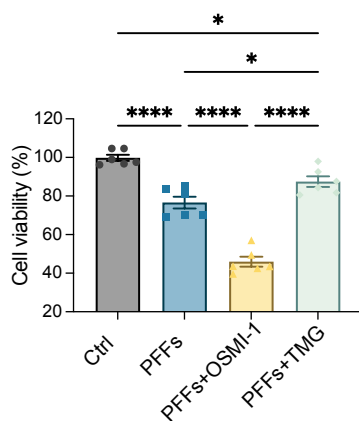**F**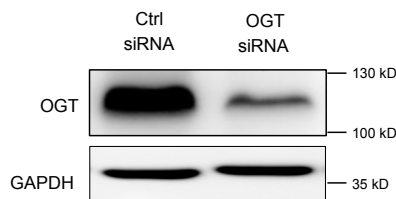**G**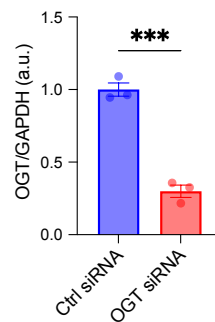**H**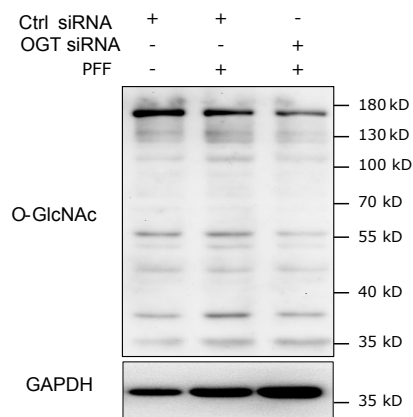**I**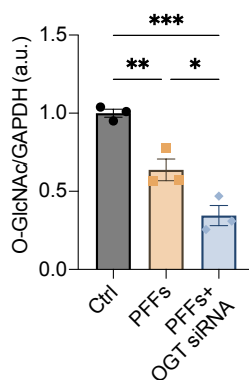

Supplement: Supplementary file 3 — Supplementary Material 3 [file 13024_2025_904_MOESM3_ESM.pdf]

**A**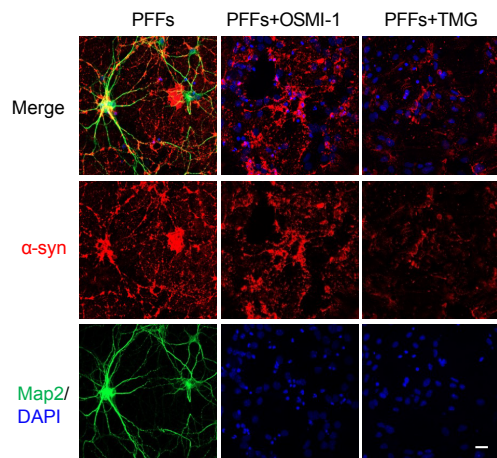**B**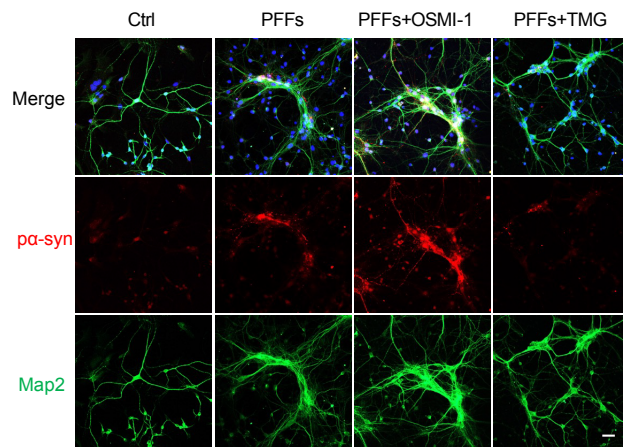**C**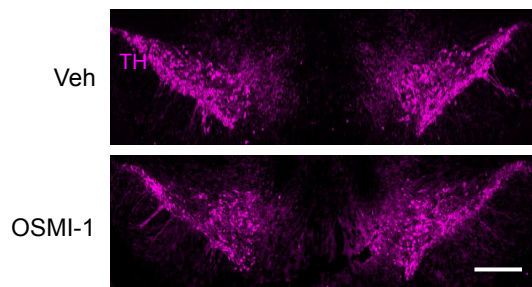**D**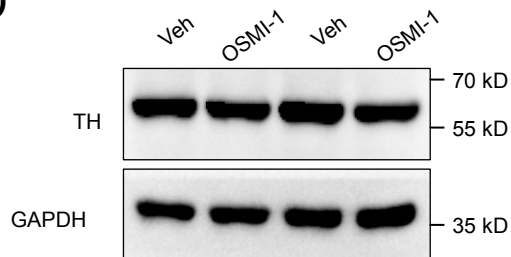

Supplement: Supplementary file 4 — Supplementary Material 4 [file 13024_2025_904_MOESM4_ESM.pdf]

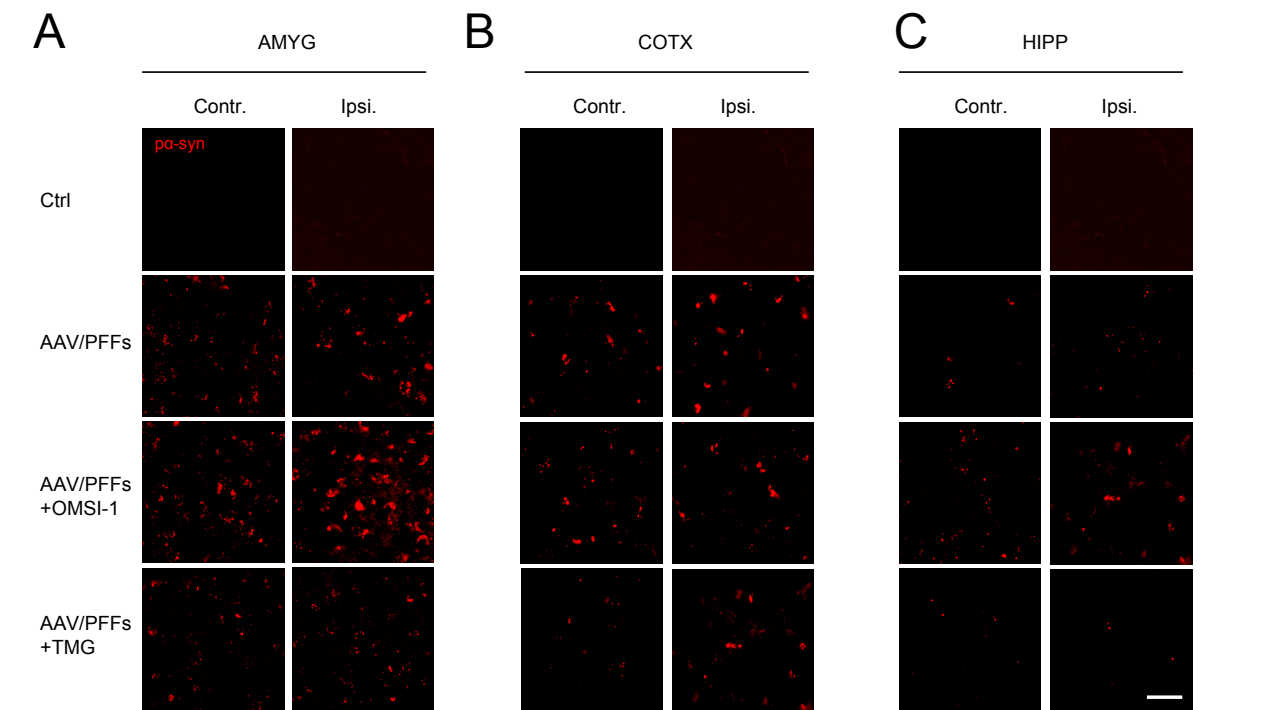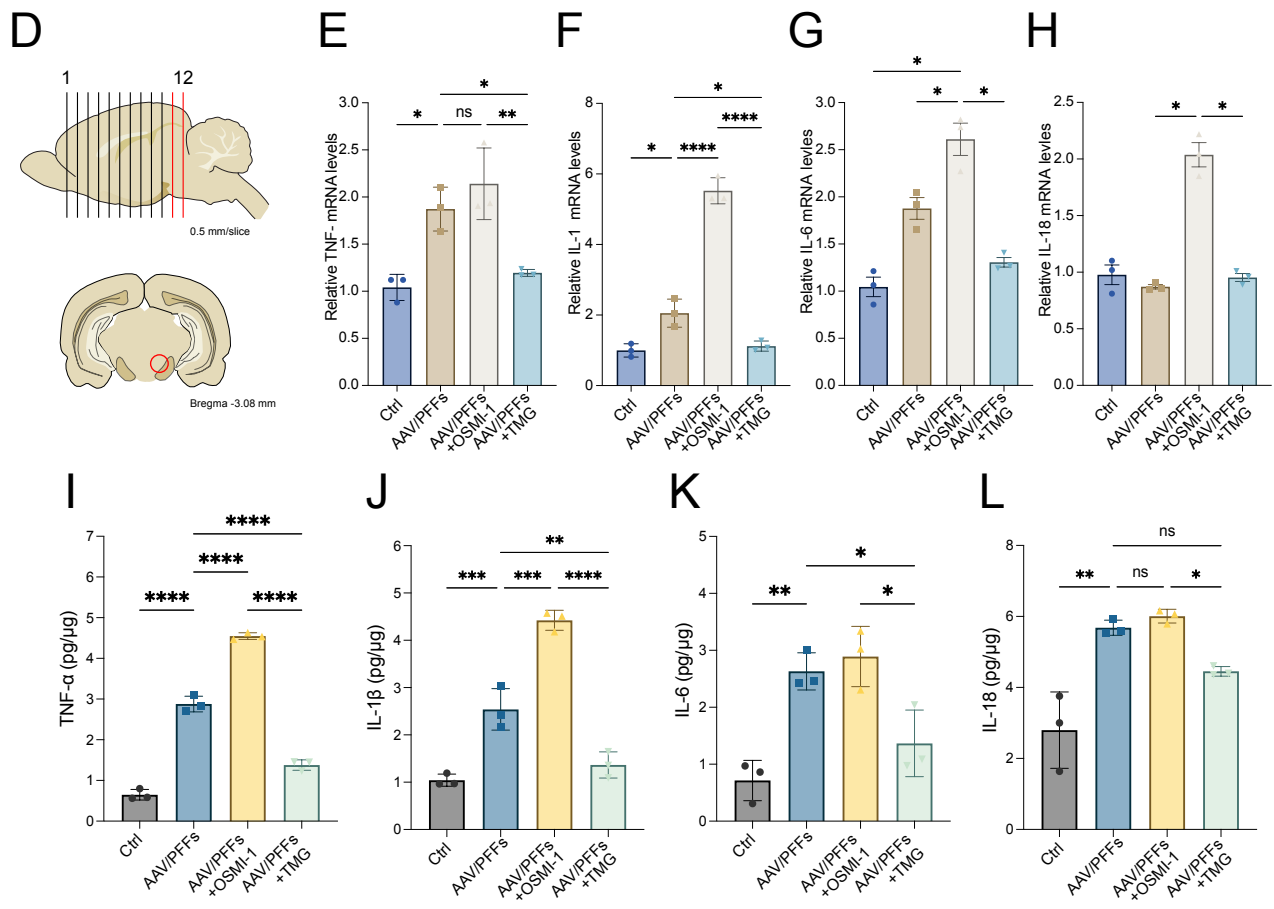

Supplement: Supplementary file 7 — Supplementary Material 7 [file 13024_2025_904_MOESM7_ESM.pdf]

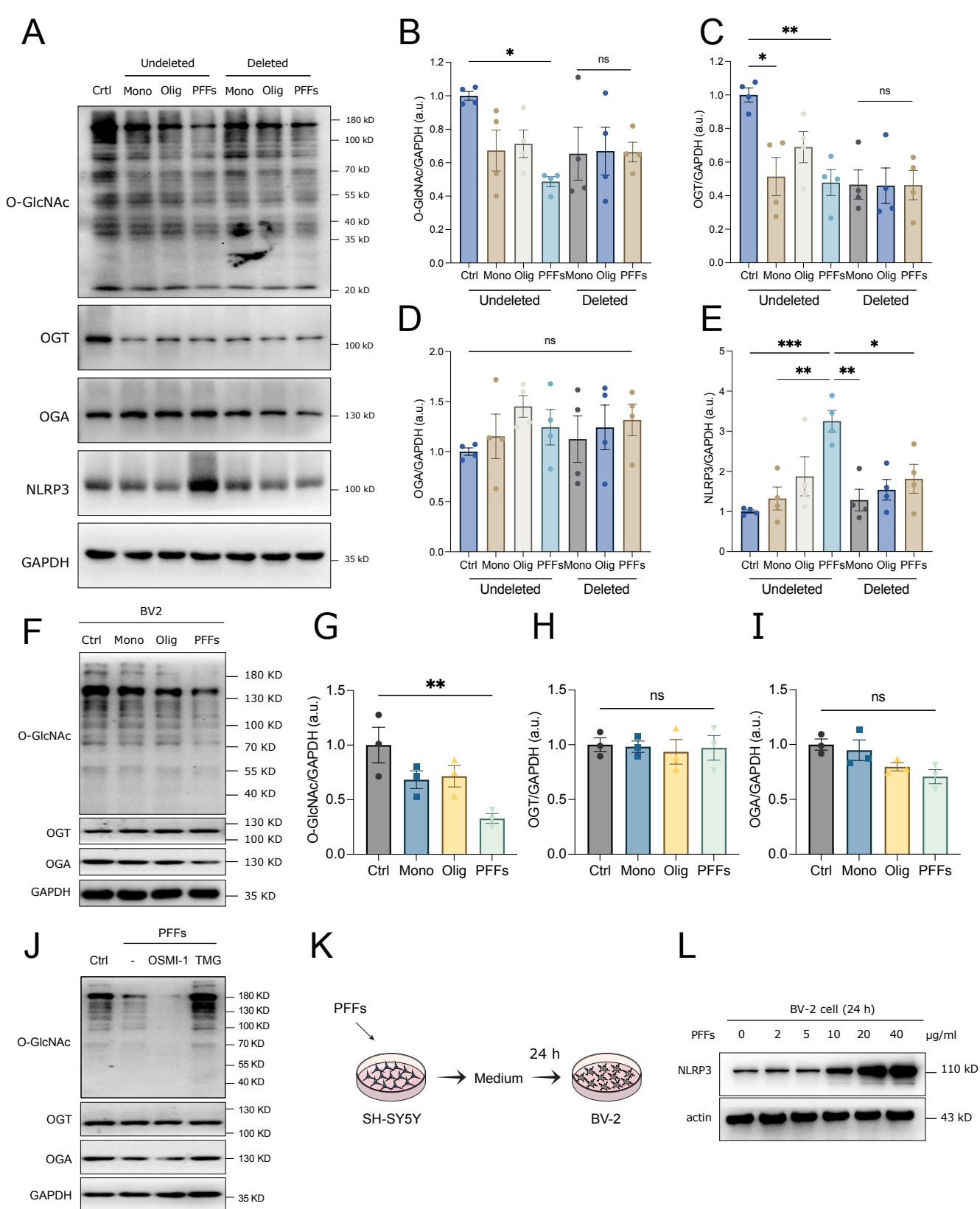

Supplement: Supplementary file 8 — Supplementary Material 8 [file 13024_2025_904_MOESM8_ESM.pdf]

**A**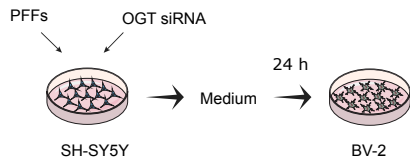**B**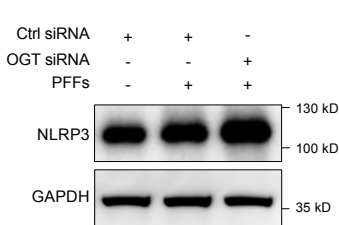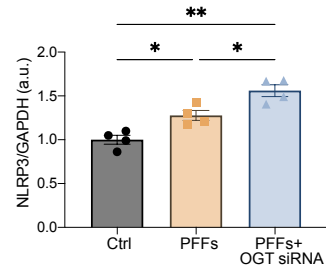**C**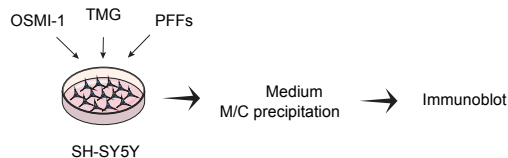**D**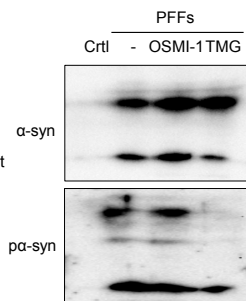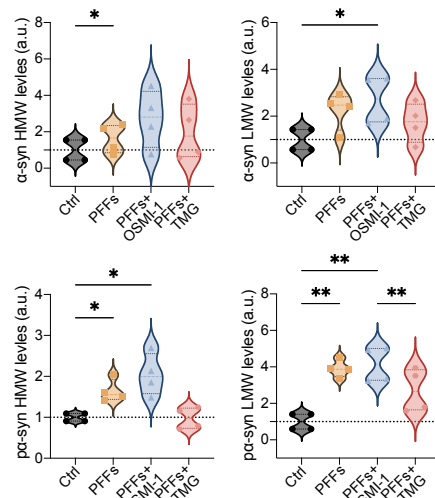**E**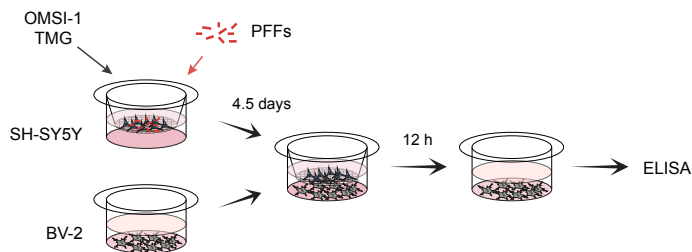**F**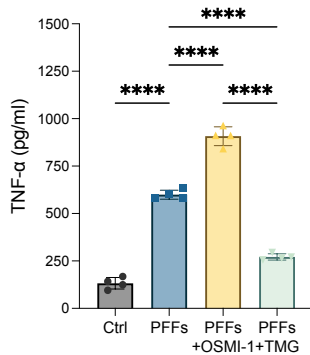**G**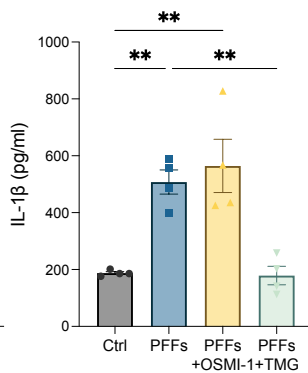**H**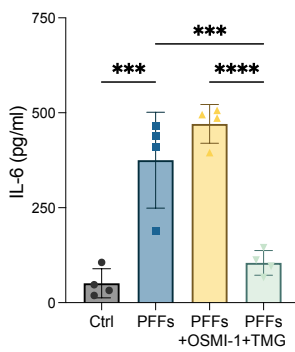**I**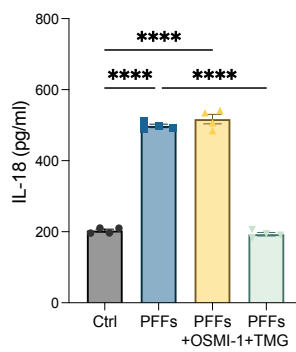

Supplement: Supplementary file 9 — Supplementary Material 9 [file 13024_2025_904_MOESM9_ESM.pdf]

A

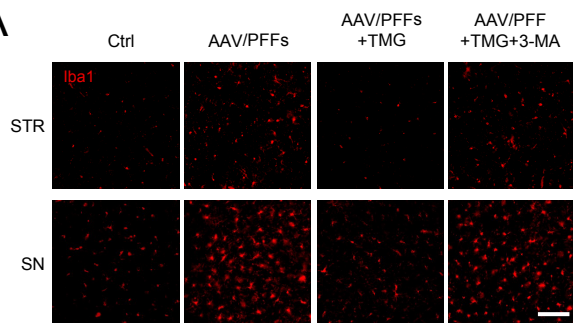

B

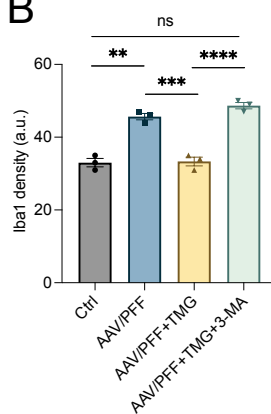

C

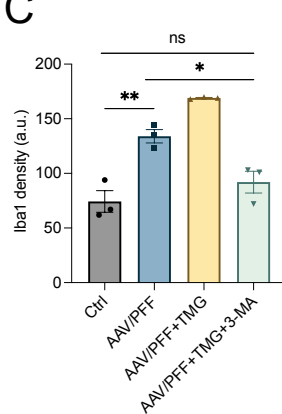

D

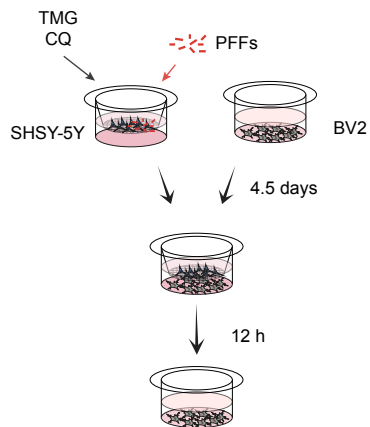

E

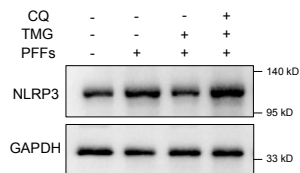

F

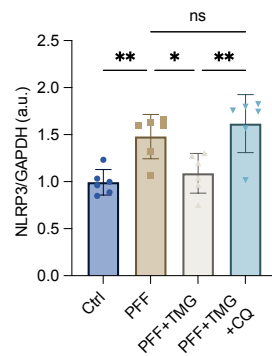

Supplement: Supplementary file 10 — Supplementary Material 10 [file 13024_2025_904_MOESM10_ESM.pdf]

A

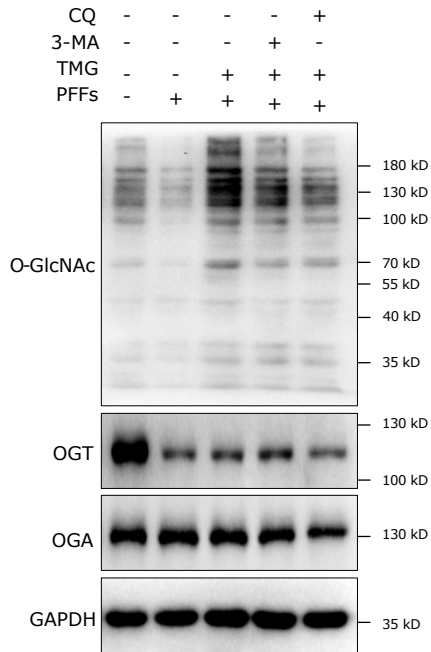

B

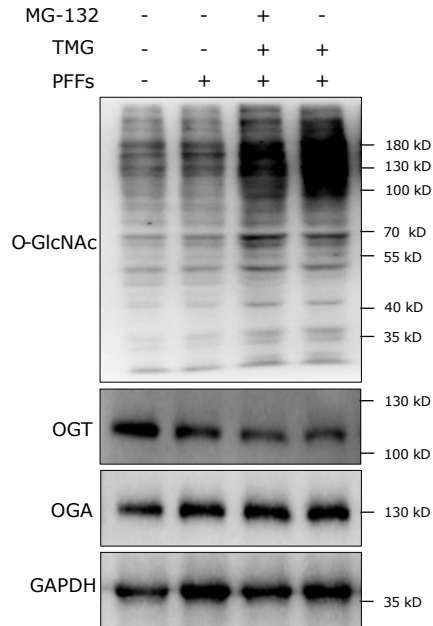

Supplement: Supplementary file 11 — Supplementary Material 11 [file 13024_2025_904_MOESM11_ESM.pdf]

## Reduced O-GlcNAcylation

## PD with $\alpha$ -syn aggregation

## Enhanced O-GlcNAcylation

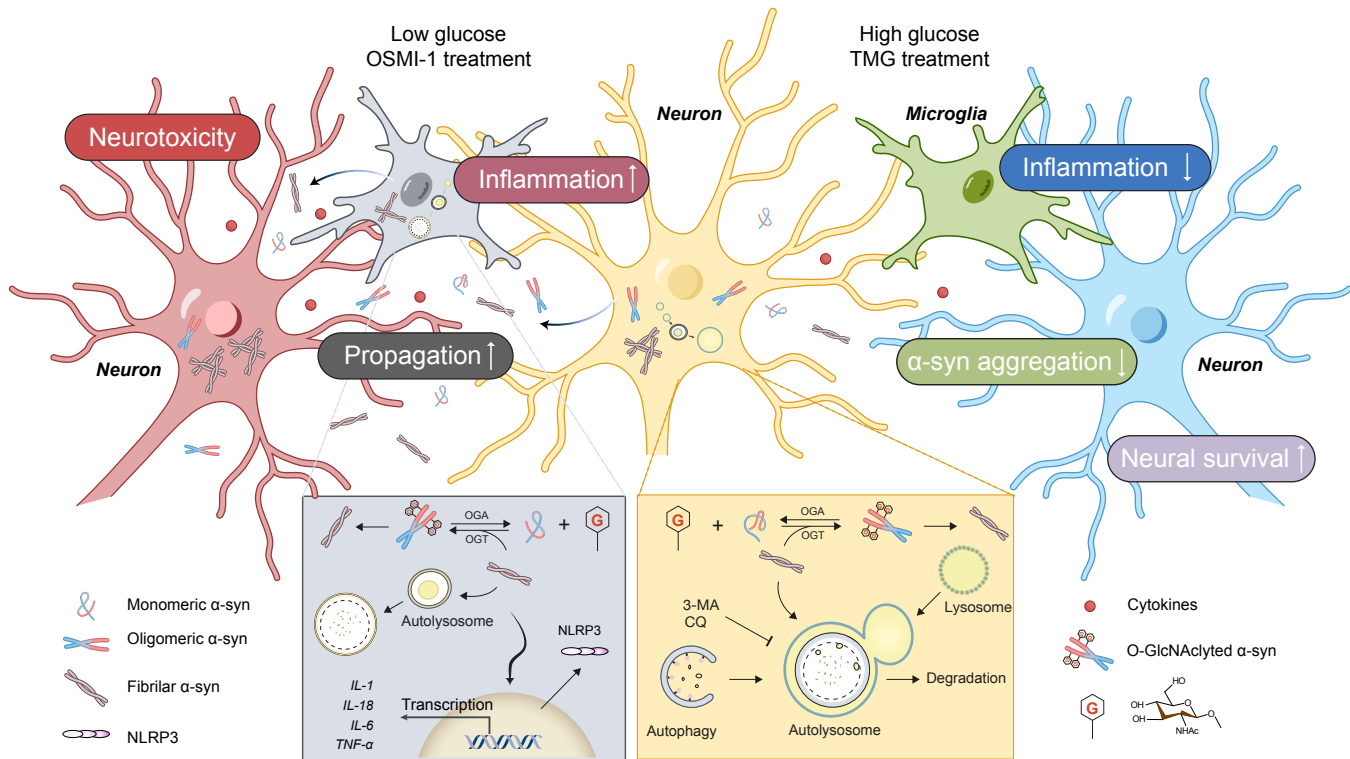

Supplement: Supplementary file 12 — Supplementary Material 12 [file 13024_2025_904_MOESM12_ESM.pdf]
