# Supplementary material for: Modulation of O-GlcNAc cycling influences α-synuclein amplification, degradation, and associated neuroinflammatory pathology
Source: Mol Neurodegener. 2025 Oct 27;20:113. doi: 10.1186/s13024-025-00904-2 (PMC12560605; doi:10.1186/s13024-025-00904-2)
Supplement: Supplementary file 5 — Supplementary Material 5 [file 13024_2025_904_MOESM5_ESM.pdf]

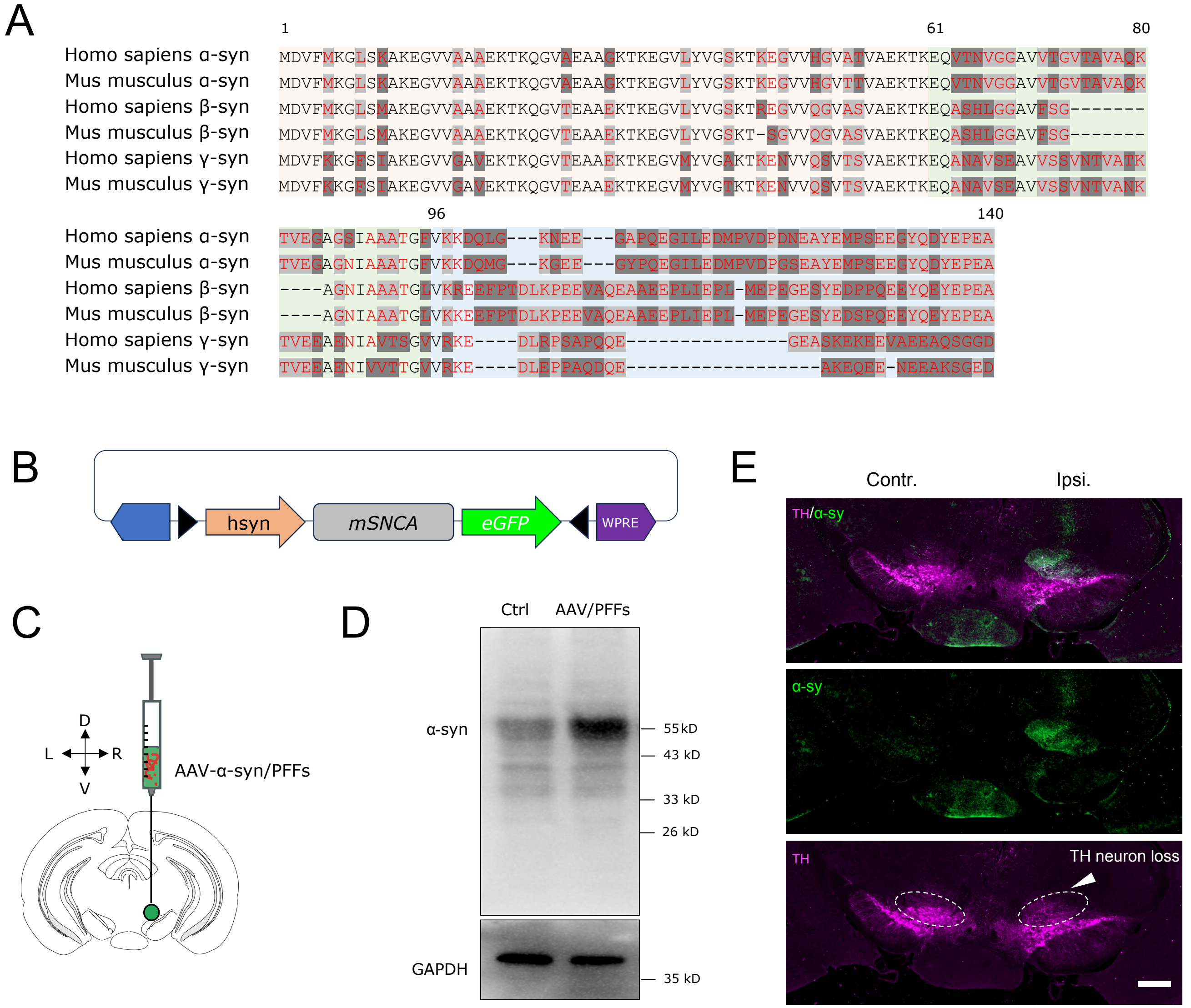
A
1
61
80
Homo sapiens α-syn MDVFMKGLSKAKEGVVAAAEKTKQGVAEAAGKTKEGVLYVGSKTKEGVVHGVATVAEKTKEQVTNVGGAVVTGVTAVAQK
Mus musculus α-syn MDVFMKGLSKAKEGVVAAAEKTKQGVAEAAGKTKEGVLYVGSKTKEGVVHGVTTVAEKTKEQVTNVGGAVVTGVTAVAQK
Homo sapiens β-syn MDVFMKGLSMAKEGVVAAAEKTKQGVTEAAEKTKEGVLYVGSKTREGVVQGVASVAEKTKEQASHLGGAVFSG-------
Mus musculus β-syn MDVFMKGLSMAKEGVVAAAEKTKQGVTEAAEKTKEGVLYVGSKT-SGVVQGVASVAEKTKEQASHLGGAVFSG-------
Homo sapiens γ-syn MDVFKKGFSIAKEGVVGAVEKTKQGVTEAAEKTKEGVMYVGAKTKENVVQSVTSVAEKTKEQANAVSEAVVSSVNTVATK
Mus musculus γ-syn MDVFKKGFSIAKEGVVGAVEKTKQGVTEAAEKTKEGVMYVGTKTKENVVQSVTSVAEKTKEQANAVSEAVVSSVNTVANK
96
140
Homo sapiens α-syn TVEGAGSIAAATGFVKKDQLG---KNEE---GAPQEGILEDMPVDPDNEAYEMPSEEGYQDYEPEA
Mus musculus α-syn TVEGAGNIAAATGFVKKDQMG---KGEE---GYPQEGILEDMPVDPGSEAYEMPSEEGYQDYEPEA
Homo sapiens β-syn ----AGNIAAATGLVKREEFPTDLKPEEVAQEAAEEPLIEPL-MEPEGESYEDPPQEEYQEYEPEA
Mus musculus β-syn ----AGNIAAATGLVKKEEFPTDLKPEEVAQEAAEEPLIEPL-MEPEGESYEDSPQEEYQEYEPEA
Homo sapiens γ-syn TVEEAENIAVTSGVVRKE----DLRPSAPQQE---------------GEASKEKEEVAEEAQSGGD
Mus musculus γ-syn TVEEAENIVVTTGVVRKE----DLEPPAQDQE------------------AKEQEE-NEEAKSGED
B
hsyn
mSNCA
eGFP
WPRE
C
D
L
R
V
AAV-α-syn/PFFs
D
Ctrl
AAV/PFFs
α-syn
55 kD
43 kD
33 kD
26 kD
GAPDH
35 kD
E
Contr.
Ipsi.
TH/α-sy
α-sy
TH
TH neuron loss
