# Supplementary material for: Modulation of O-GlcNAc cycling influences α-synuclein amplification, degradation, and associated neuroinflammatory pathology
Source: Mol Neurodegener. 2025 Oct 27;20:113. doi: 10.1186/s13024-025-00904-2 (PMC12560605; doi:10.1186/s13024-025-00904-2)
Supplement: Supplementary file 6 — Supplementary Material 6 [file 13024_2025_904_MOESM6_ESM.pdf]

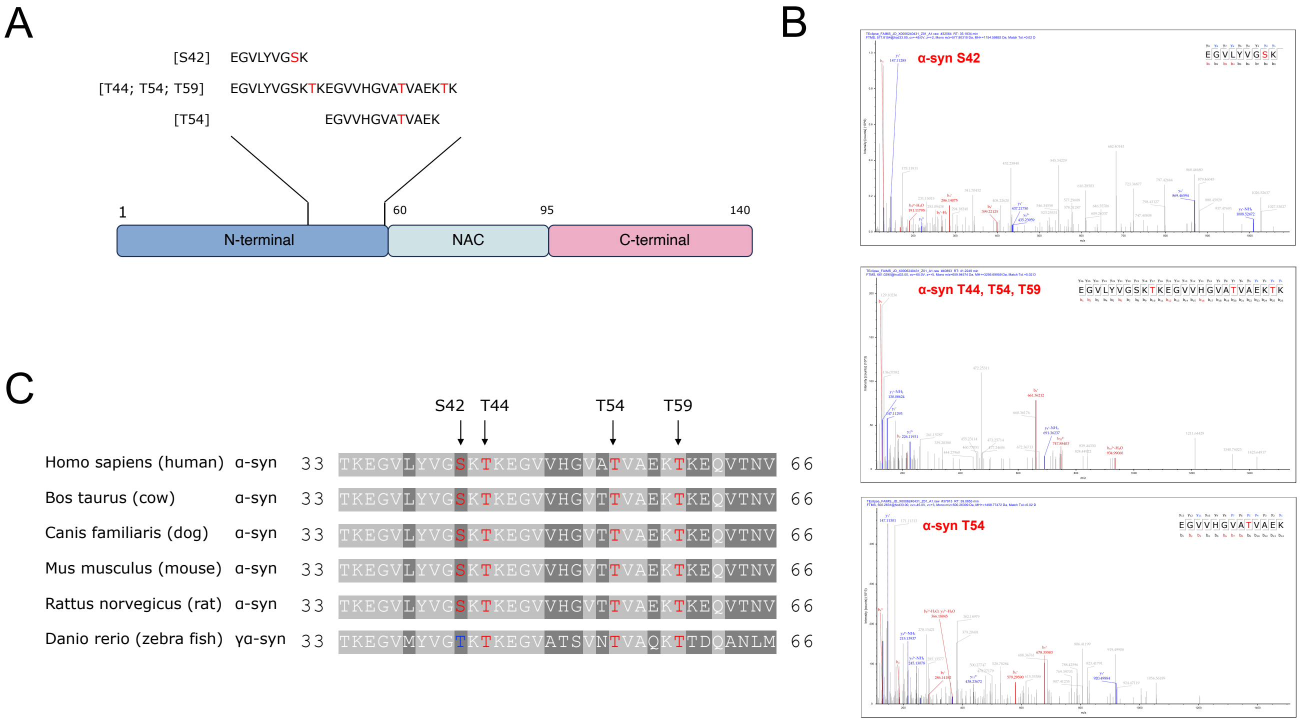
A
[S42] EGVLYVGSK
[T44; T54; T59] EGVLYVGSKTKEGVVHGVATVAEKTK
[T54] EGVVHGVATVAEK
1
60
95
140
N-terminal
NAC
C-terminal
B
α-syn S42
EGVLYVGSK
α-syn T44, T54, T59
EGVLYVGSKTKEGVVHGVATVAEKTK
α-syn T54
EGVVHGVATVAEK
C
S42
T44
T54
T59
Homo sapiens (human) α-syn 33 TKEGVLYVGSKTKEGVVHGVATVAEKTKEQVTNV 66
Bos taurus (cow) α-syn 33 TKEGVLYVGSKTKEGVVHGVTTVAEKTKEQVTNV 66
Canis familiaris (dog) α-syn 33 TKEGVLYVGSKTKEGVVHGVTTVAEKTKEQVTNV 66
Mus musculus (mouse) α-syn 33 TKEGVLYVGSKTKEGVVHGVTTVAEKTKEQVTNV 66
Rattus norvegicus (rat) α-syn 33 TKEGVLYVGSKTKEGVVHGVTTVAEKTKEQVTNV 66
Danio rerio (zebra fish) γα-syn 33 TKEGVMYVGTKTKEGVATSVNTVAQKTTDQANLM 66
