# Supplementary material for: Modulation of O-GlcNAc cycling influences α-synuclein amplification, degradation, and associated neuroinflammatory pathology
Source: Mol Neurodegener. 2025 Oct 27;20:113. doi: 10.1186/s13024-025-00904-2 (PMC12560605; doi:10.1186/s13024-025-00904-2)

Fig 1A

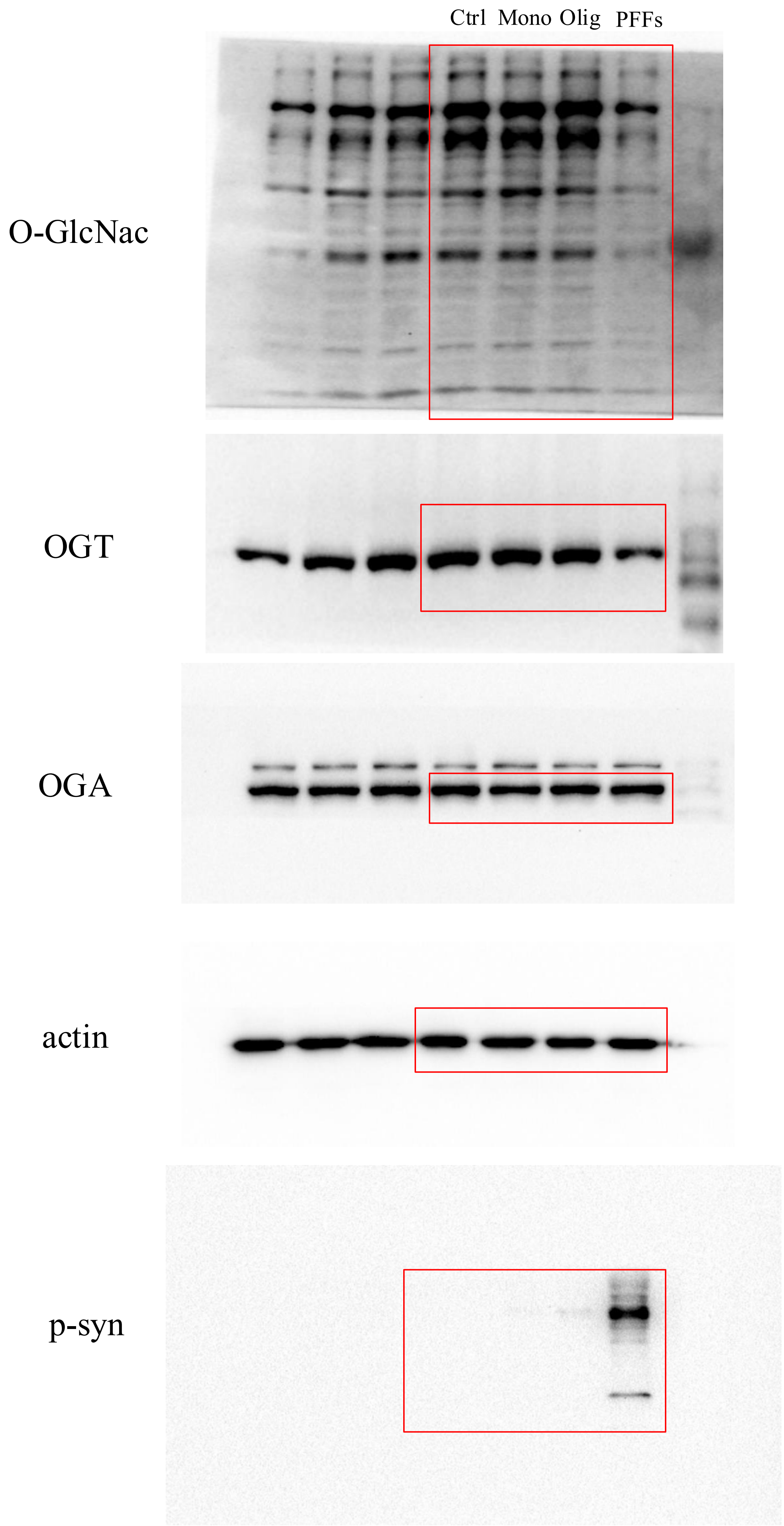
Ctrl
Mono
Olig
PFFs
O-GlcNac
OGT
OGA
actin
p-syn

Fig 1B

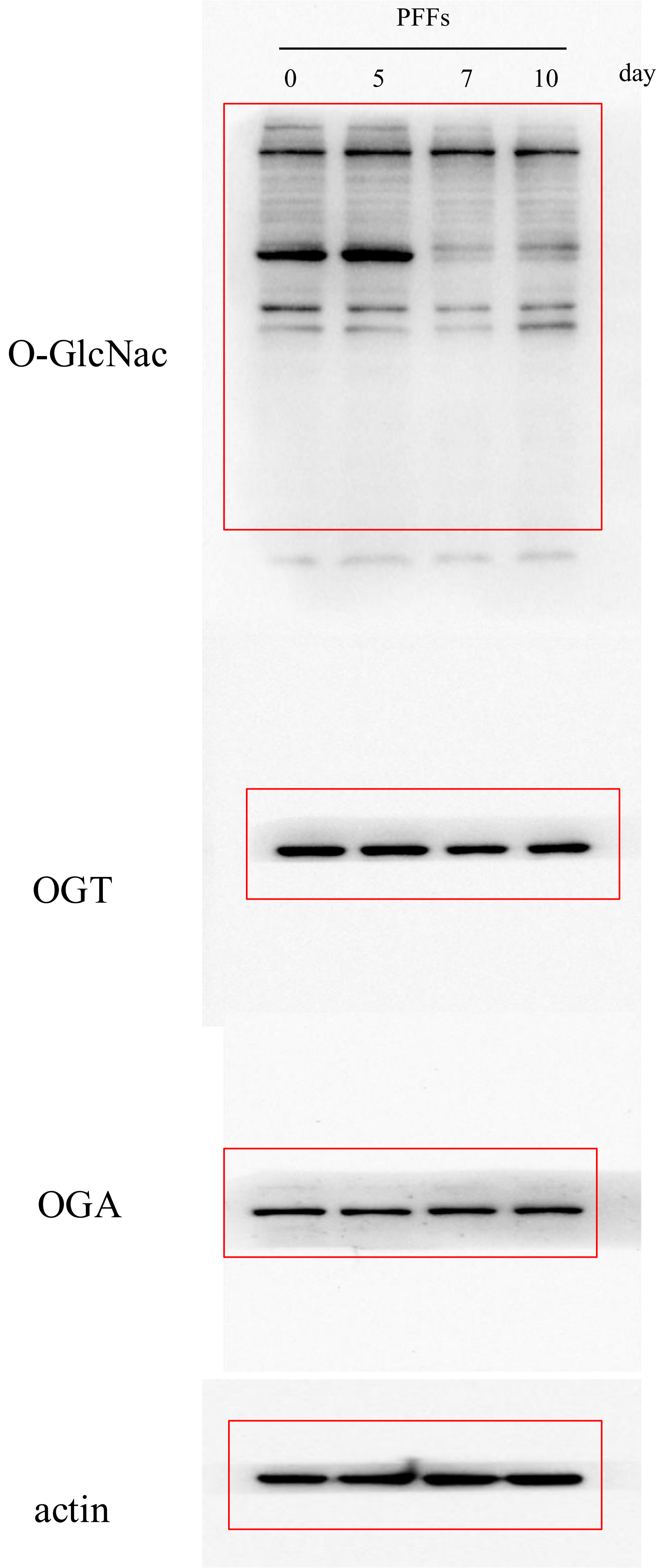
PFFs
0
5
7
10
day
O-GlcNac
OGT
OGA
actin

Fig 1C

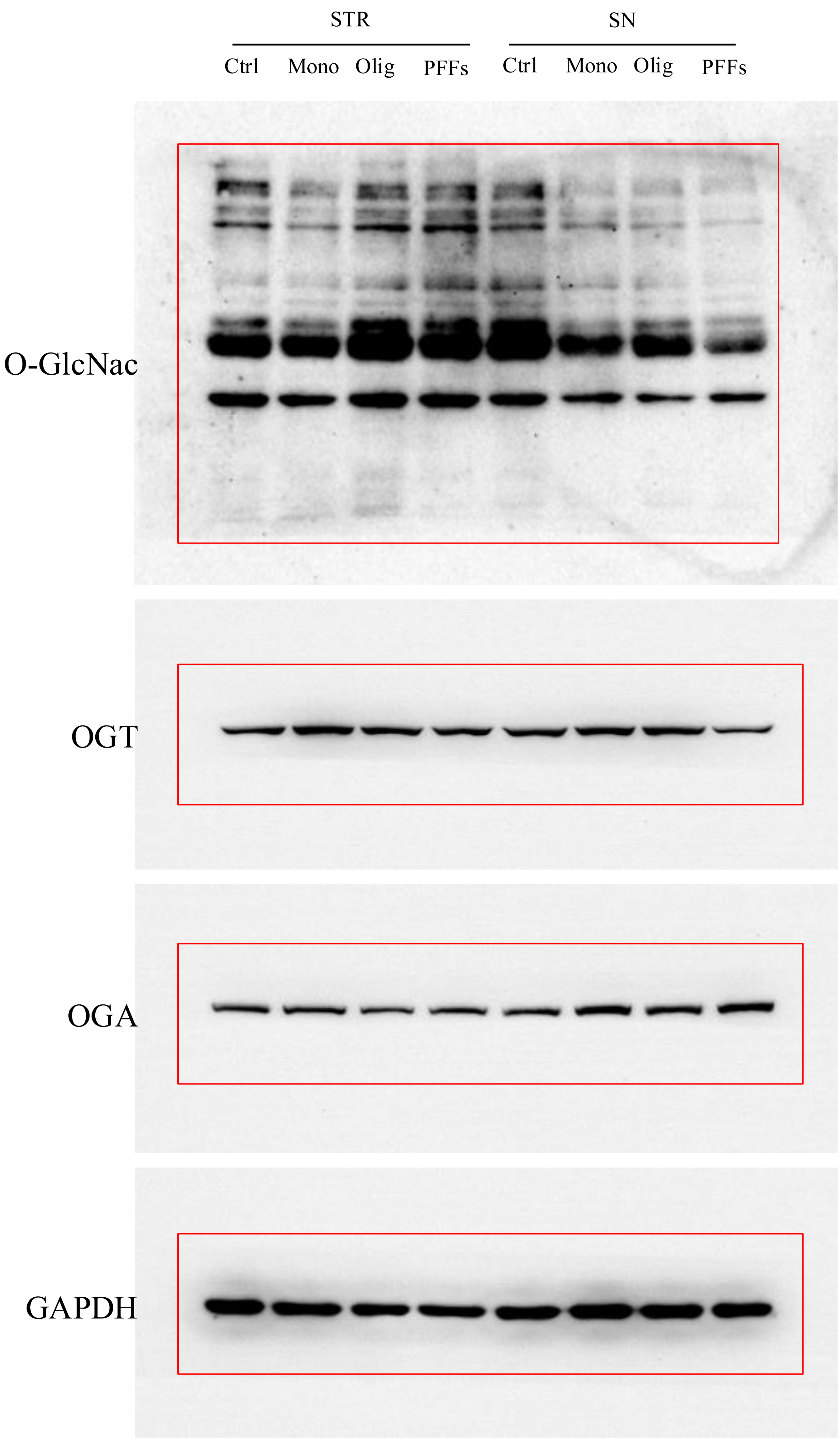
STR
SN
Ctrl
Mono
Olig
PFFs
Ctrl
Mono
Olig
PFFs
O-GlcNac
OGT
OGA
GAPDH

Fig 1F

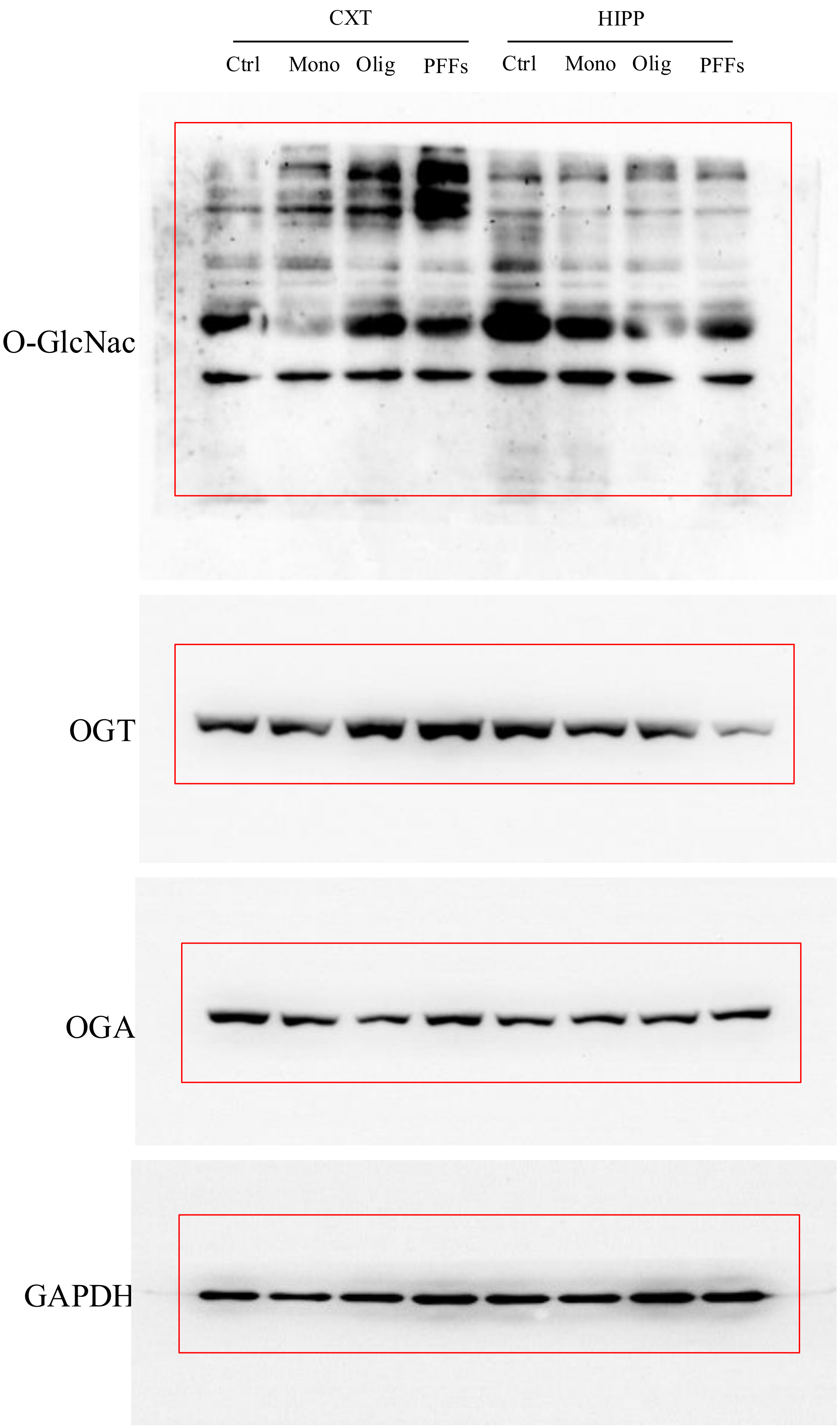
CXT
HIPP
Ctrl
Mono
Olig
PFFs
Ctrl
Mono
Olig
PFFs
O-GlcNac
OGT
OGA
GAPDH

Fig 2A

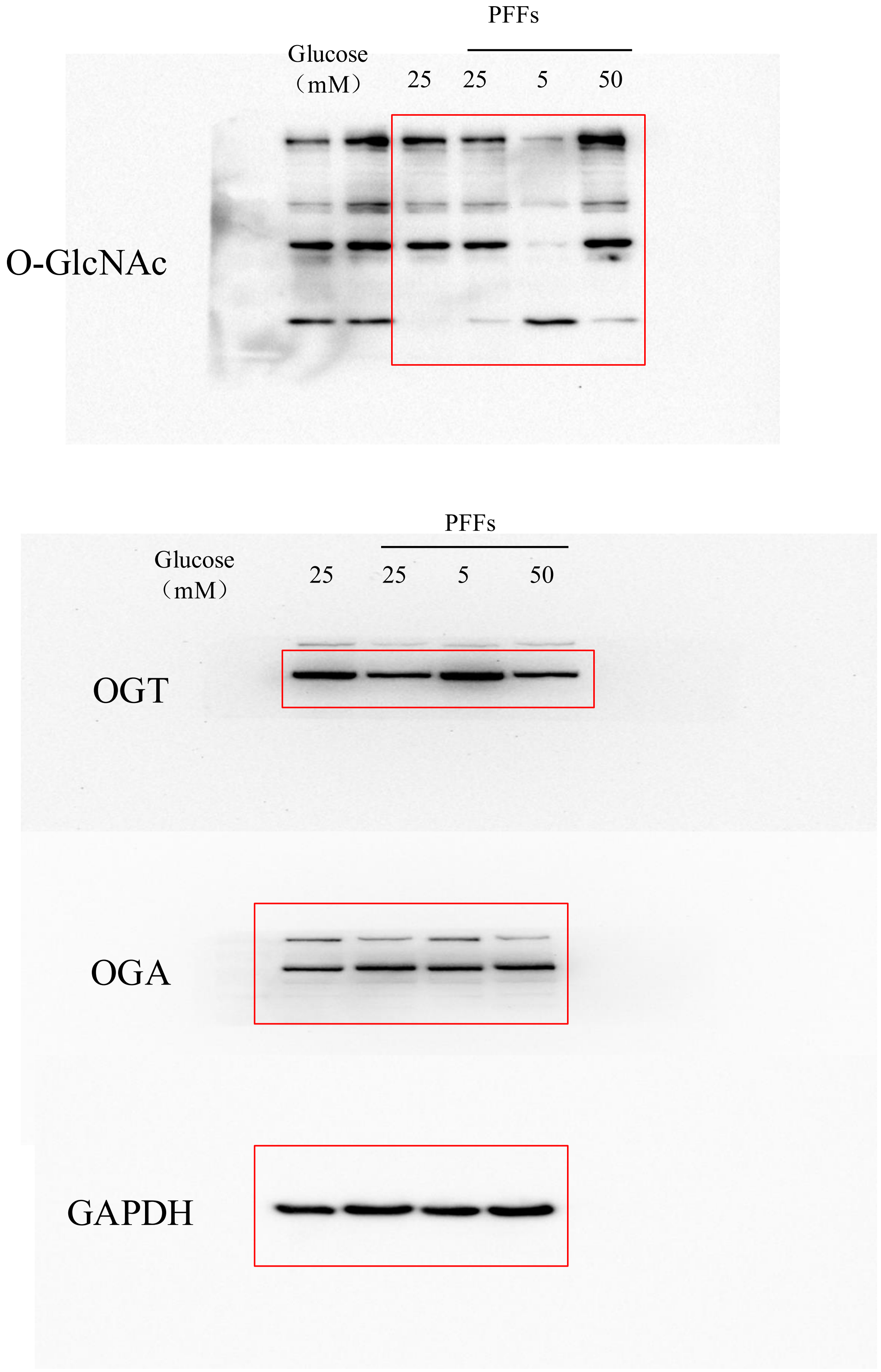

PFFs
Glucose (mM)
25
25
5
50
O-GlcNAc
PFFs
Glucose (mM)
25
25
5
50
OGT
OGA
GAPDH


Fig 2B

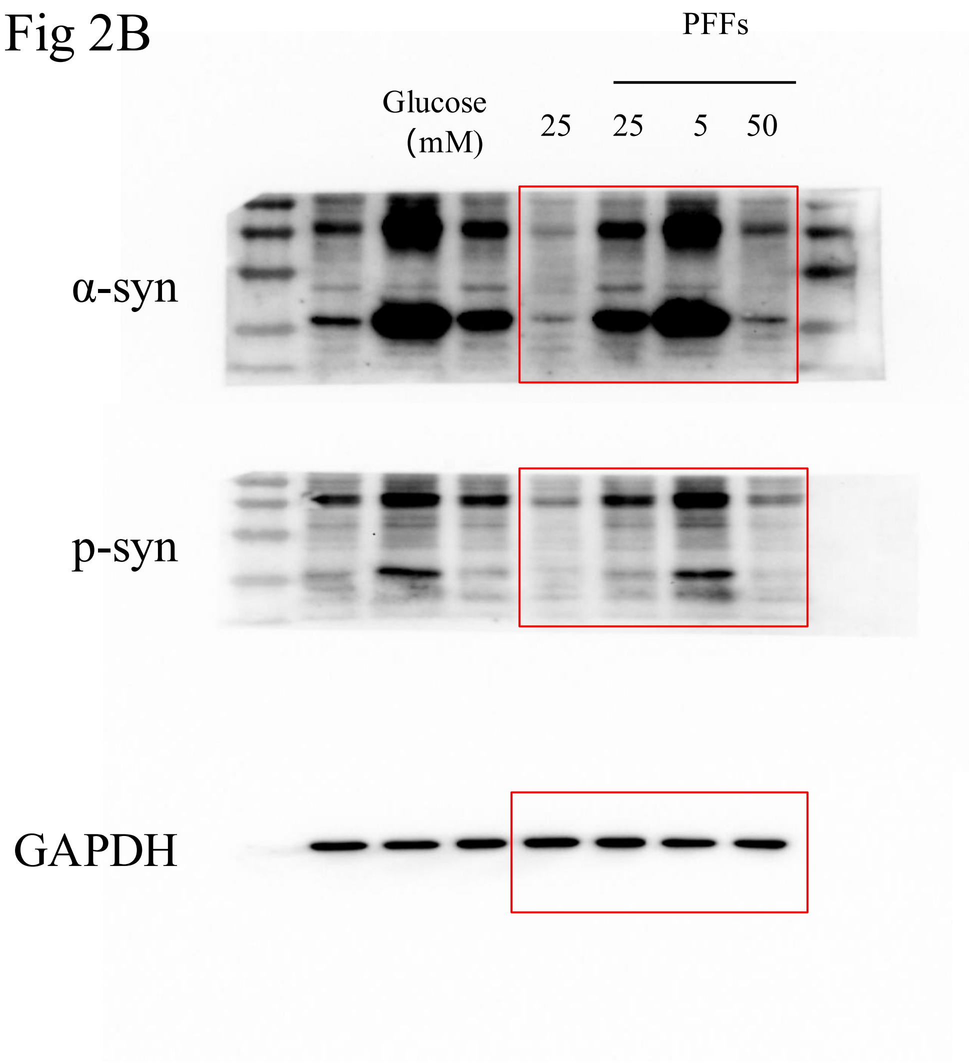

PFFs
Glucose (mM)
25
25
5
50
α-syn
p-syn
GAPDH

Fig 2E

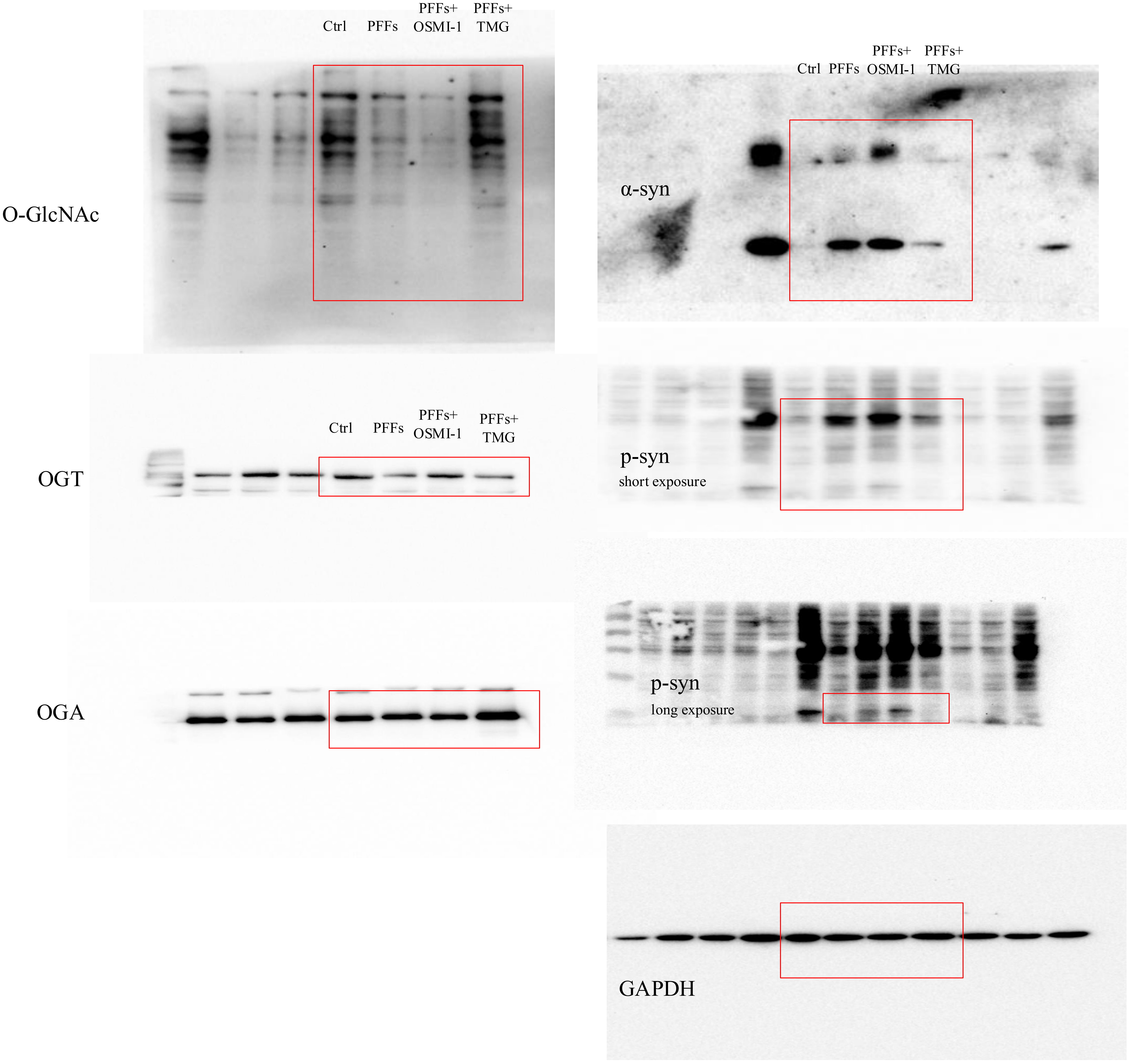

Ctrl
PFFs
PFFs+ OSMI-1
PFFs+ TMG
O-GlcNAc
α-syn
p-syn
short exposure
OGT
OGA
p-syn
long exposure
GAPDH

Fig 2K

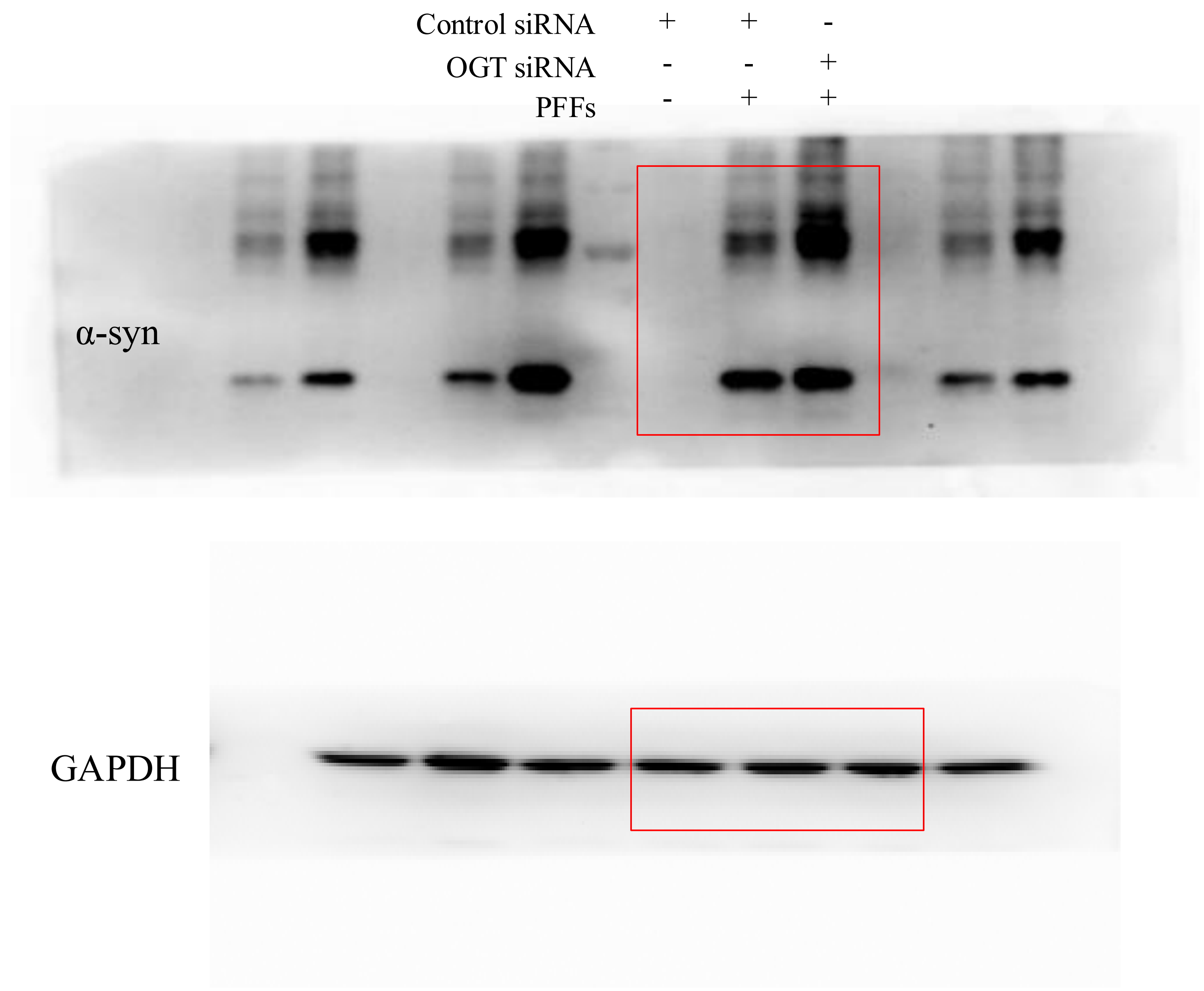

Fig 2L

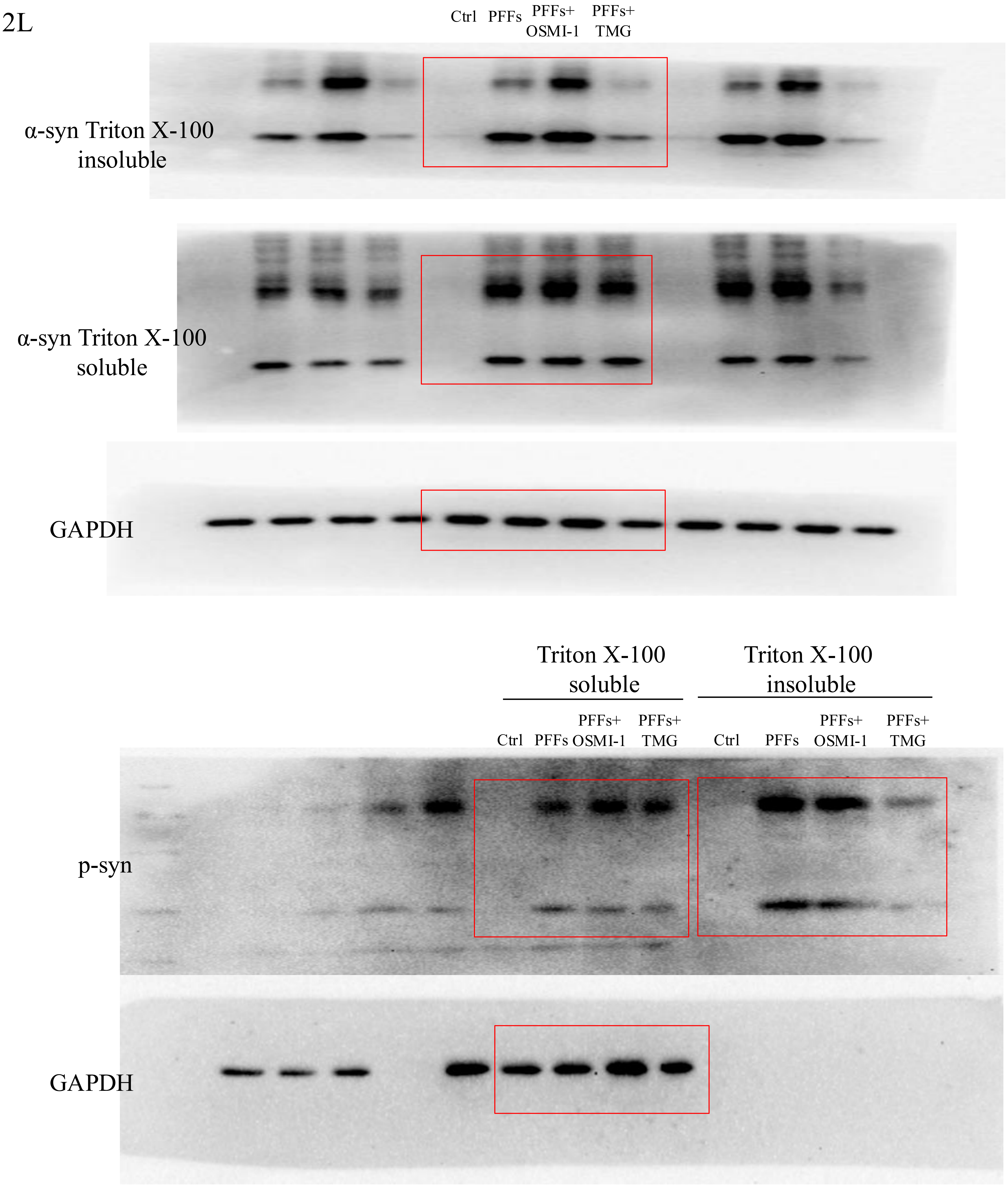
Ctrl
PFFs
PFFs+ OSMI-1
PFFs+ TMG
α-syn Triton X-100 insoluble
α-syn Triton X-100 soluble
GAPDH
Triton X-100 soluble
Triton X-100 insoluble
Ctrl
PFFs
PFFs+ OSMI-1
PFFs+ TMG
Ctrl
PFFs
PFFs+ OSMI-1
PFFs+ TMG
p-syn
GAPDH

Fig 3G

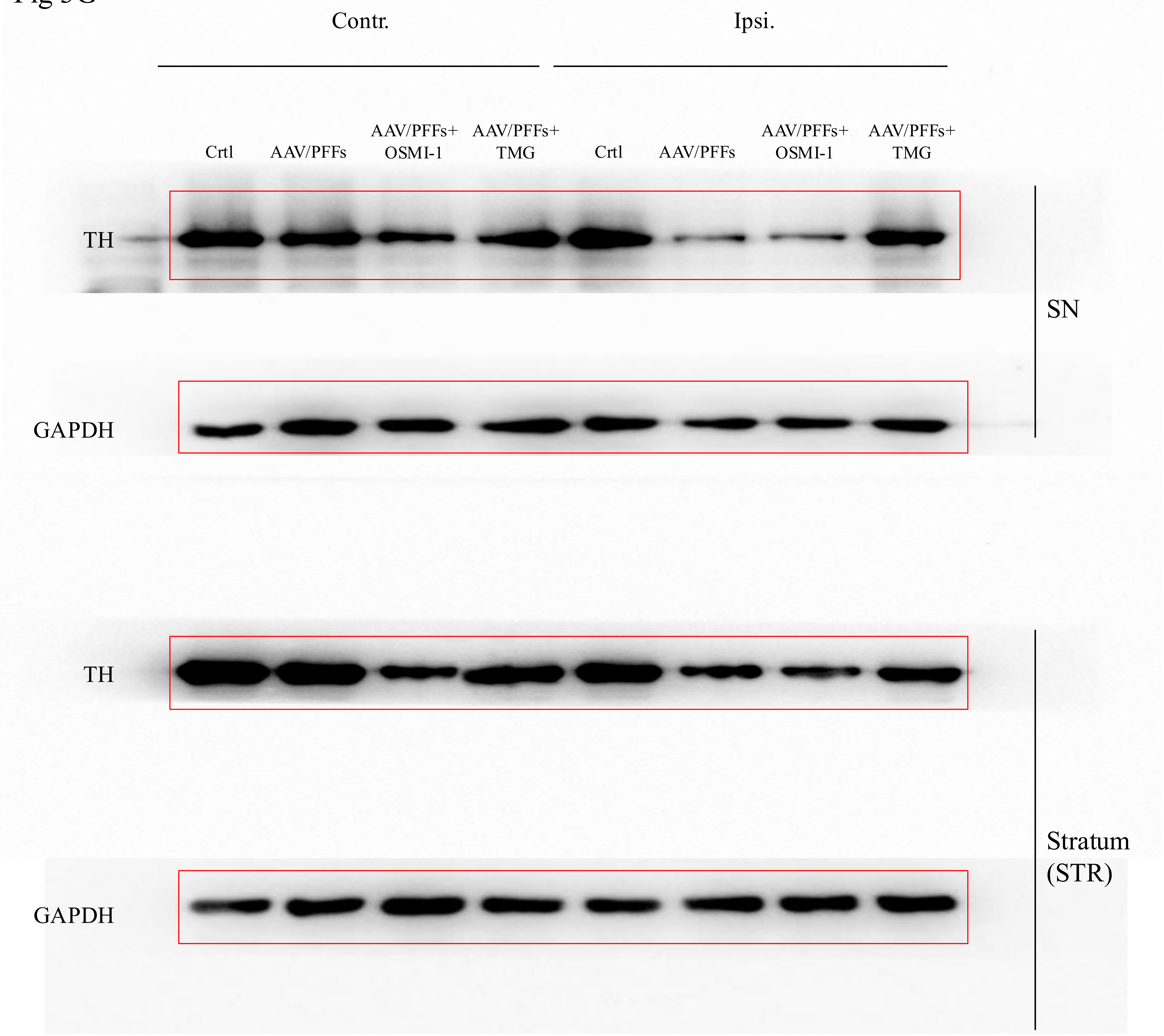
Contr.
Ipsi.
Crtl
AAV/PFFs
AAV/PFFs+ OSMI-1
AAV/PFFs+ TMG
Crtl
AAV/PFFs
AAV/PFFs+ OSMI-1
AAV/PFFs+ TMG
TH
GAPDH
SN
TH
GAPDH
Stratum (STR)

Fig 4B

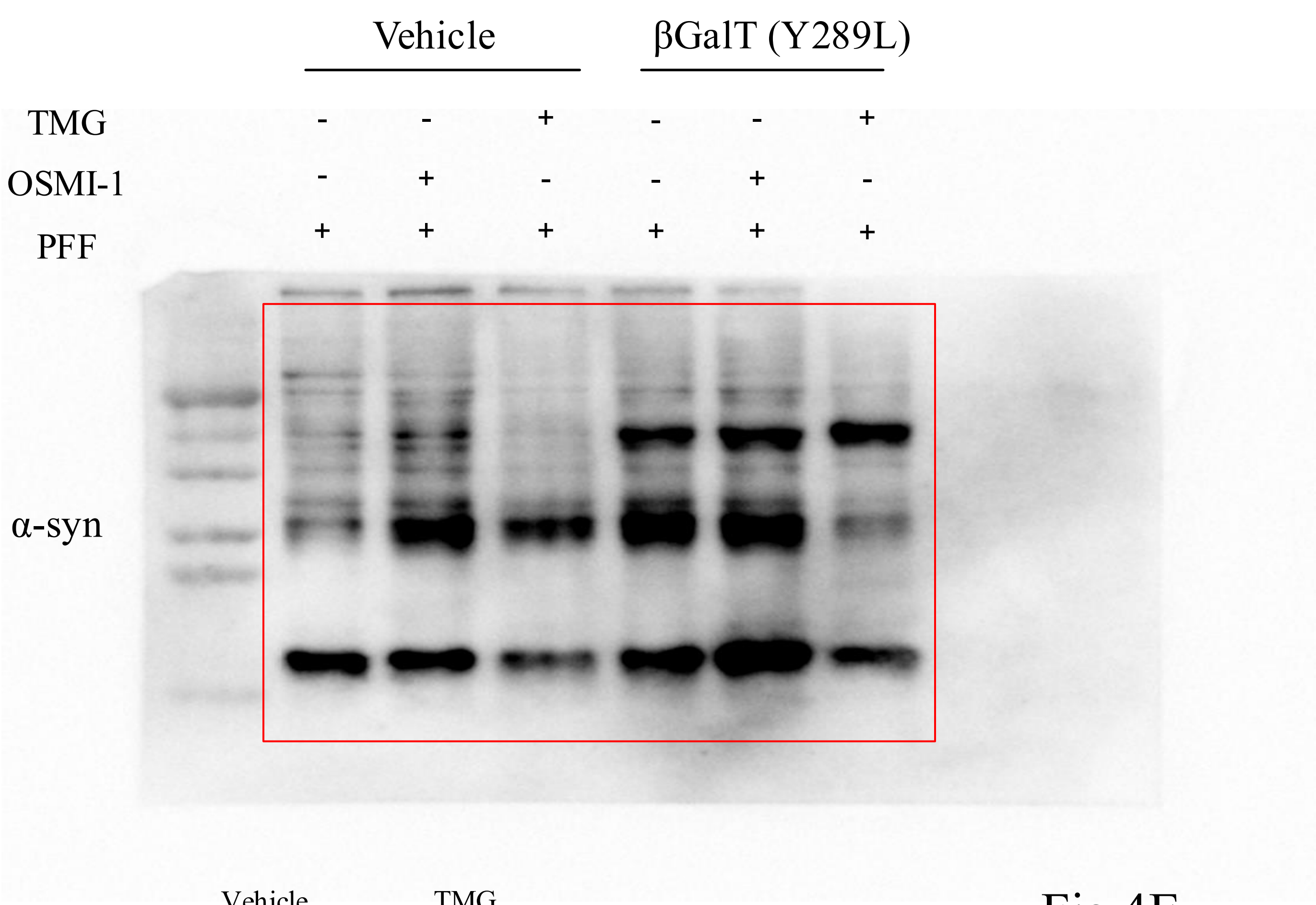


Fig 4D

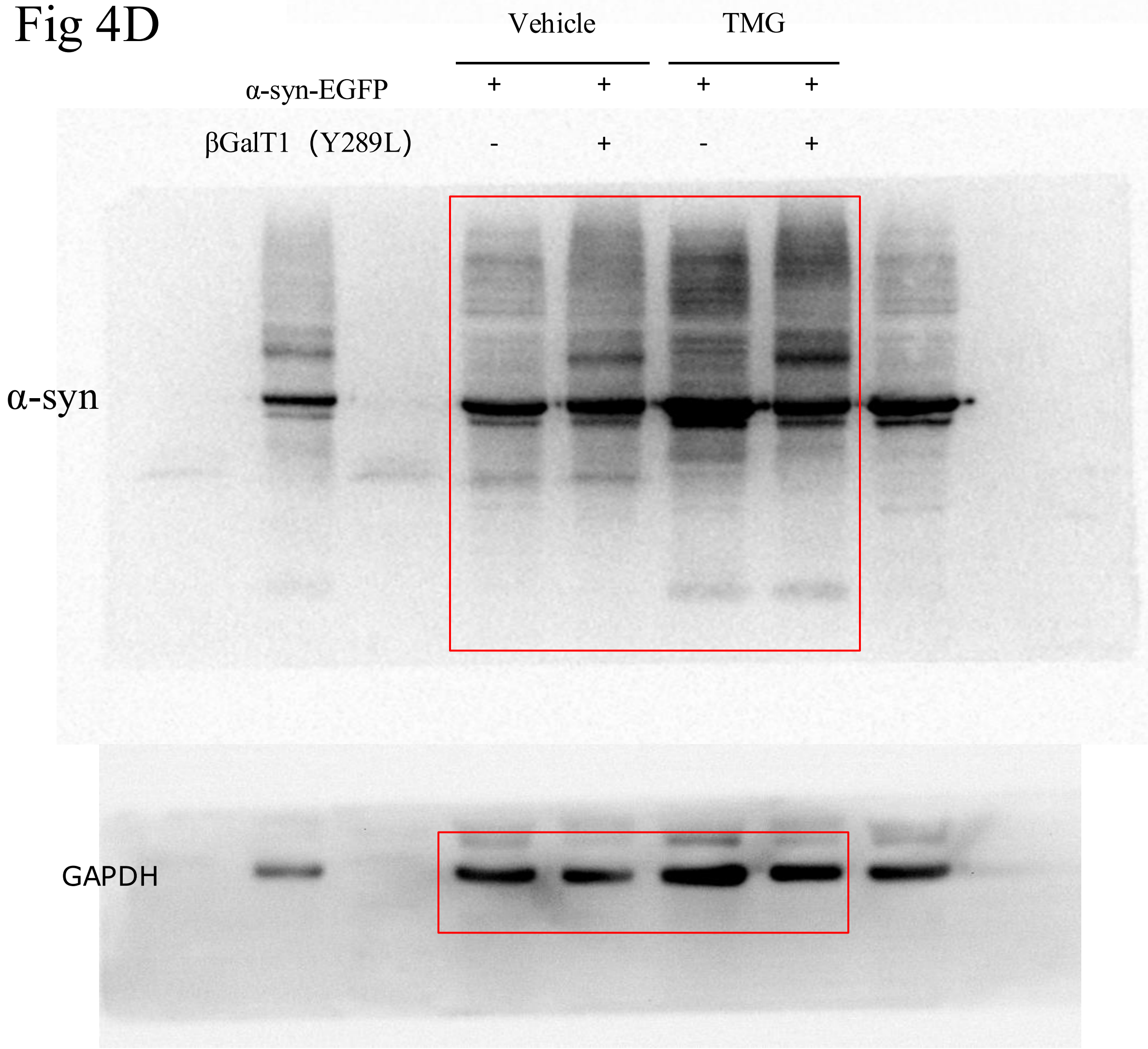


Fig 4E

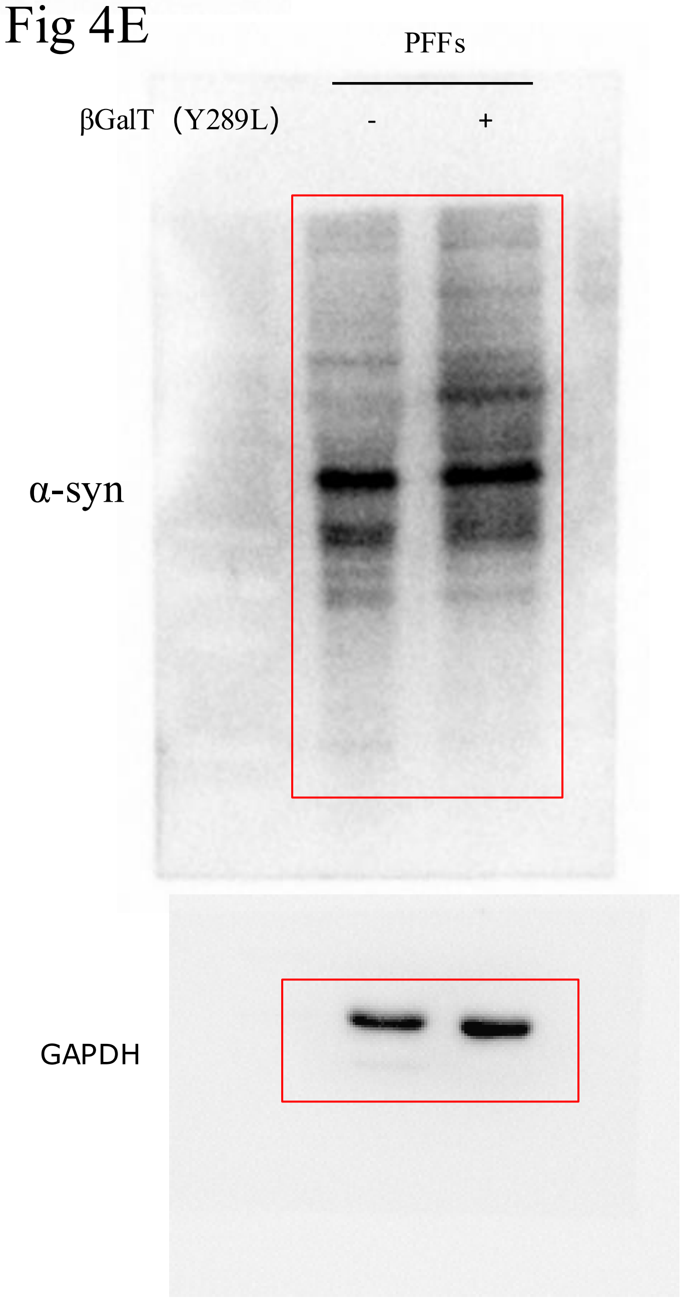

Fig 4G

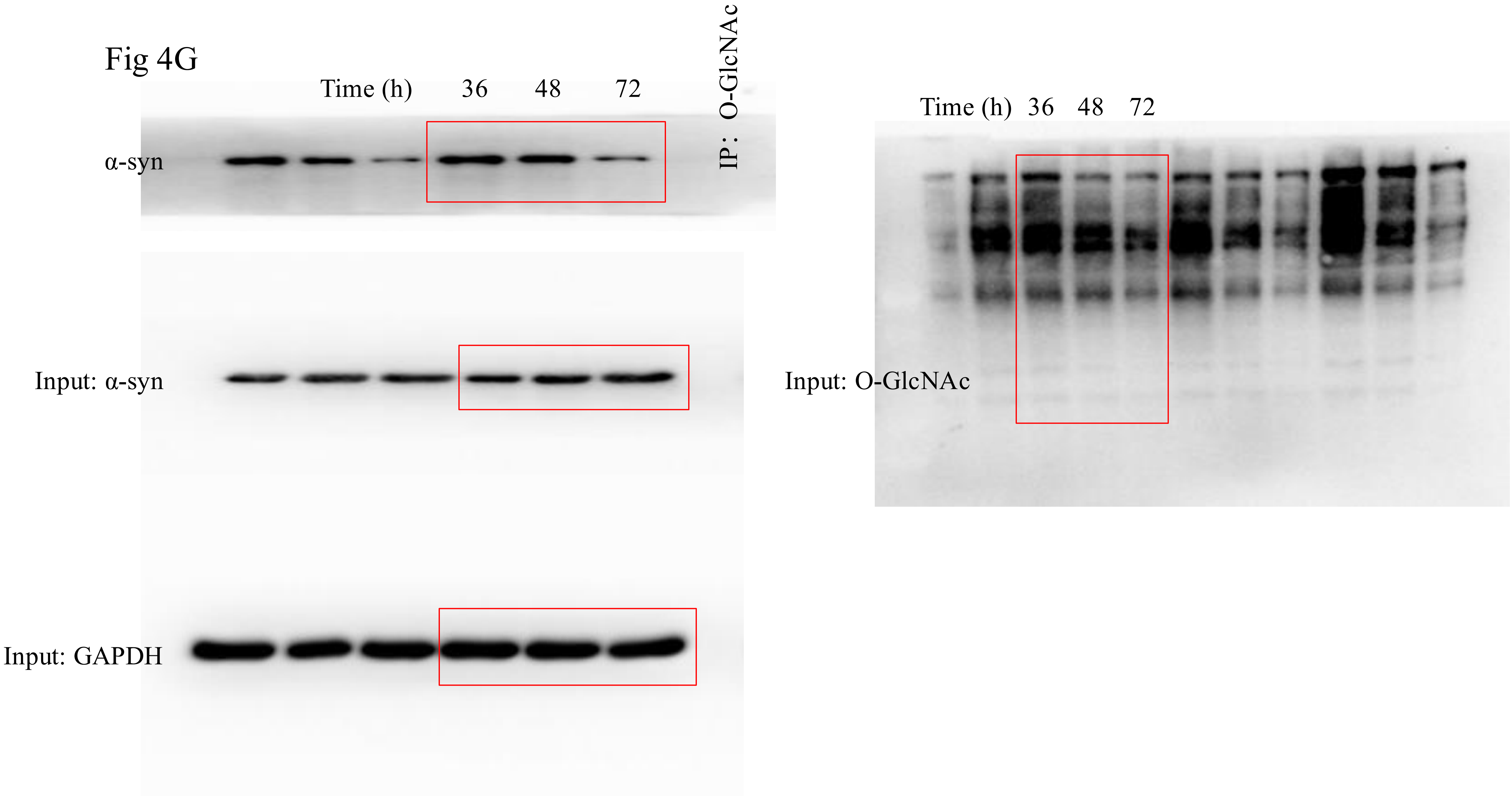
Time (h)
36
48
72
α-syn
IP : O-GlcNAc
Input: α-syn
Input: GAPDH
Time (h) 36 48 72
Input: O-GlcNAc

Fig 4H

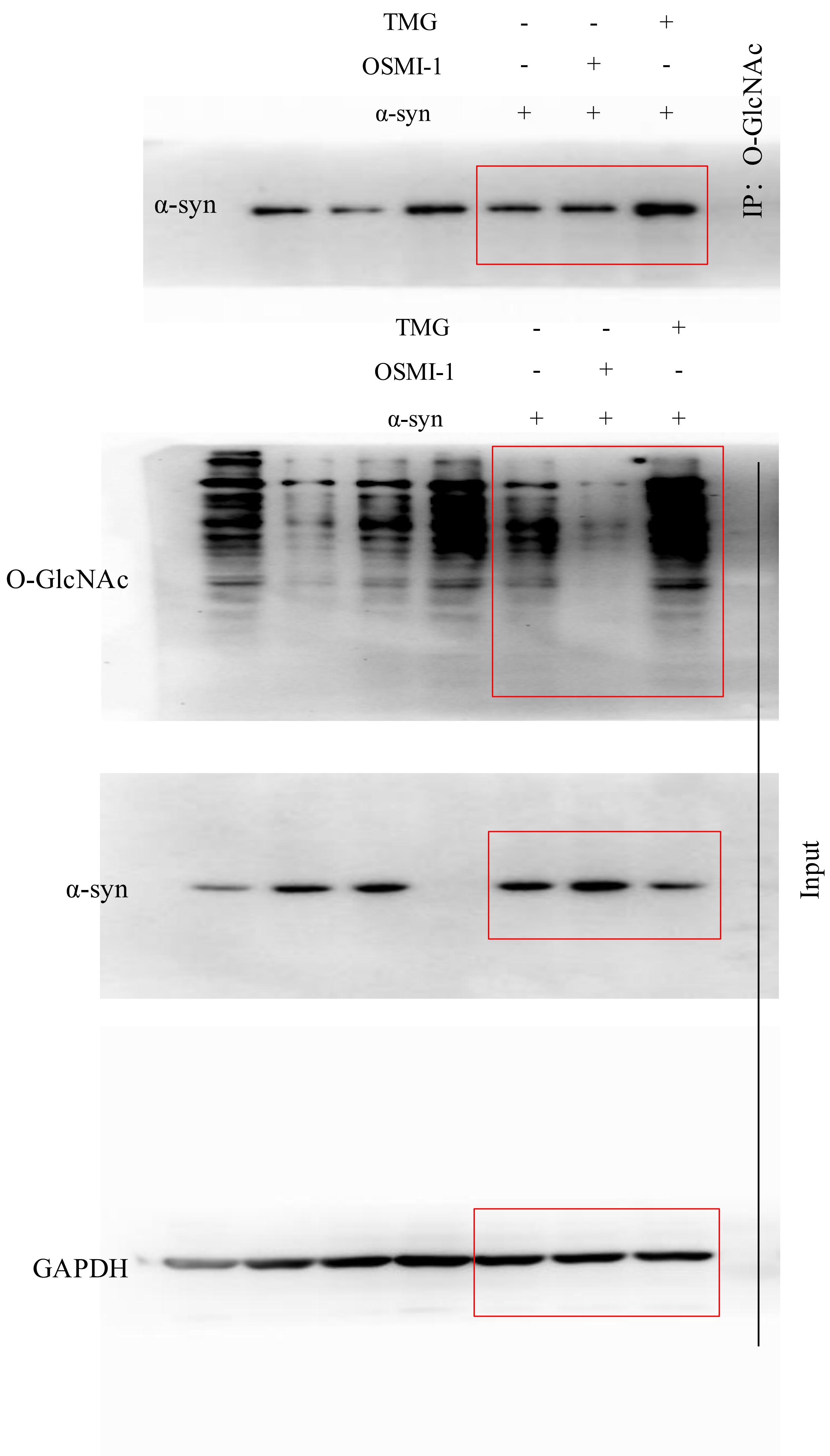

TMG - - +
OSMI-1 - + -
α-syn + + +
α-syn
IP: O-GlcNAc
TMG - - +
OSMI-1 - + -
α-syn + + +
O-GlcNAc
α-syn
GAPDH
Input


Fig 4I

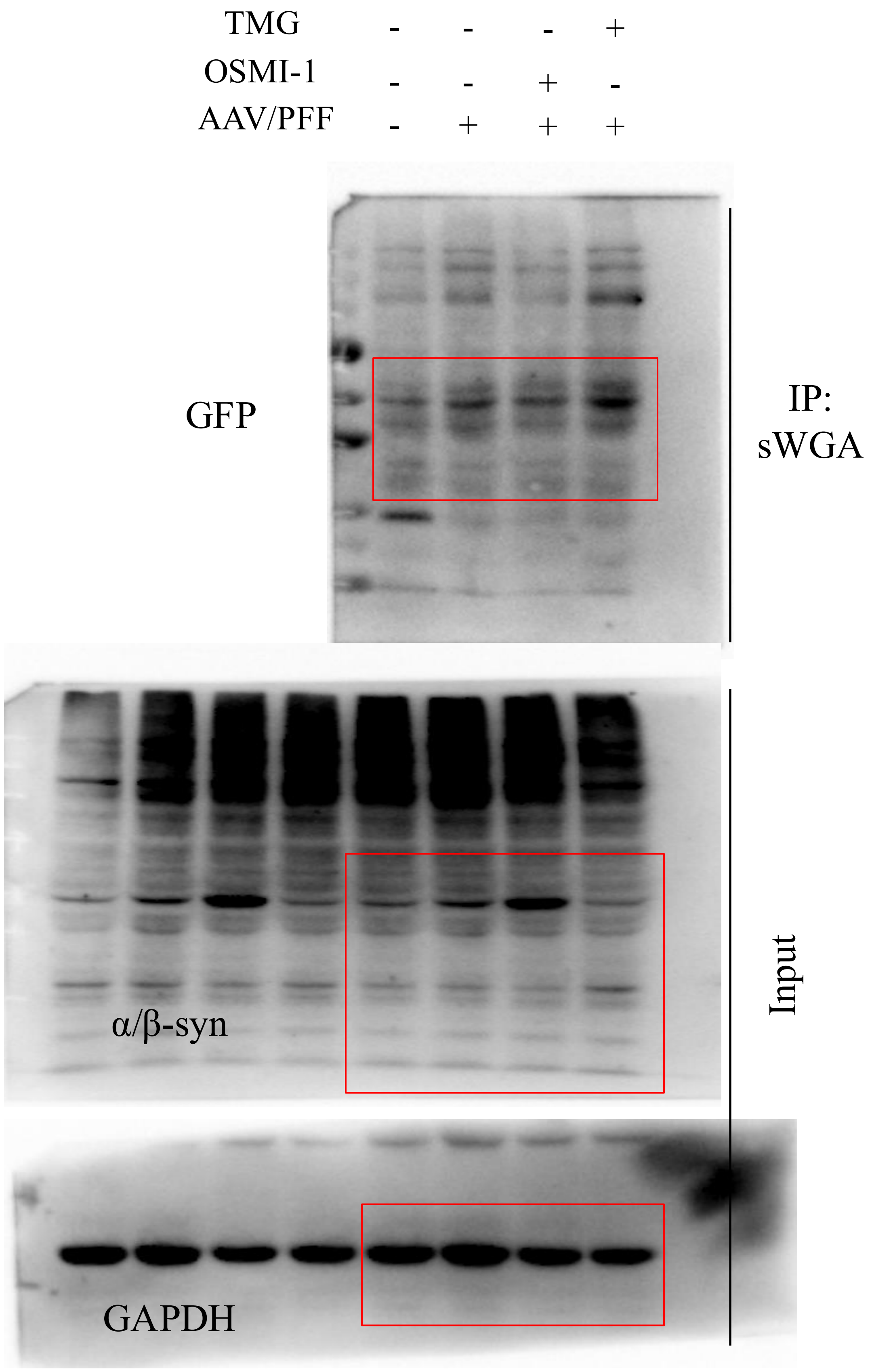

TMG - - - +
OSMI-1 - - + -
AAV/PFF - + + +
GFP
IP: sWGA
α/β-syn
GAPDH
Input

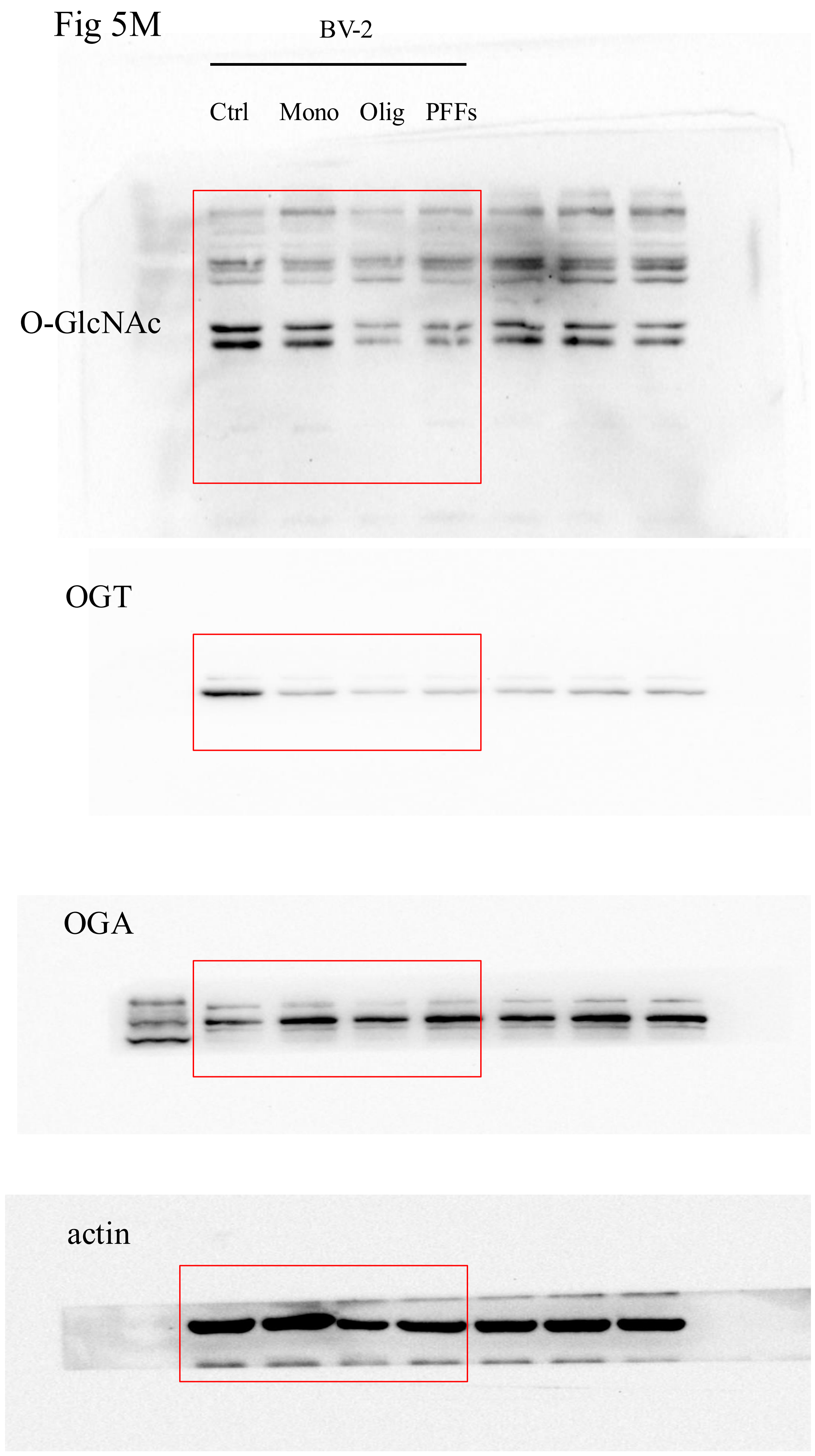
Fig 5M
BV-2
Ctrl
Mono
Olig
PFFs
O-GlcNAc
OGT
OGA
actin

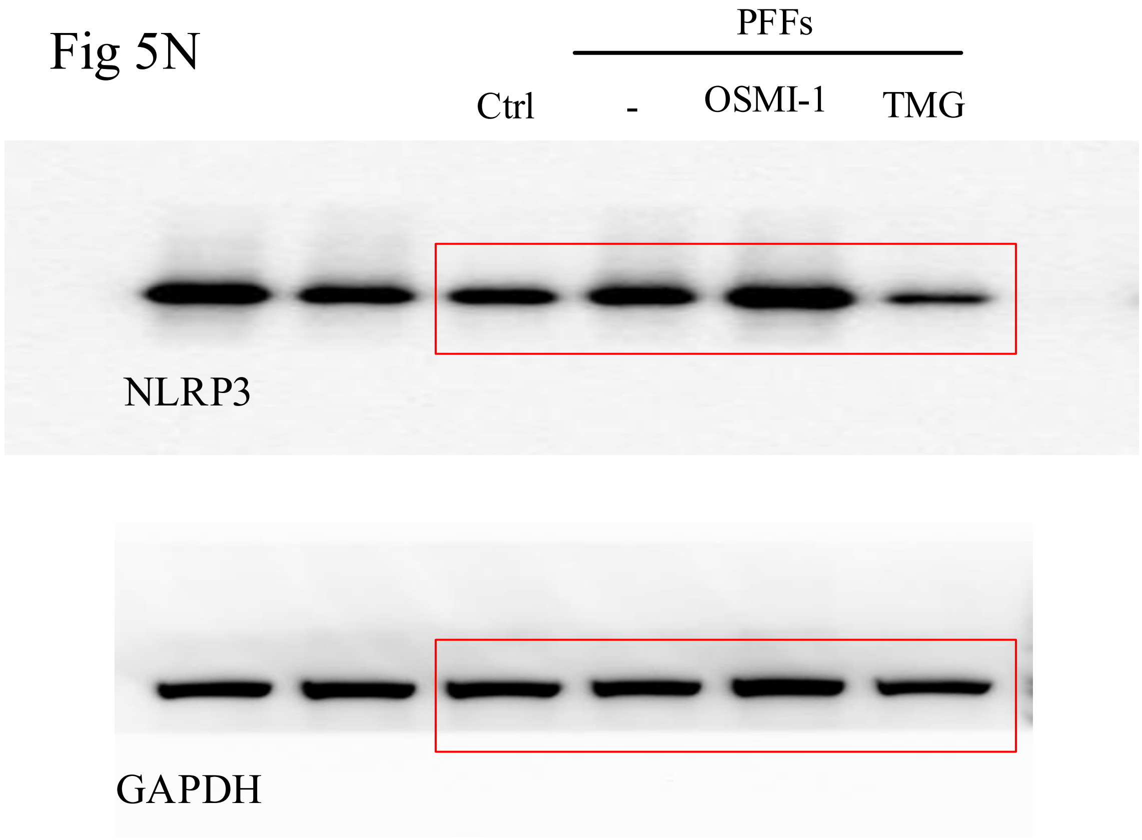
Fig 5N
PFFs
Ctrl
-
OSMI-1
TMG
NLRP3
GAPDH

Fig 6A

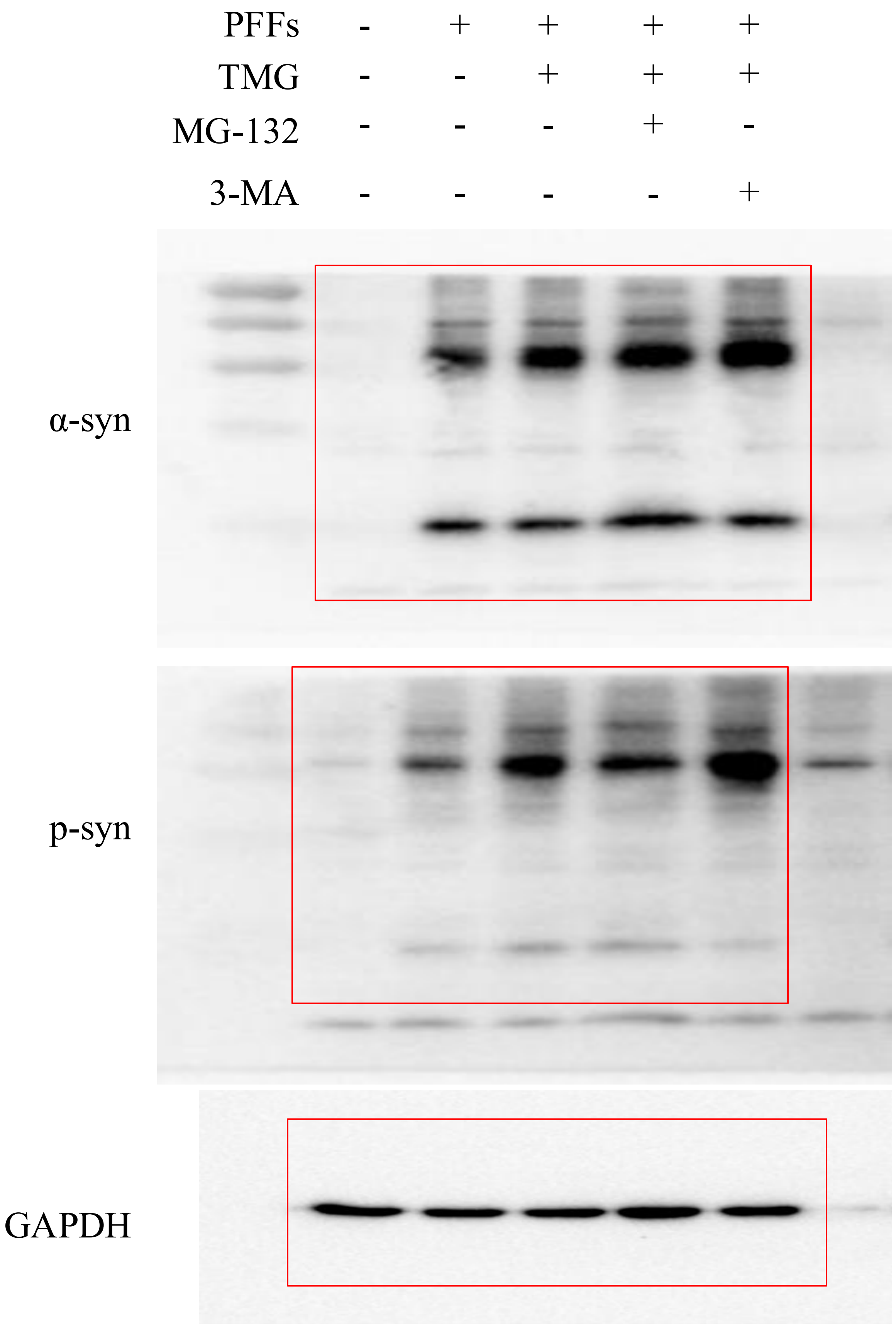

Fig 6K

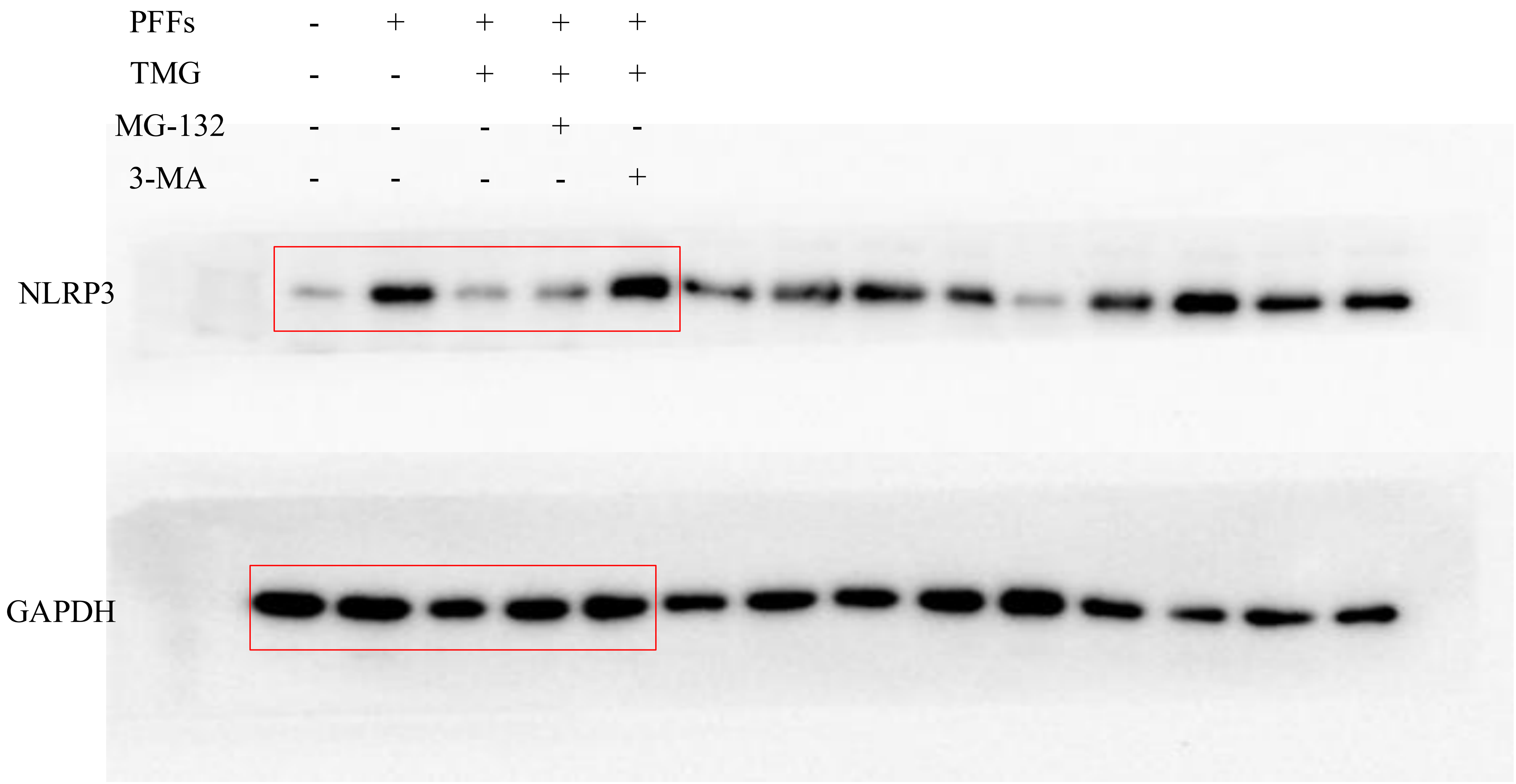

Fig S1A

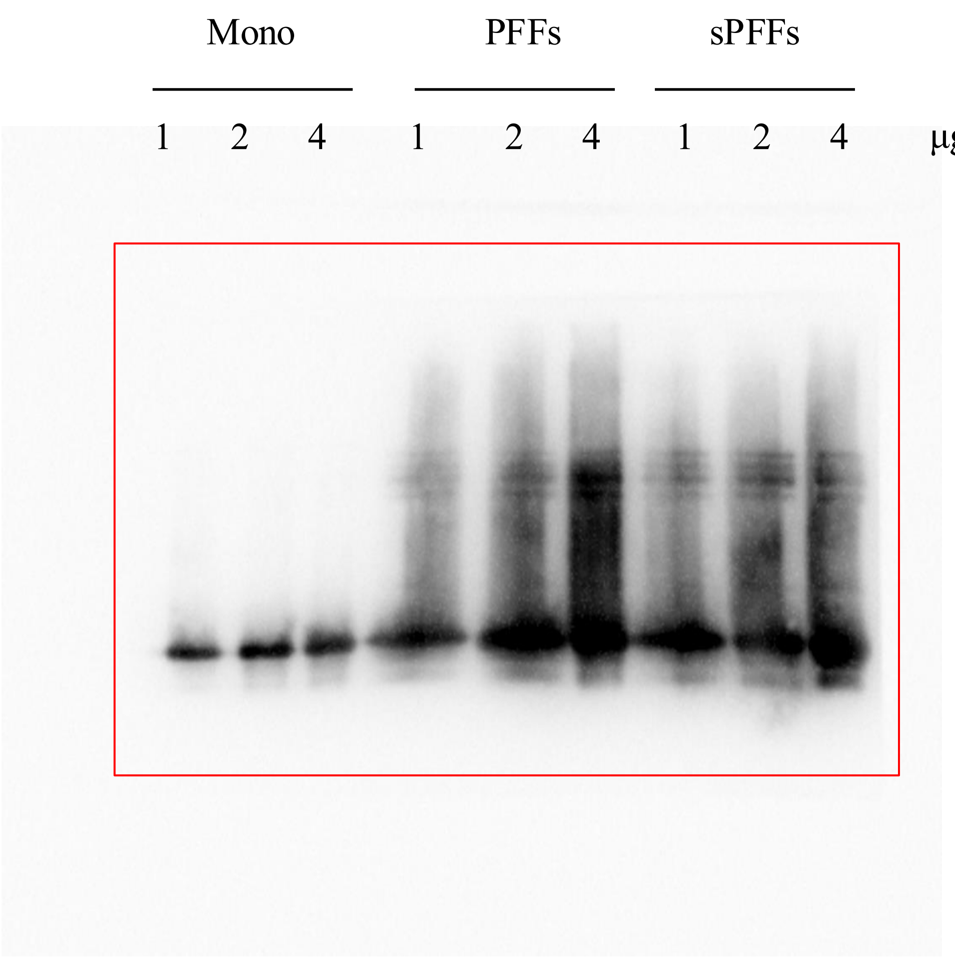


Fig S1B

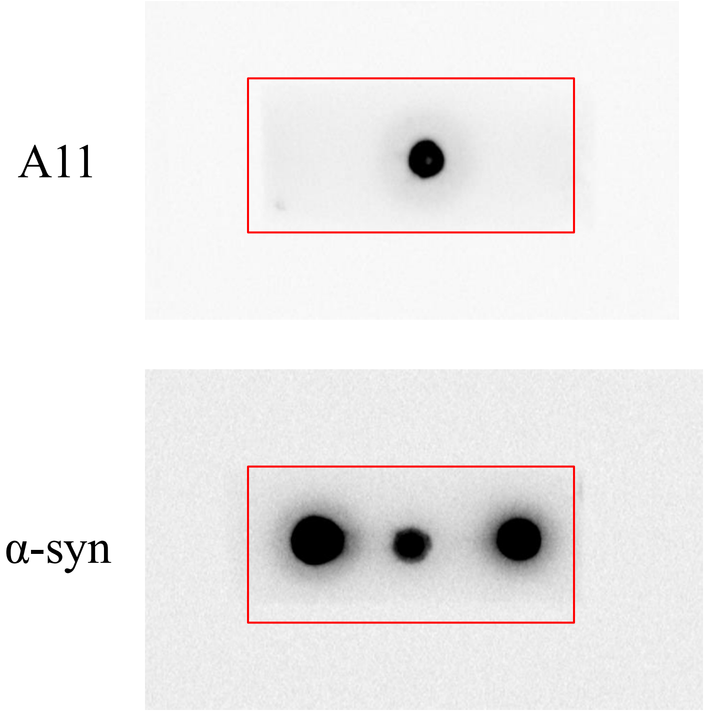


Fig S1E

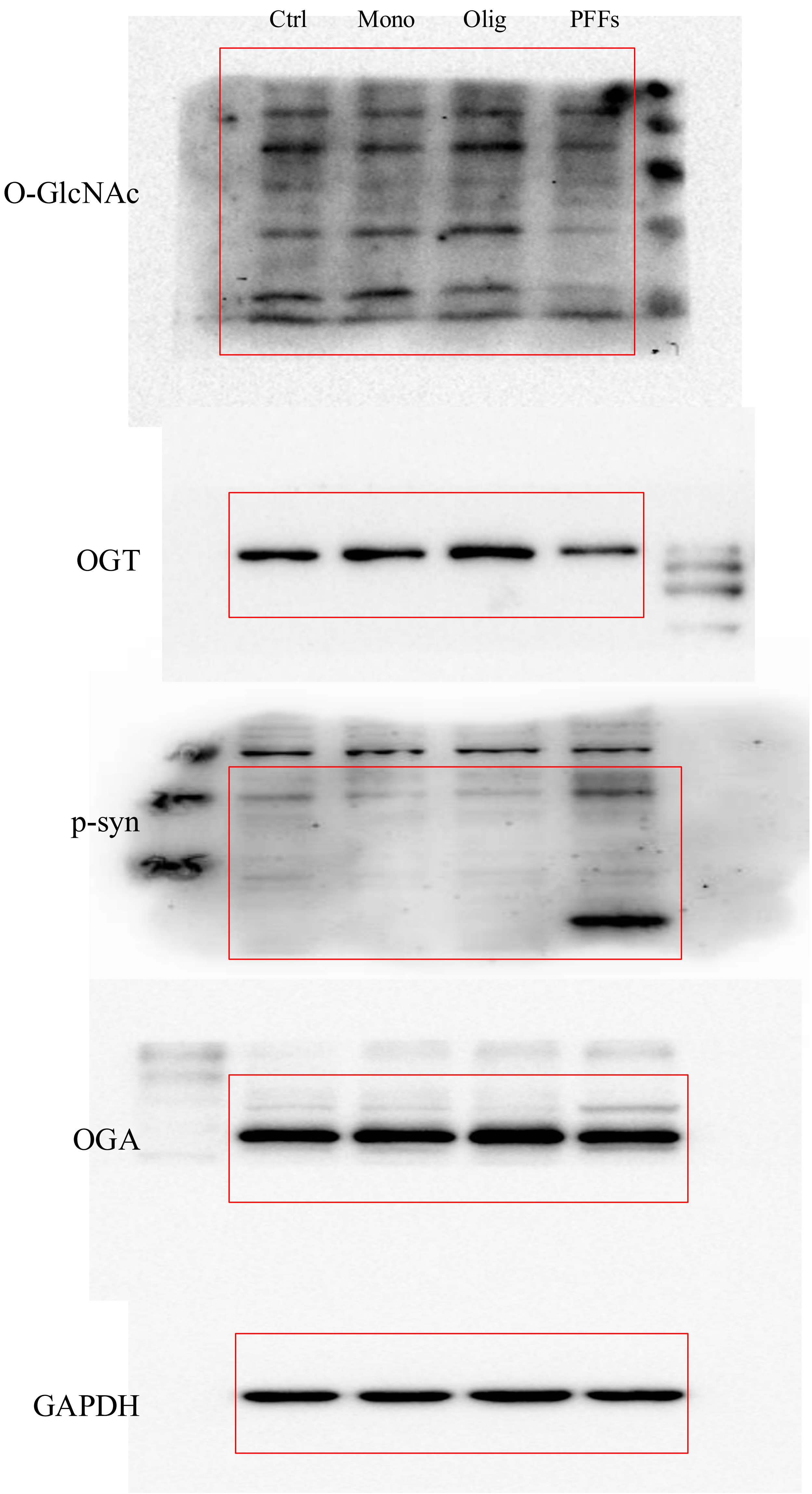

Fig S1F

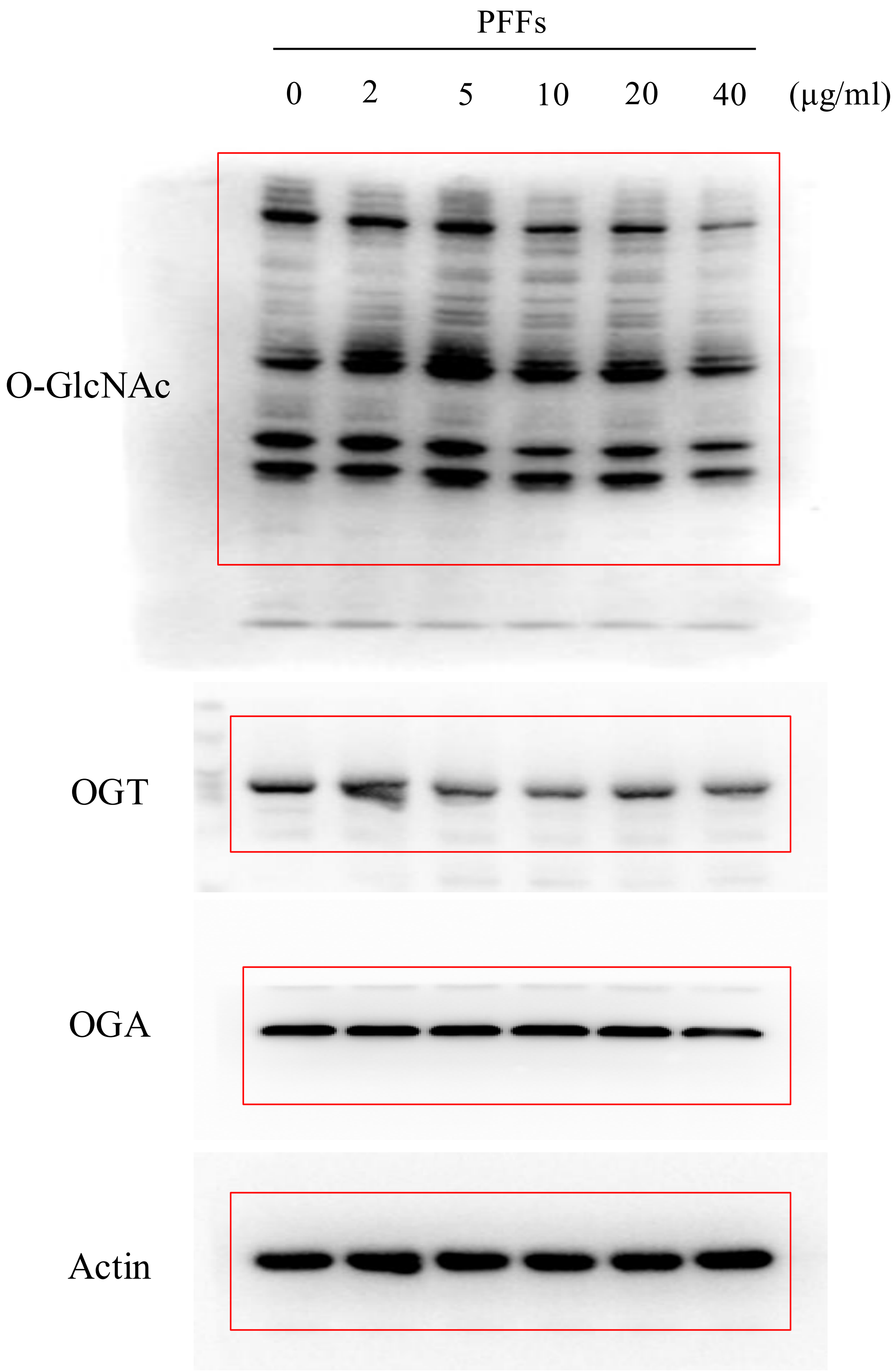

Fig S2B

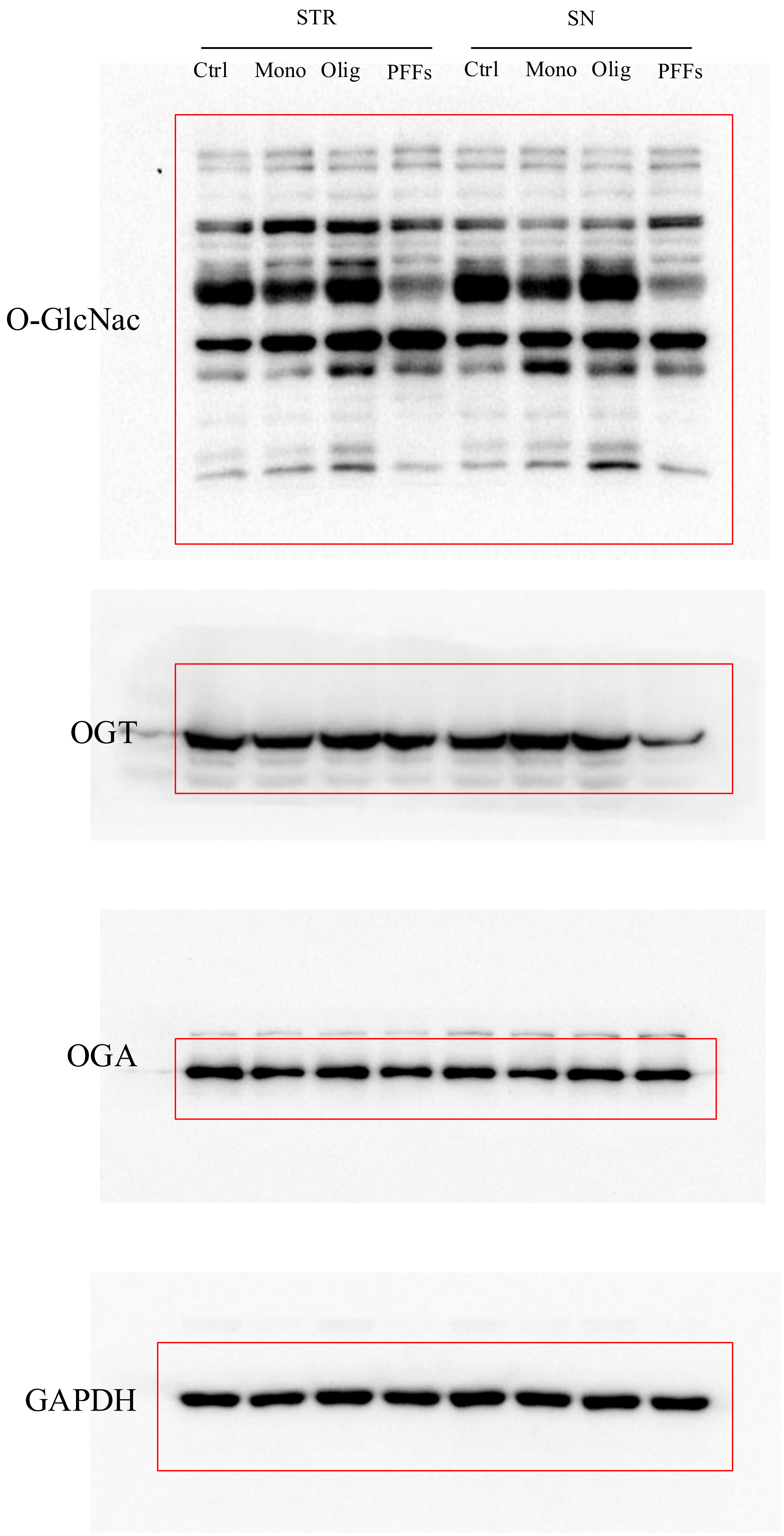


Fig S2E

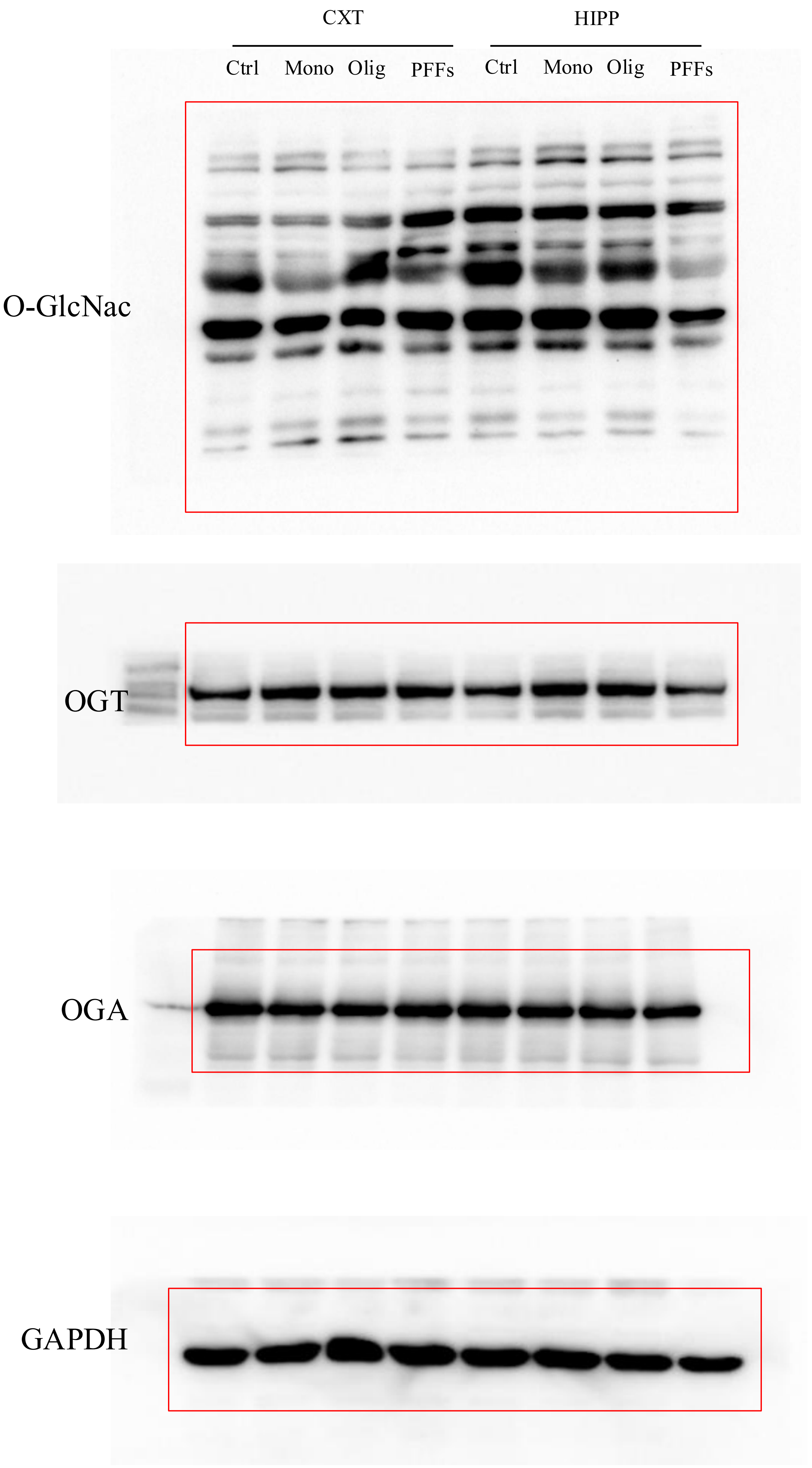

Fig S3D

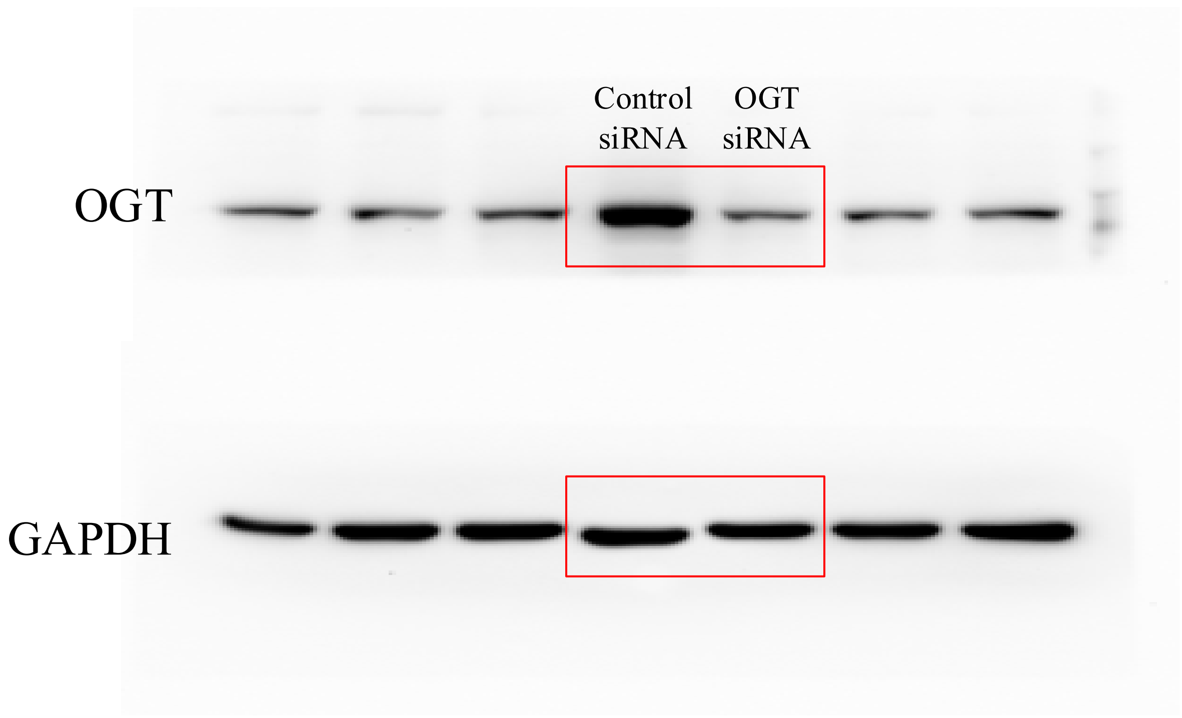


Fig S3F

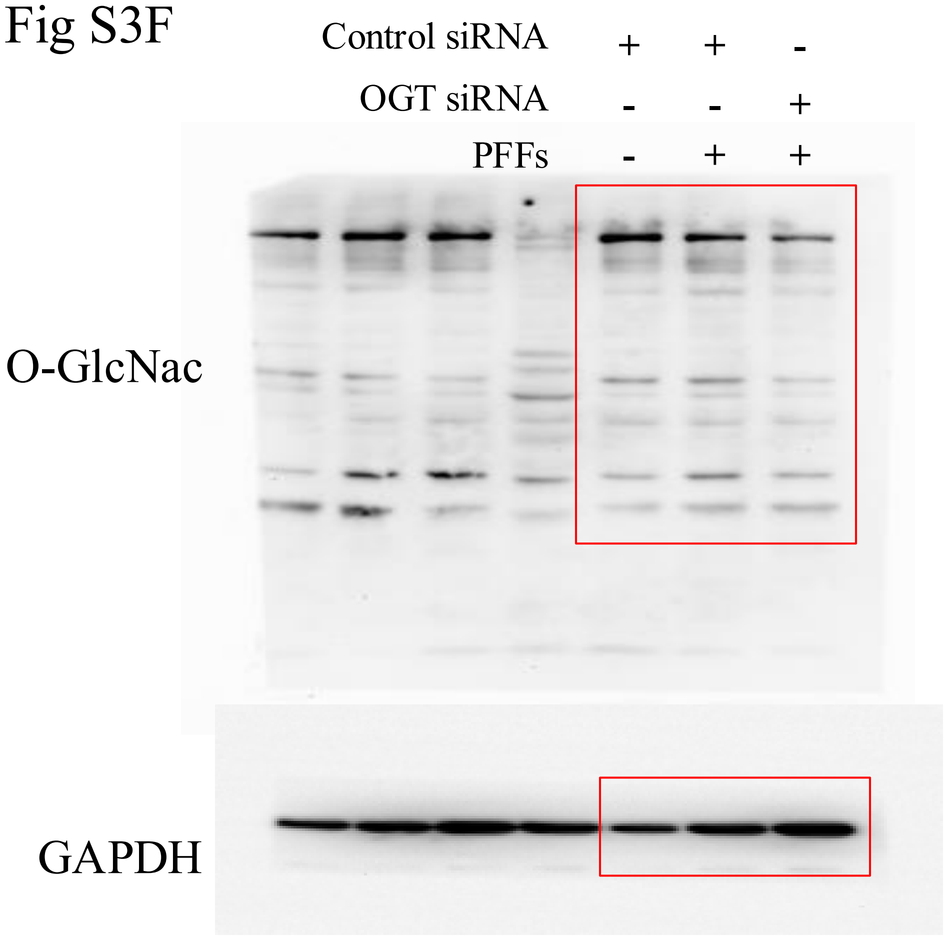

Fig S5D

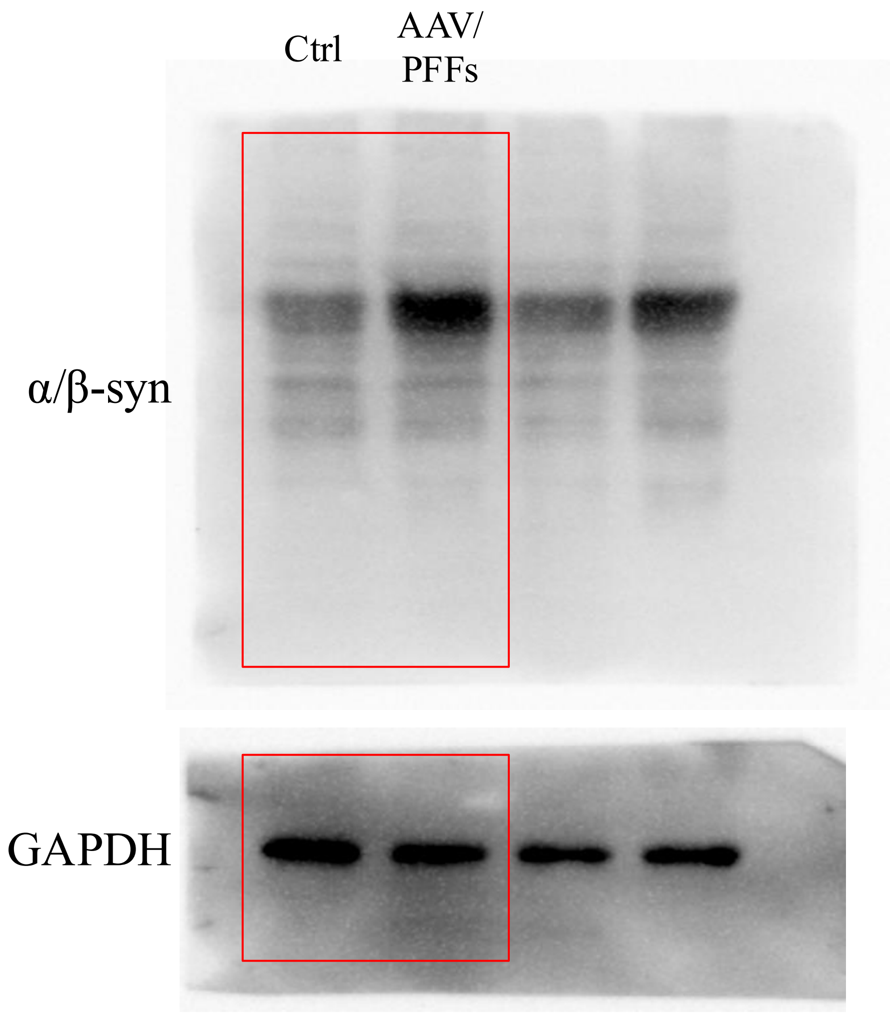

Fig S8A

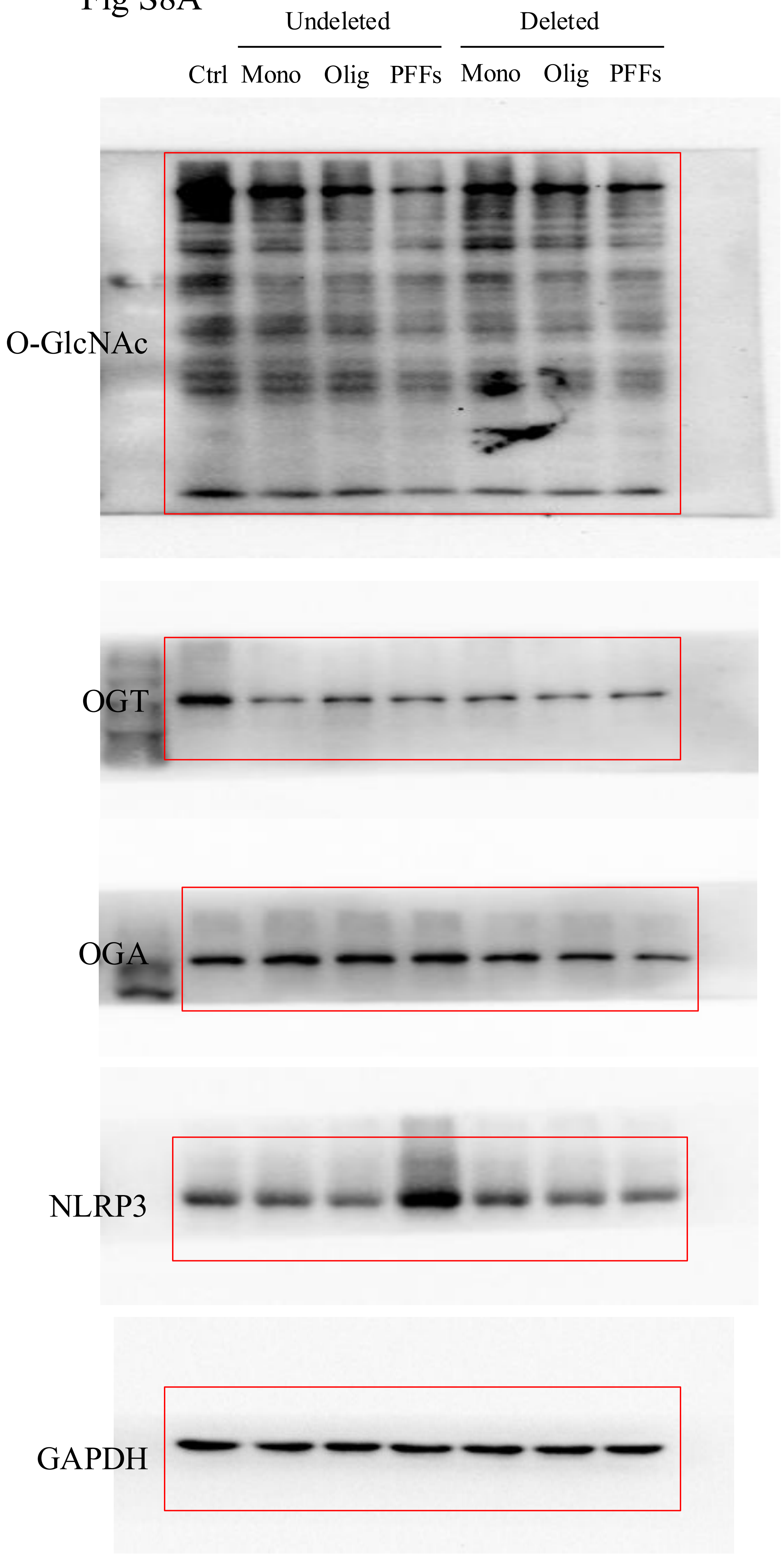


Fig S8F

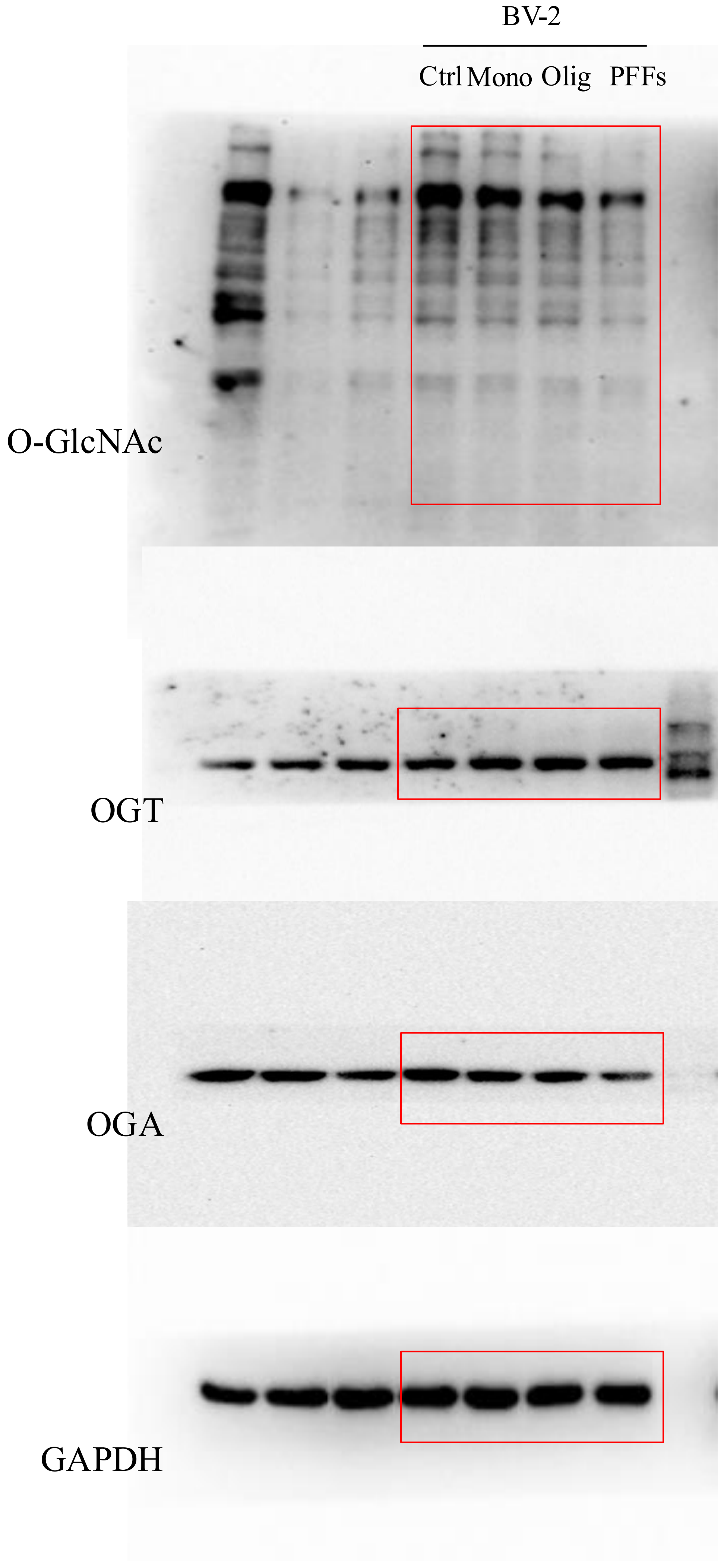

Fig S8J

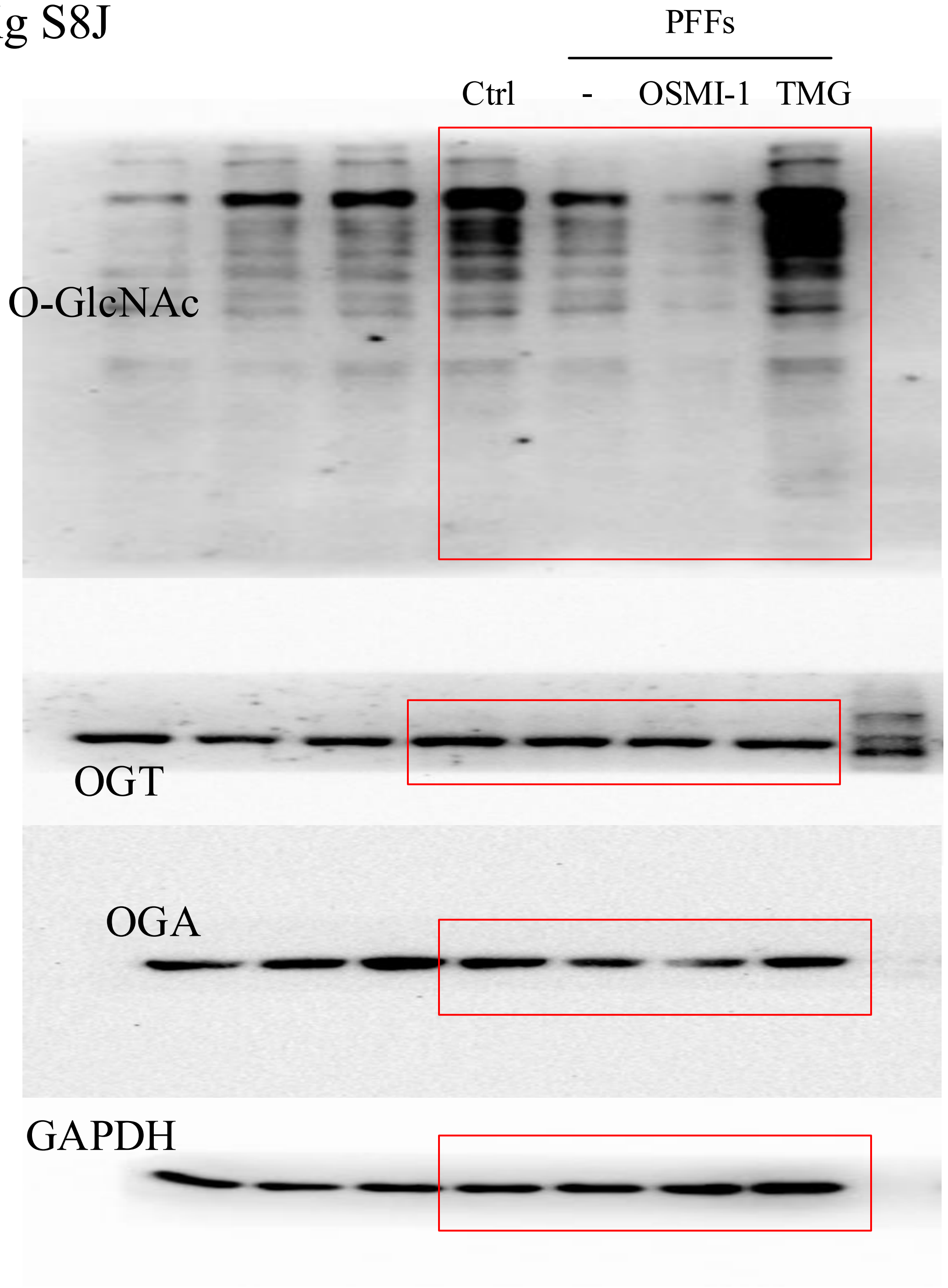


Fig S9B

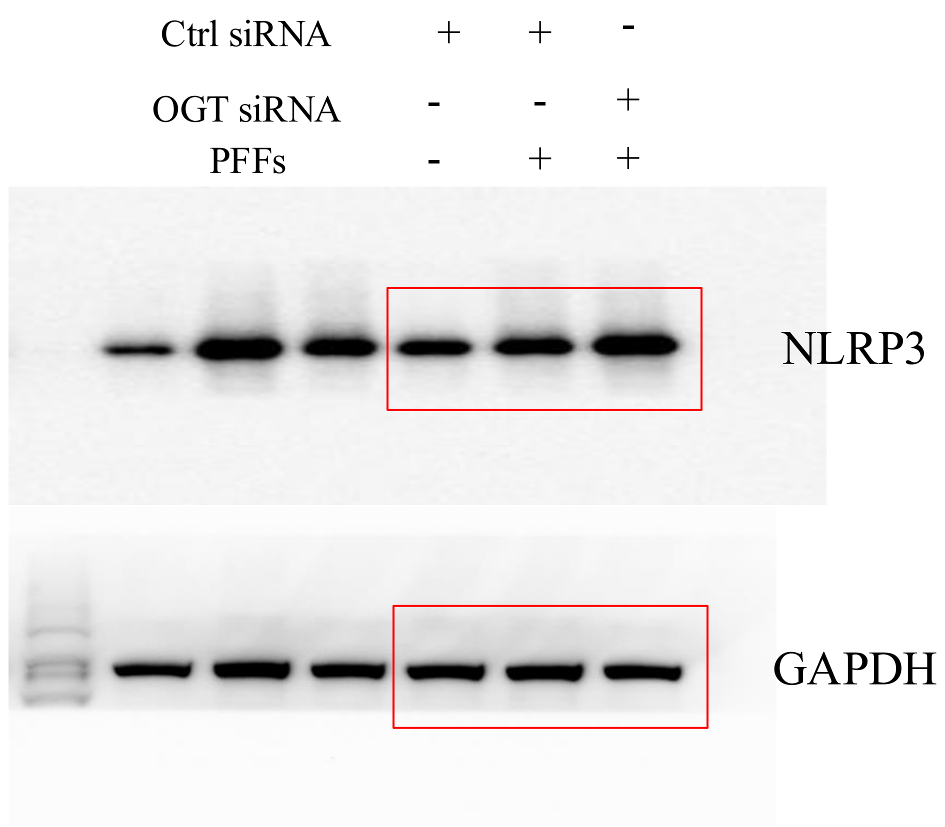


Fig S9D

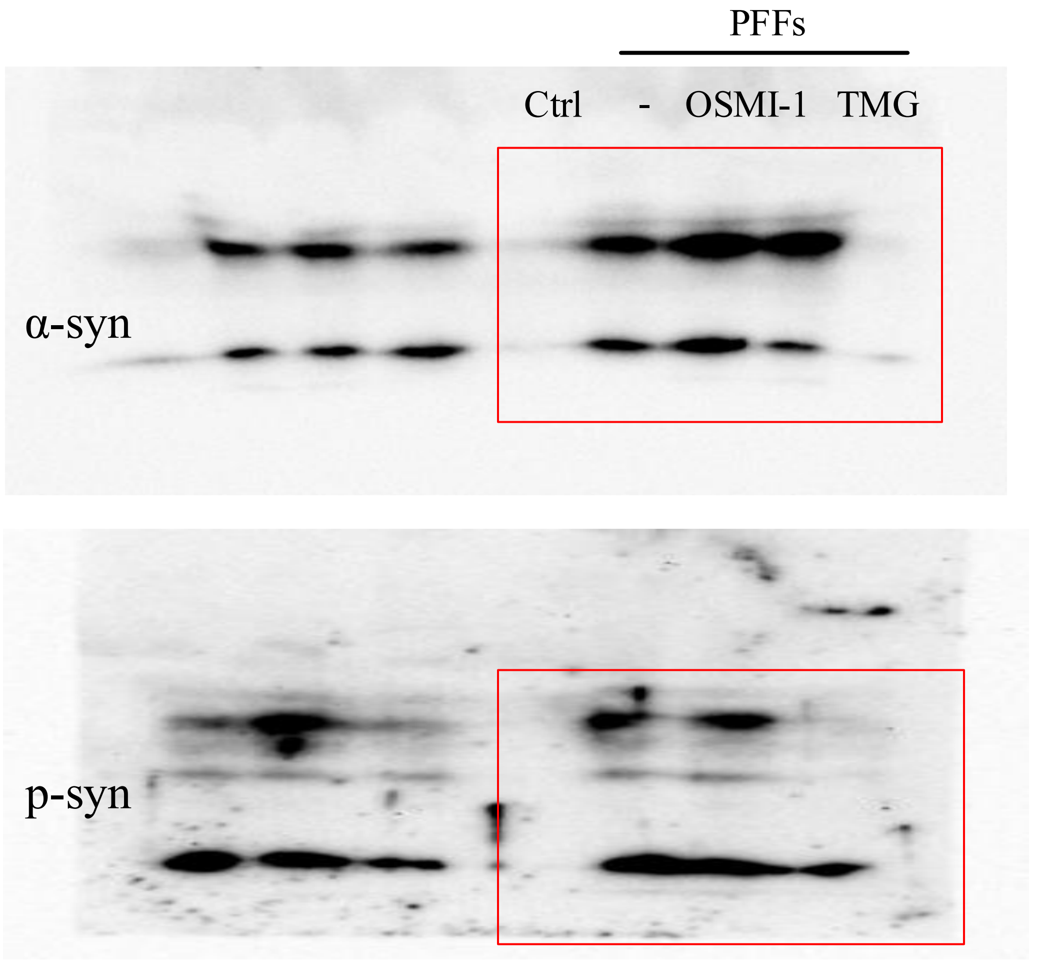


Fig S8L

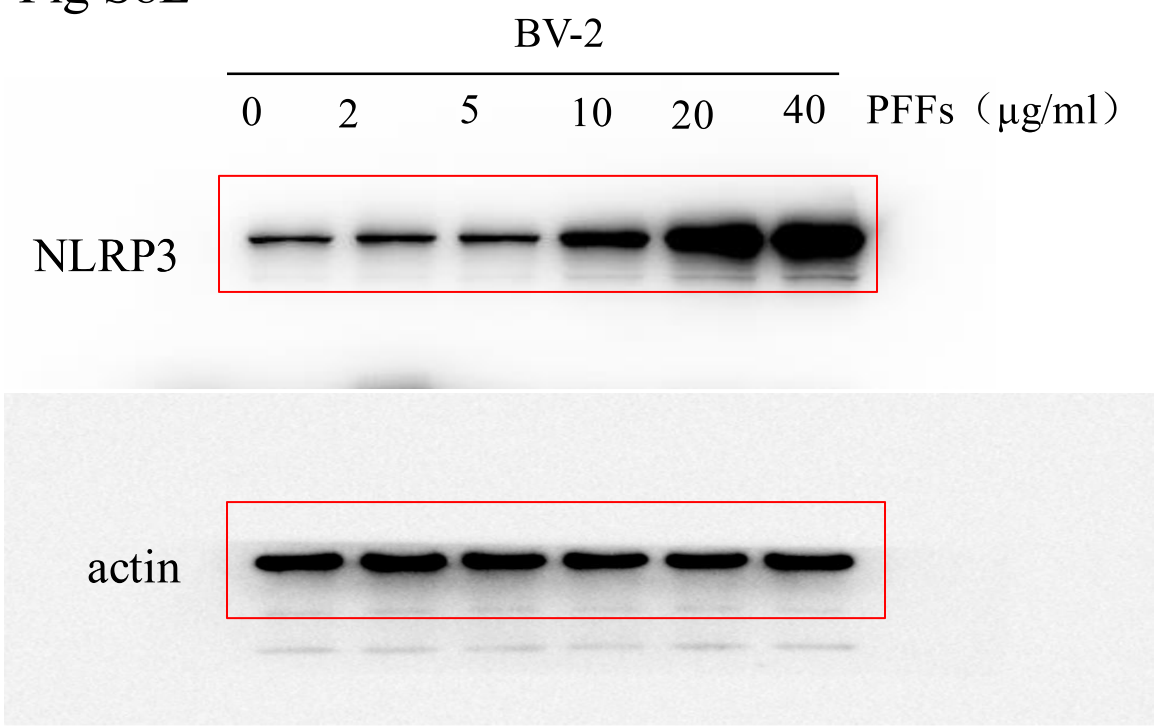

Fig S10E

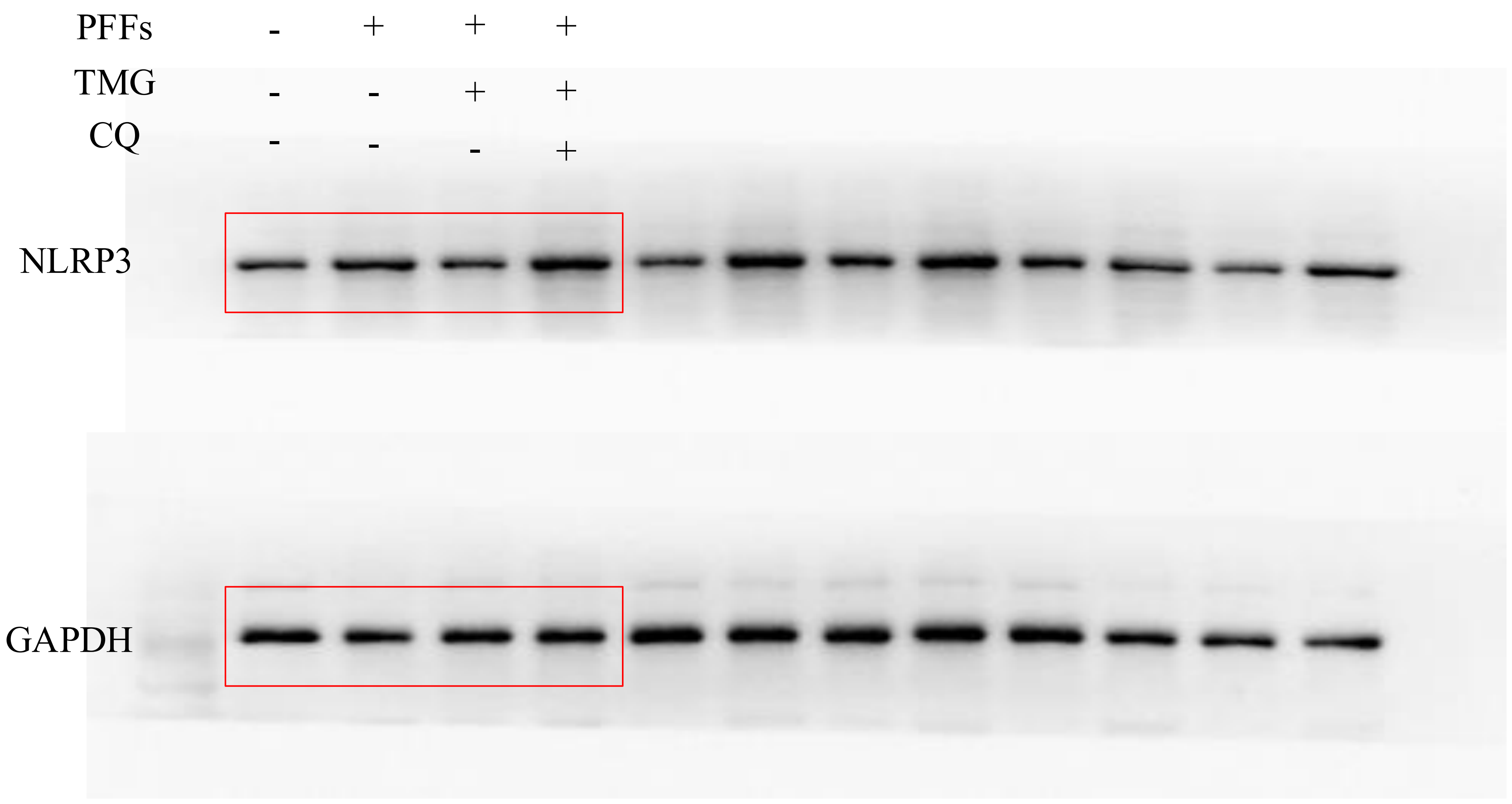

Fig S11A

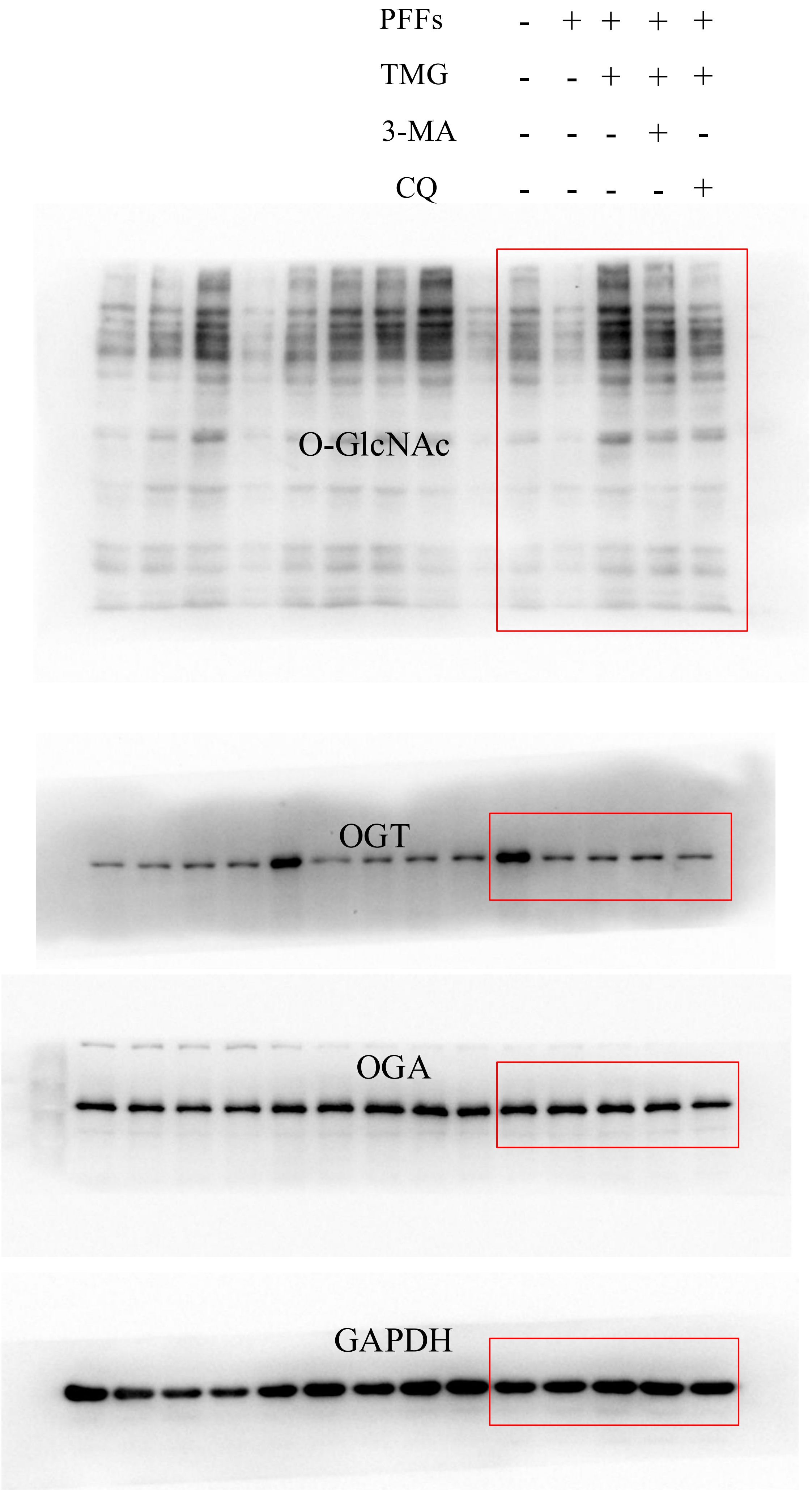


Fig S11B

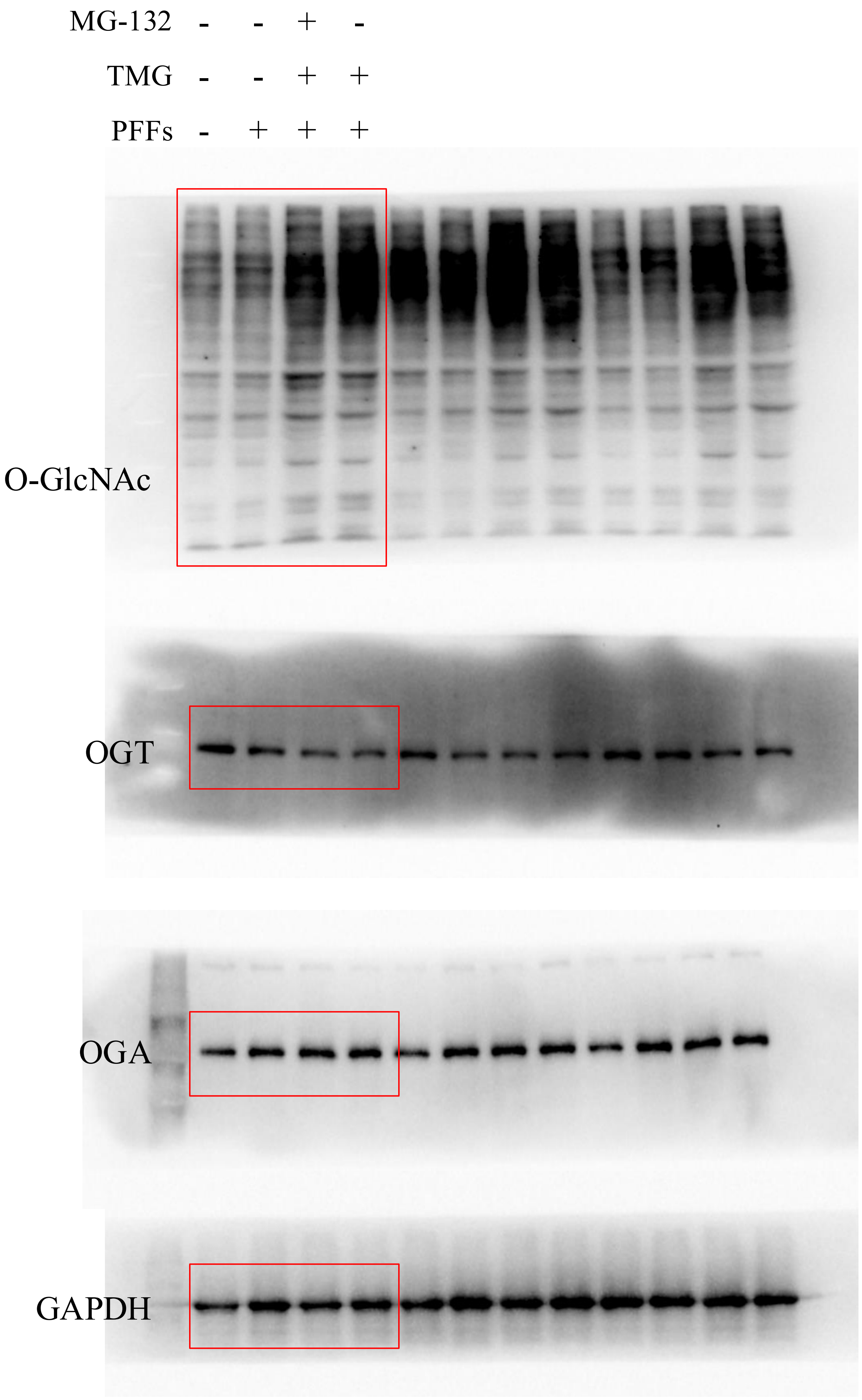

Supplement: Supplementary file 13 — Supplementary Material 13 [file 13024_2025_904_MOESM13_ESM.pdf]
