# Supplementary material for: Modulation of O-GlcNAc cycling influences α-synuclein amplification, degradation, and associated neuroinflammatory pathology
Source: Mol Neurodegener. 2025 Oct 27;20:113. doi: 10.1186/s13024-025-00904-2 (PMC12560605; doi:10.1186/s13024-025-00904-2)
Supplement: Supplementary file 14 — Supplementary Material 14 [file 13024_2025_904_MOESM14_ESM.docx]

Supplementary materials

Title: Modulation of O-GlcNAc cycling influences α-synuclein amplification, degradation, and associated neuroinflammatory pathology

**Authors:** Yongzhen Miao^1,2^, Ting Zhang^1^, Zhuoya Ma^1^, Huanhuan Du^1^, Qipei Gu^1^, Mengni Jiang^1^, Kangping Xiong^2^, Chun-feng Liu^1,2^*, Hongrui Meng^1,2^*

^1^Institute of Neuroscience, Soochow University, Suzhou, 215123, Jiangsu, China.

^2^Department of Neurology, Second Affiliated Hospital of Soochow University, Suzhou, 215004, Jiangsu, China.

*Corresponding author: [liuchunfeng@suda.edu.cn](mailto:liuchunfeng@suda.edu.cn); [hrmeng@suda.edu.cn](mailto:hrmeng@suda.edu.cn)

**List of Supplementary Materials**

Figs. S1 to S12

Tables S1 to S3


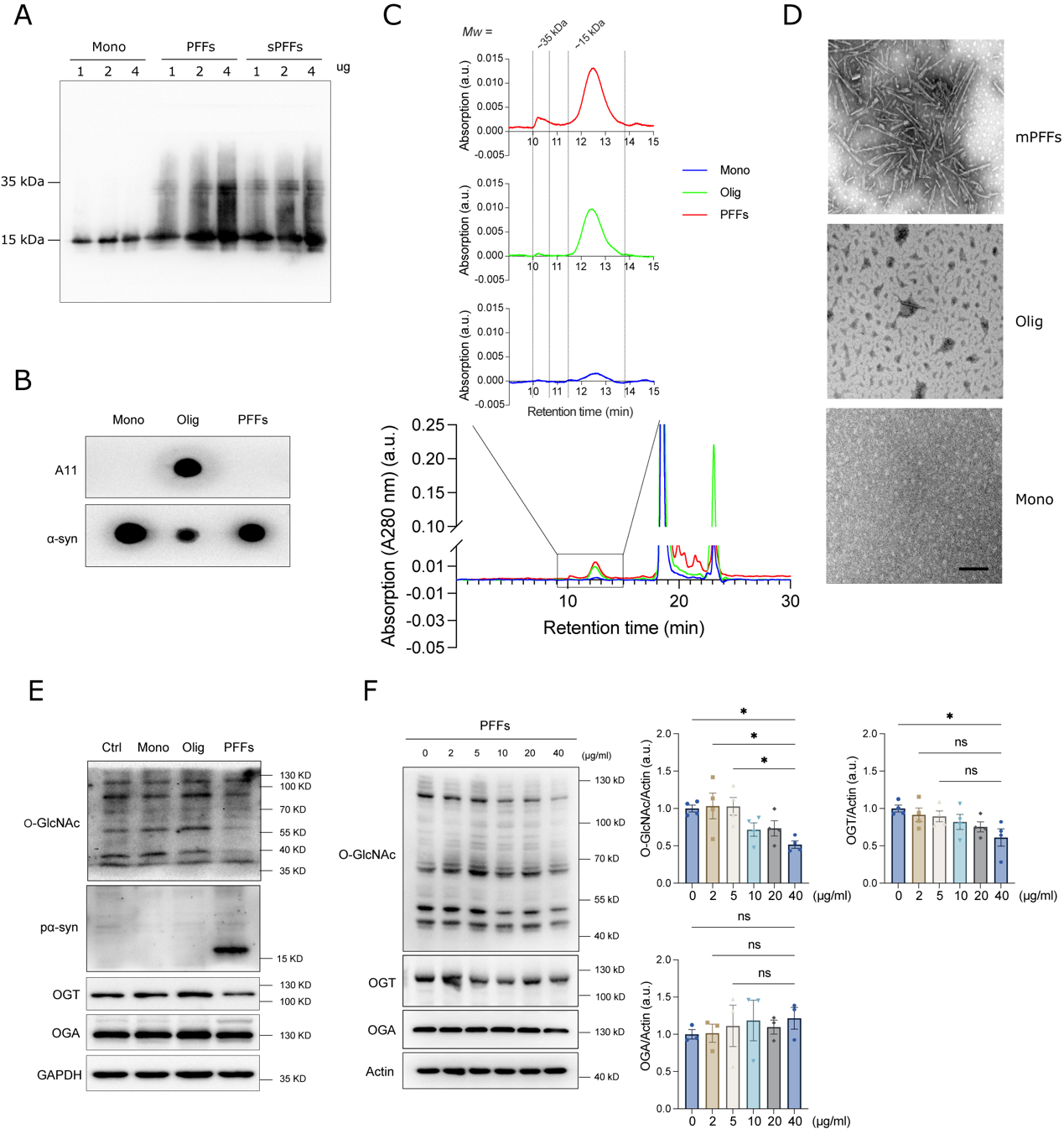


**Fig S1. Conformational strains of α-syn preparation and culture cell treatment**

(**A**) Immunoblot analysis of α-syn monomer and preformed fibrils (PFFs). The monomeric α-syn was obtained by diluting the recombinant proteins in Tris-HCl buffer (pH 7.5), following the removal of pre-existing aggregated species through ultracentrifugation and filtration. The oligomer and PFFs were obtained by incubating 5 mg/mL of monomeric proteins (see Materials and Methods). Freshly purified α-syn monomer and PFFs fragmented by sonication (sPFFs) were used in the experiments. (**B**) Dot blot analysis of α-syn oligomers with the oligomer antibody A11, which specifically recognizes the amyloid β-sheets. (**C**) HPLC-SEC analysis of α-syn monomer, oligomer, and PFFs. Faster-eluting peaks corresponding to molecular weights of ~35 kDa were detectable in the PFFs aggregation reaction but completely absent in the monomeric α-syn reaction. The slower-eluting peaks corresponding to ~15 kDa existed in all three fractions. (**D**) Transmission electron microscopy (TEM) images of conformational strains of α-syn. Scare bar=100 nm. (**E**) Immunoblot analysis for O-GlcNAc, OGT, and OGA levels in mouse primary neurons cultured for 12 days with conformational strains of α-syn (2 μg/ml) addiction. (**F**) Immunoblot and quantification analysis for O-GlcNAc, OGT, and OGA in SH-SY5Y cells exposed to PFFs at concentrations of 2, 5, 10, 20, and 40 μg/mL for five days. The protein levels were normalized to Actin (n=3). Values are presented as means ± SEM. *P<0.05 and **P<0.01 by one-way ANOVA with Tukey’s post-hoc test.


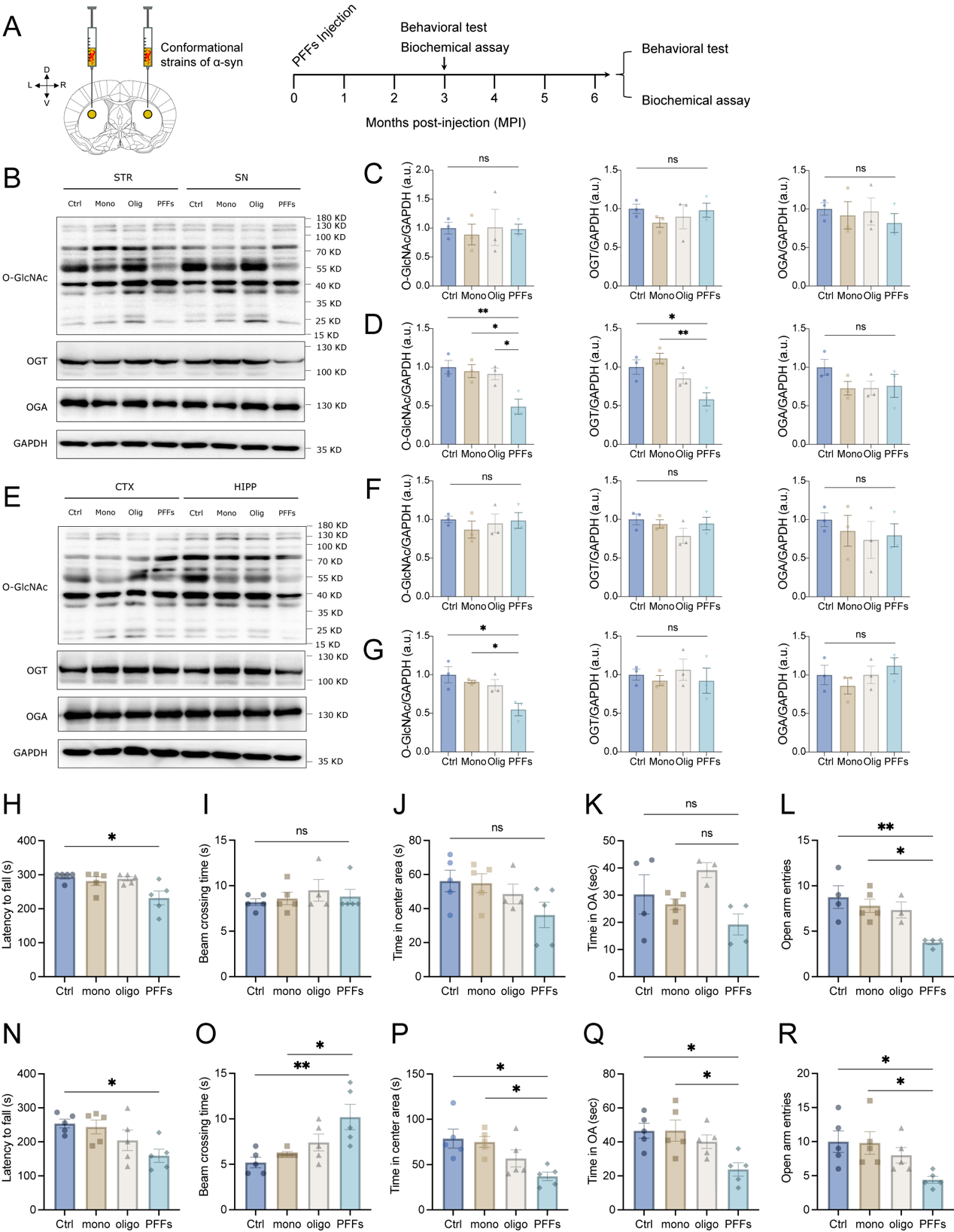


**Fig S2. α-Syn fibril bilateral striatum seeding downregulates O-GlcNAc in connecting regions and causes behavioral impairment**

(**A**) Schematic drawing for the experimental design of α-syn striatum injection. The conformational strains of α-syn (5 ug/side) were bilaterally injected into the striatum. Behavioral tests before brain dissection for biochemical assay following 3- or 6-months post-injection (MPI). (**B**) Representative immunoblot images of O-GlcNAc, OGA, and OGT from the mouse striatum and substantia nigra are displayed. (**C, D**) Densitometric quantification of O-GlcNAc, OGA, and OGT in striatum (C) and substantia nigra (D). The protein levels were normalized to the GAPDH (n=3). (**E**) Representative immunoblot images of O-GlcNAc, OGA, and OGT from the mouse cortex and hippocampus are displayed. (**F, G**) Densitometric quantification of O-GlcNAc, OGA, and OGT proteins in (F) and hippocampus (G). The protein levels were normalized to the expression of GAPDH (n=3). (**H, N**) Rotarod test, (**I, O**) balance beam test, (**J, P**) open field test, and (**K-L, Q-R**) elevated plus maze tests were performed prior to brain dissection following 3- or 6-month post-injection. The corresponding parameter scores were quantified (n=5). Values are presented as means ± SEM. *P<0.05 and **P<0.01by one-way ANOVA with Tukey’s post-hoc test.

**
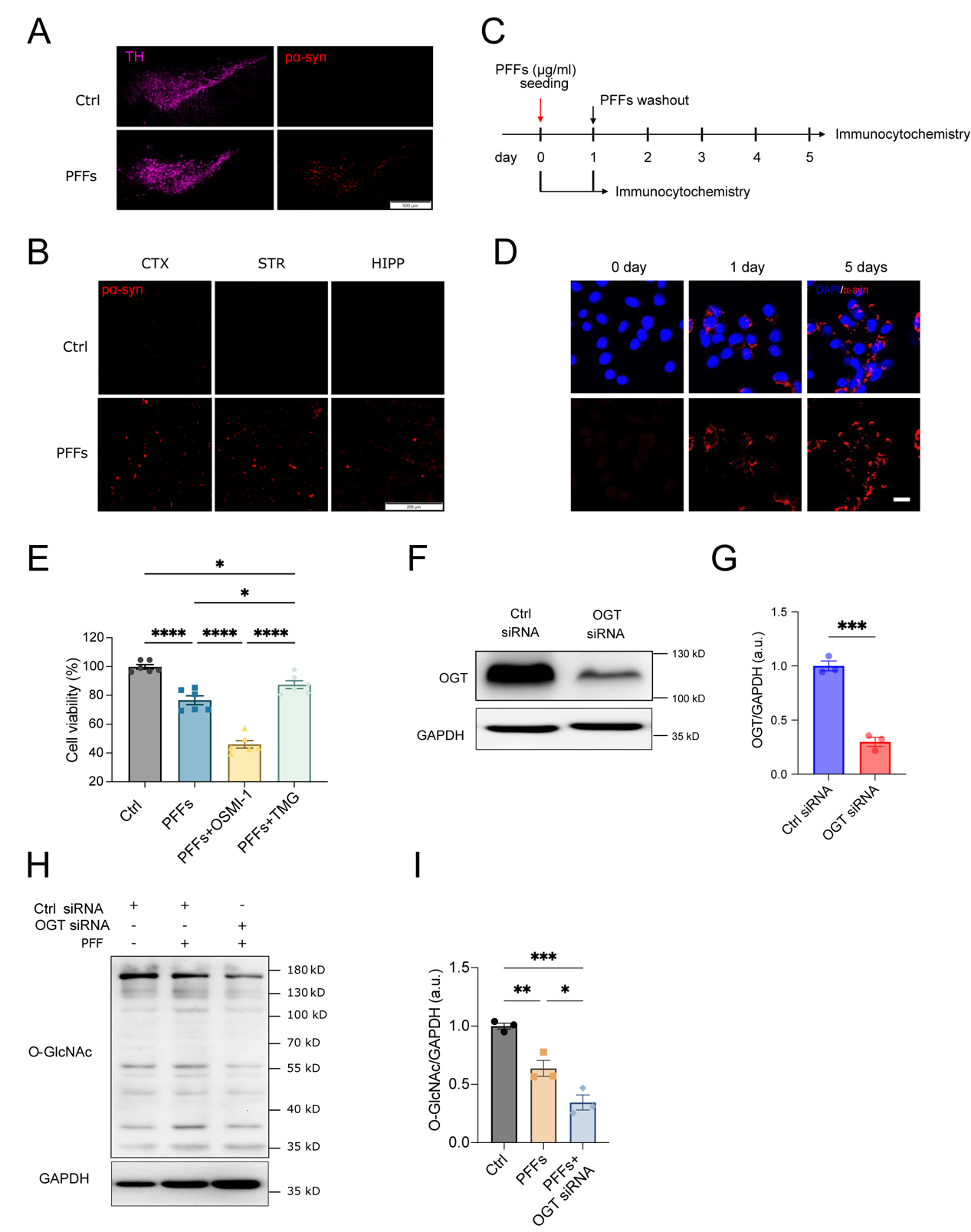
**

**Fig S3. Seeding PFFs causes aggregated α-syn propagation, and modulation of O-GlcNAcylation affects α-syn-induced cell viability**

(**A**) Immunohistochemical staining of pα-syn (red) in dopaminergic neurons (TH^+^, magenta) in the substantia nigra of PFFs-injected mice. Scale bar = 500 μm. (**B**) Staining of pα-syn in the cortex (CTX), striatum (STR), and hippocampus (HIPP) of PFFs-injected mice. Scale bar = 200 μm. (**C**) Schematic overview of the experimental design testing α-syn internalization by SH-SY5Y cells and subsequent aggregation following PFFs seeding. (**D**) Immunocytochemical staining of α-syn aggregates in PFFs-seeded SH-SY5Y cells on days 1 and 5. Scale bar = 10 μm. (**E**) Cell viability of SH-SY5Y cells seeded with PFFs and treated with OSMI-1 or TMG (n=6). (**F-G**) Immunoblot analysis of the OGT knockdown efficiency in 2 μg of *OGT* siRNA-transfected SH-SY5Y cells for 36 hours. A scrambled siRNA was used as a control (Ctrl) for transfection. The OGT levels were normalized to GAPDH (n=3). (**H-I**) Immunoblot and quantification for O-GlcNAc in SH-SY5Y cells transfected with *OGT* siRNA and control. GAPDH serves as a loading control (n=3). Values are presented as means ± SEM. *P<0.05, **P<0.01, ***P<0.001, and ****P<0.0001 by two-tailed Student’s *t*-test (C) or one-way ANOVA with Tukey’s post-hoc test (E, F, and G).


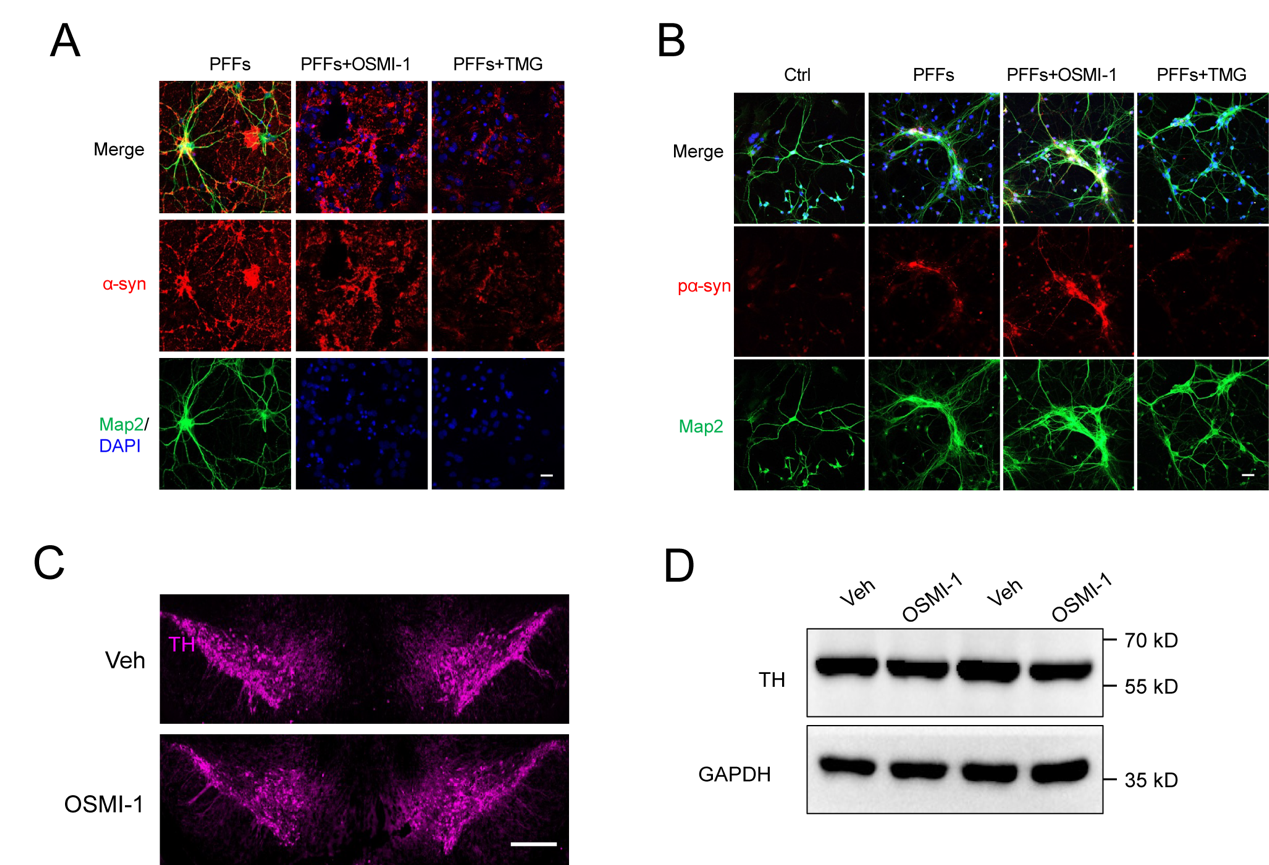


**Fig S4. Modulation of O-GlcNAcylation affects α-syn aggregation after PFFs seeding in primary neurons and cytotoxicity testing of OSMI-1**

(**A-B**) Primary neurons cultured for 7 days were seeded with PFFs (2 μg/ml) and further maintained for one week. Before staining, the cells were treated with OSMI-1 (20 μM) or TMG (1 μM) for two days. Immunofluorescent staining was performed using the neuronal marker MAP2 (green) and antibodies against pan α-syn (A) or pS129 α-syn (p-syn) (B) (red). DAPI (blue) was used to identify the nucleus. Scale bars = 20 μm. (**C**) Immunohistochemical staining of dopaminergic neurons (TH^+^, magenta) in the substantia nigra of mice injected with OSMI-1 (0.4 mg/kg). Scale bar = 500 μm. (**D**) Immunoblot analysis of TH levels in the substantia nigra of OSMI-1-injected mice. GAPDH serves as theloading control.

**Fig S5. Unilateral delivery of pAAV-hSyn-SNCA-eGFP virus and PFFs mixture into substantia nigra in mice midbrain**

(**A**) Amino acid sequence alignment of human (*H. sapiens*) and mouse (*M. musculus*) α, β, γ-synuclein. The sequence with the pale red (1-60), green (61-95), and light blue (96-140) indicates the amphipathic domain, hydrophobic domain, and acidic domain, respectively. Amino acids in identity and differences among the six origins are highlighted in black and red, respectively. (**B**) Schematic illustration of the pAAV-hSyn-*mSNCA*-*eGFP*-WPRE (AAV/PFFs) construct that expresses mouse α-syn (*mSNCA*) under the control of the neuron-specific human *synapsin* promoter. WPRE: Woodchuck hepatitis virus posttranscriptional regulatory element. (**C**) Experimental design for delivery of AAV/PFFs (2 x 10^9^ va) and PFFs (10 μg) mixture into the right substantia nigra of mice. (**D**) Immunoblot analysis of α-syn expression by anti-α/β-syn antibody (sny211) in mice substantia nigra four weeks post-injection. The target band, theoretically ~55 kDa, was recognized. GAPDH as a loading control. (**E**) Immunofluorescence (IF) for visualization of α-syn (green). Dopaminergic neurons were marked by tyrosine hydroxylase (TH, magenta). IF images show that α-syn expression causes loss of dopaminergic neurons (arrowhead) in the ipsilateral substantia nigra at four weeks post injection. Scale bar = 0.5 mm.

**Fig S6. Identification of O-GlcNAcylation sites of α-syn mapped by HPLC-MS/MS**

(**A**) Schematic representation of the α-syn sequence and the identified O-GlcNAcylation sites Ser42, Thr44, Thr54, and Thr59 from α-syn dimer. (**B**) HPLC-MS/MS spectra of glycosylation (G)-modified peptides embedding S42/T44/T54/T59 mapping on the oligomerized α-syn are shown. The sequence of each precursor peptide, the modified residues, and the precursor m/z (+203.0794 Da) are indicated. (**C**) Locations of O-GlcNAclyted residues and amino acid alignment (33-66) of α-syn in various species. NCBI RefSeq accession numbers are as follows: *Homo sapiens*, P37840; *Bos taurus*, NP_001029213.1; *Canis lupus*, XP_860942.1; *Mus musculus*, NP_001035916.1; *Rattus norvegicus*, NP_062042.1; *Danio rerio*, NP_001017567.1. Identity (red) and similarity (blue) of O-GlcNAcylated serine (S) and threonine (T) residues, as well as conserved amino acids (gray background), are shown.


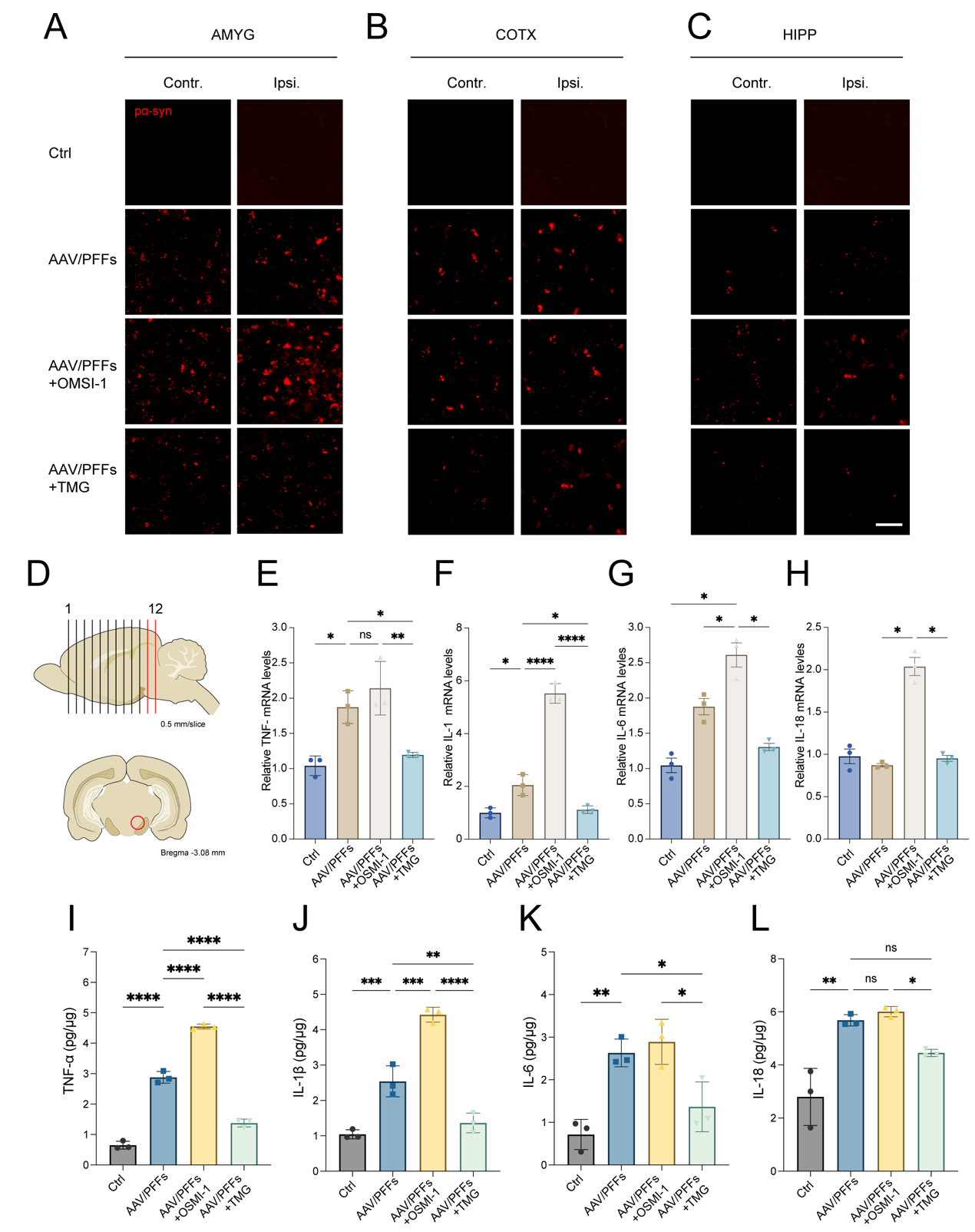


**Fig S7. Modulation of O-GlcNAc affects microglial activity and cytokine presentation elicited by α-syn aggregation in mouse brain**

(**A-C**) Immunohistochemistry analysis of p-syn in the ipsilateral and contralateral sides of the amygdala (A), cortex (B), and hippocampus (C) from mice that had a unilateral substantia nigra AAV/PFFs injection. Scale bar=25 μm. (**D**) Schematic demonstrating the location of the dissected regions in coronal section slices, based on the Acrylic Alto Matrix model of the adult mouse brain with 12 grooves at intervals of 0.5 mm (upper). The lower image shows a coronal section cut through the substantia nigra on the ipsilateral side of the AAV/PFFs injection. The slice was further dissected (red circle) and used for the ELISA assay. (**E-H**) mRNA levels of *TNF-α* (E), *IL-1β* (F), *IL-6* (G), and *IL-18* (H) in mice brain samples. The relative mRNA levels were normalized to the *GAPDH* (n=3). (**I-L**) Levels of TNF-α (I), IL-1β (J), IL-6 (K), and IL-18 (L) in 200 μg/mL of total proteins obtained from the mice midbrains were detected using ELISA (n=3). Values are presented as means ± SEM. *P<0.05, **P<0.01, ***P<0.001, and ****P<0.0001 by one-way ANOVA with Tukey’s post-hoc test.

**Fig S8. Donated SH-SY5Y cells treated with α-syn downregulate O-GlcNAc and upregulate NLRP3 in the recipient BV-2 cells**

(**A-E**) The cell culture medium of donated SH-SY5Y treated with conformational α-syn was obtained, the secreted α-syn was depleted by the anti-α-syn antibody (see material and methods), and the medium was used to treat BV-2 cells. Immunoblot and quantification analysis evaluated the O-GlcNAc, OGT, OGA, and NLRP3 protein levels. GAPDH as a loading control (n=4). (**F-I**) Quantification of O-GlcNAc (F), OGT (G), OGA (H), and NLRP3 (I) protein levels in BV-2 cells directedly treated with conformational strains of α-syn. The protein levels were normalized to GAPDH (n=3). (**J**) Immunoblot analysis of O-GlcNAcylation, OGT, and OGA in the PFFs-seeded BV-2 cells treated with OSMI-1 or TMG. GAPDH as a loading control. (**K, L**) Conditional medium (CM) from SH-SY5Y was seeded with various doses of PFFs and used to culture BV-2 cells for 24 h. Immunoblot analysis was performed to evaluate the levels of NLRP3 in recipient cells. Values are presented as means ± SEM. *P<0.05, **P<0.01, and ***P<0.001, by one-way ANOVA with Tukey’s post-hoc test.

**
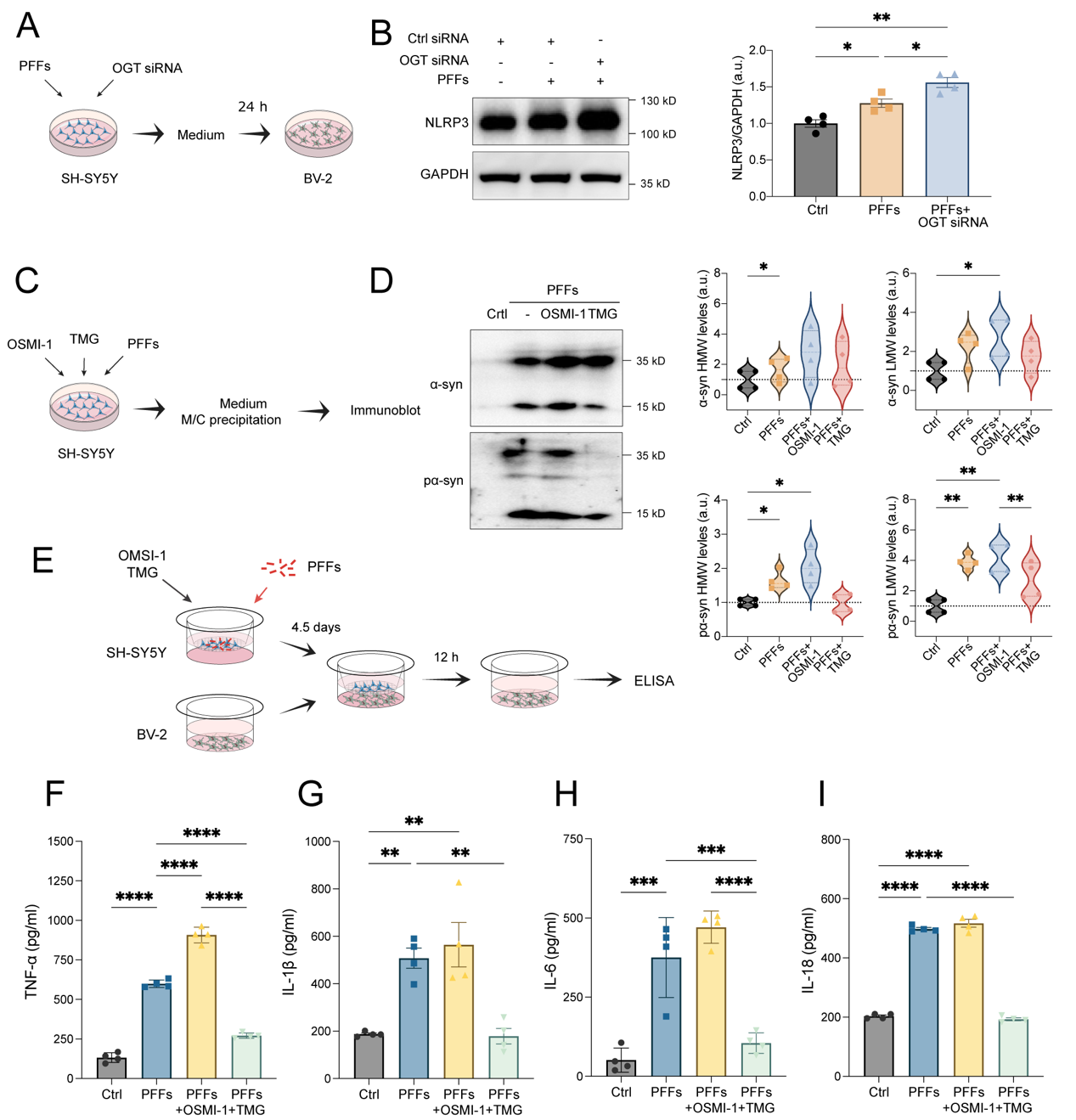
**

**Fig S9. Regulating O-GlcNAcylation triggers inflammatory cytokine secretion**

(**A**) The culture medium obtained from SH-SY5Y cells transfected with OGT siRNA for 36 h was seeded with PFFs for 3 days and then treated with BV-2 cells for 24 h. (**B**) The BV2 lysate was used to perform the immunoblot, and the quantification of NLRP3 levels was normalized to GAPDH (n=4). (**C**) Precipitation of the secreted proteins from the cultured medium of PFFs-seeded SH-SY5Y cells treated with OSMI-1 or TMG using a methanol/chloroform (M/C) method. (**D**) Immunoblot and quantification analysis for the high and low molecular mass of α-syn or pS129 α-syn in the M/C precipitated proteins. (**E**) Schematic design of the neuron-glial co-culture procedure for ELISA assay. PFFs seeded SH-SY5Y were treated with OSMI-1 or TMG for 4.5 days and co-cultured with BV-2 cells for 12 hours following a medium change. The co-cultured medium was obtained and used for ELISA. (**F-I)** The concentrations of TNF-α (F), IL-1β (G), IL-6 (H), and IL-18 (I) in the co-cultured cell medium were measured by ELISA (n=4). Values are presented as means ± SEM. *P<0.05, **P<0.01, ***P<0.001, and ****P<0.0001 by one-way ANOVA with Tukey’s post-hoc test.

**
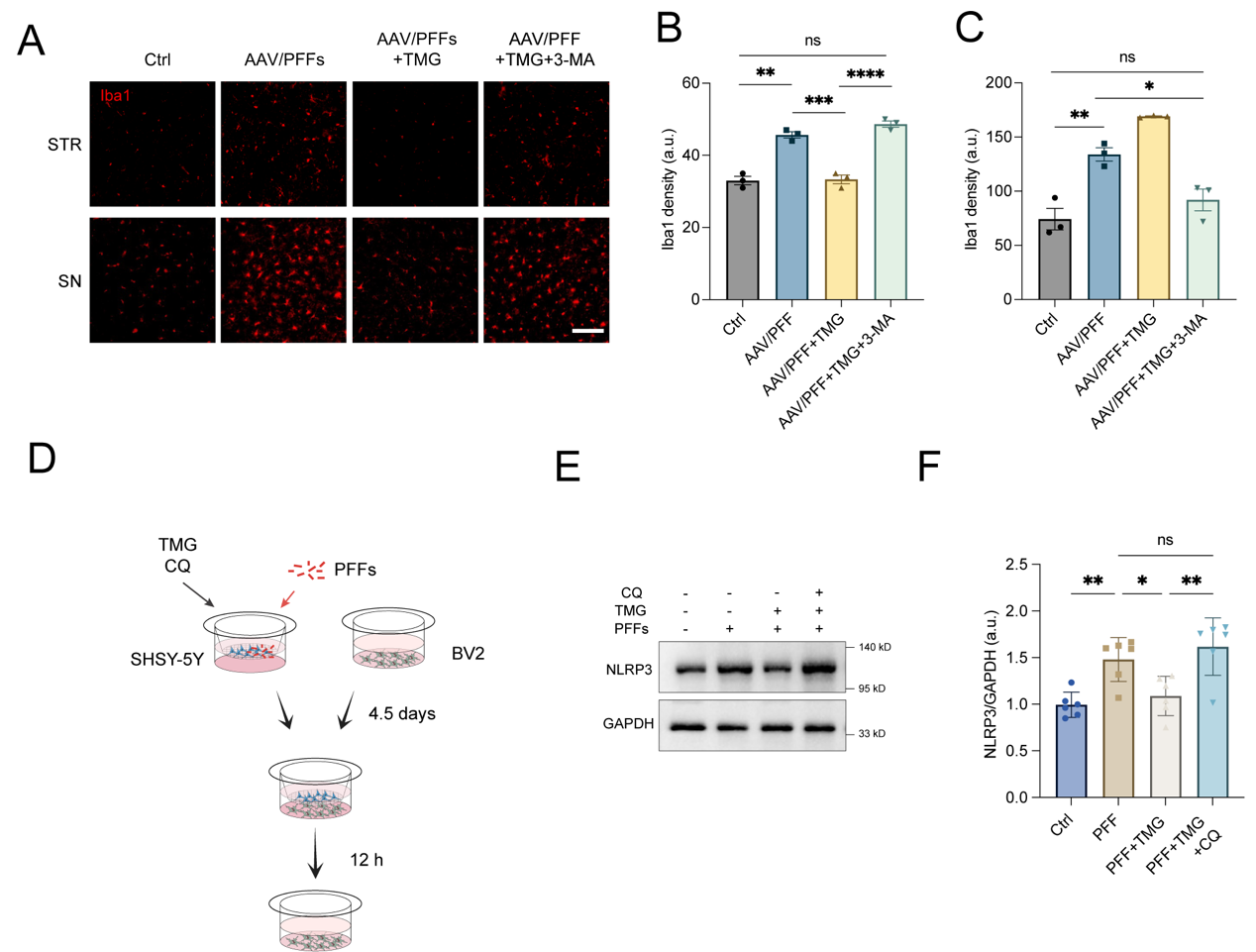
**

**Fig S10. Inhibiting autophagosome-lysosome flux restrains pathological α-syn-induced microglial inflammation**

(**A**) Representative immunofluorescent images for Iba1^+^ microglia of the substantia nigra and striatum in the brain of AAV/PFFs mouse treated with TMG and 3-MA. Scale bar = 100 μm. (**B-C**) Quantification of Iba1^+^ cells in substantia nigra (B) and striatum (C) in the mouse brains (n=3). (**D**) Schematic diagram of the trans-well co-culture experimental design. Before treatment with 40 μg/ml PFFs for 4.5 days, SH-SY5Y cells were pre-incubated with 10 μM chloroquine (CQ) and 1 μM TMG. Then, the compound and PFFs were washed out by changing the culture medium. The cells were subjected to immunoblot analysis after co-culturing with BV-2 cells for an additional 12 h. (**E**) Immunoblot analysis for NLRP3 in BV-2 cells of trans-well co-culture experiments. **(F)** The graphs depict the densitometric quantification of NLRP3 levels normalized to GAPDH (n=6). Values are presented as means ± SEM. *P<0.05, **P<0.01, ***P<0.001, and ****P<0.0001 by one-way ANOVA with Tukey’s post-hoc test.

**Fig S11. Blocking autophagosome-lysosome flux or the proteasome does not influence the PFFs-seeding-induced OGT reduction**

(**A**) Immunoblot analysis for O-GlcNAc proteins, OGT, and OGA in SH-SY5Y cells treatment of PFFs combined with TMG and 3-MA or chloroquine (CQ). GAPDH was used as a loading control. (**B**) Immunoblot analysis for O-GlcNAc proteins, OGT, and OGA in SH-SY5Y cells treated with PFFs combined with TMG in the presence or absence of MG-132. GAPDH was used as a loading control.

**Fig S12. Scheme of O-GlcNAcylation cycling modulates α-syn aggregation and cell-to-cell transmission, correlating with pathology manifestation and proteolysis degradation**

The α-syn fibrillar species recruits monomeric α-syn to form high molecular mass aggregates and pathologically transmits them to neighboring cells or anatomical connecting regions. Reduced O-GlcNAcylation by restricting glucose supply or enzymatic inhibition of OGT promotes α-syn aggregation-induced pathology, including dopaminergic neuronal toxicity and neuroinflammation. Conversely, inhibiting OGA or increasing O-GlcNAc substrate donation ameliorates pathological α-syn aggregation and transmission and NLRP3-related microglial activation. Additionally, elevated O-GlcNAcylation improves α-syn aggregate degradation through autophagosome-lysosome proteolysis. Therefore, pathological α-syn aggregation, transmission, and consequently, microglial inflammation can be blocked by O-GlcNAcylation.

| **PTM sites** | **Positions in Proteins** | **Sites** |
| --- | --- | --- |
| **O-GlcNAc site (g)** | |  |
| EGVLYVGSK | 35-43 | Ser42 |
| EGVLYVGSKTKEGVVHGVATVAEKTK | 35-60 | Thr44; Thr54; Thr59 |
| EGVVHGVATVAEK | 46-58 | Thr54 |
| **Phosphorylation sites (p)** |  |  |
| EGVVAAAEKTKQGVAEAAGKTK | 13-34 | Thr22 |
| QGVAEAAGKTKEGVLYVGSKTK | 24-45 | Thr33 |
| **Ubiquitination (u)** |  |  |
| EGVVAAAEKTKQGVAEAAGKTK | 13-34 | Lys32 |
| QGVAEAAGKTKEGVLYVGSKTK | 24-45 | Lys34; Lys43; Lys45 |
| **Oxidation (ox)** |  |  |
| MDVFMK | 1-6 | Met1; Met5 |

**Table S1. Post-translational modification (PTM) identification of α-syn on PFFs seeding in vitro via HPLC-MS/MS**

**Table S2. List of antibodies used in the study**

| Antigen | Supplier | Product catalog | Dilution used in WB | Dilution used in IHC/ICC |
| --- | --- | --- | --- | --- |
| α-synuclein (MJFR1) | Abcam | ab138501 | 1:1000 | 1:600 |
| α-synuclein (LB509) | Abcam | ab27766 | 1:1000 | 1:600 |
| α/β-synuclein (Syn205) | CST | 2644 | 1:1000 | 1:100 |
| α-synuclein (Syn211) | Invitrogen | AHB0261 | N/A | 1:100 |
| α/β-synuclein (R04-5C4) | ZEN Bio | R380962 | 1:1000 |  |
| P129S-α-synuclein | Abcam | ab51253 | 1:1000 | 1:600 |
| P129S -α-synuclein | ZEN Bio | R380937 | N/A | 1:200 |
| Tyrosine hydroxylase | Abcam | Ab76442 | N/A | 1:1000 |
| Tyrosine hydroxylase | Novus | NB300-109 | 1:1000 | N/A |
| GAPDH | Proteintech | 60004-1 | 1:5000 | N/A |
| O-GlcNAc | PTM, Bio | PTM-051 | 1:1000 | N/A |
| O-GlcNAc, RL2 | Invitrogen | MA1072 | 1:1000 | N/A |
| OGT | Abcam | ab96718 | 1:1000 | N/A |
| OGA/MGEA5 | Proteintech | 14711-1-AP | 1:1000 | N/A |
| Oligomer A11 | Invitrogen | AHB0052 | 1:1000 | N/A |
| β-actin | Beyotime | AF0003 | 1:1000 | N/A |
| MAP2 | Invitrogen | MA5-12826 | N/A | 1:500 |
| Iba1 | Oasisbiofarm | OB-PGP049 | N/A | 1:1000 |
| β-tubulin | Fdbio | FD0064 | 1:1000 | N/A |
| NLRP3 | CST | 15101 | 1:1000 | N/A |
| p62/SQSTM1 | MBL | PM-045 | N/A | 1:200 |
| Ubiquitin K-48 | Sigma-Aldrich | ZRB2150 | 1:1000 | 1:100 |
| Ubiquitin K-63 | ZEN Bio | R381564 | 1:1000 | 1:100 |
| Lamp1 | CST | 9091 | N/A | 1:100 |
| Goat anti-Mouse IgG (H+L) secondary antibody, Alexa Fluor 488 | Invitrogen | A-11017 | N/A | 1:500 |
| Goat anti-Mouse IgG (H+L) Highly secondary antibody, Alexa Fluor 594 | Invitrogen | A-10037 | N/A | 1:500 |
| Dylight 649 AffiniPure Goat anti-Rabbit IgG (H+L) | Fdbio | FD0130 | N/A | 1:500 |
| Goat anti-Chicken IgY (H+L) secondary antibody, Alexa Fluor 647 | Abcam | ab150171 | N/A | 1:1000 |
| Goat anti-Guinea pig IgG (H+L), 594 | Oasisbiofarm | G-GP594 | N/A | 1:1000 |

N/A: non-applicable

**Table S3. Sequences of primers used for RT-qPCR analysis of mouse brain tissue**

| Species | Genes | Primer | Nucleotide sequence |
| --- | --- | --- | --- |
| Mus musculus | *TNF-α* | F | ACTCCAGGCGGTGCCTATAT |
|  |  | R | GTGAGGGTCTGGGCCATAGAA |
| Mus musculus | *IL-6* | F | CCACTTCACAAGTCGGAGGCTTA |
|  |  | R | GCAAGTGCATCGTTGTTCATAC |
| Mus musculus | *IL-1β* | F | TGGTGTGTGACGTTCCCATT |
|  |  | R | TCGTTGCTTGGTTCTCCTTG |
| Mus musculus | *IL-18* | F | TGGTTCCATGCTTTCTGGACTCCT |
|  |  | R | TTCCTGGGCCAAGAGGAAGTGATT |
| Mus musculus | *actin* | F | CATTGCTGACAGGATGCAGAAGG |
|  |  | R | TGCTGGAAGGTGGACAGTGAGG |

F, forward; R, reverse
